# Lysine targeting covalent inhibitors of malarial kinase *Pf*CLK3†

**DOI:** 10.1039/d5md00335k

**Published:** 2025-05-27

**Authors:** Skye B. Brettell, Gillian Cann, Abbey Begen, Saumya Sharma, Amit Mahindra, Lauren V. Carruthers, Graeme Milligan, David J. Clarke, Andrew B. Tobin, Andrew G. Jamieson

**Affiliations:** a School of Chemistry, The Advanced Research Centre, University of Glasgow 11 Chapel Lane Glasgow G11 6EW UK andrew.jamieson.2@glasgow.ac.uk; b Centre for Translational Pharmacology, The Advanced Research Centre, University of Glasgow 11 Chapel Lane G11 6EW UK; c Keltic Pharma Therapeutics, The Advanced Research Centre, University of Glasgow 11 Chapel Lane G11 6EW UK; d EaSTCHEM School of Chemistry, University of Edinburgh Joseph Black Building, David, Brewster Road Edinburgh EH9 3FJ UK

## Abstract

Malaria continues to devastate tropical regions of the world, with resistance to frontline drugs on the rise. Kinase inhibition has emerged as a promising novel mechanism of action in the fight against malaria. We previously reported the development of TCMDC-135051 (1), a highly potent, multi-stage inhibitor of *Plasmodium falciparum* CLK3 (*Pf*CLK3). Building on this work, we subsequently developed the first covalent kinase inhibitor for malaria, selectively targeting a unique cysteine residue. Despite their high potency and selectivity, covalent inhibitors that target cysteine residues are particularly vulnerable to resistance arising from single point mutations of the nucleophilic residue. This work presents a novel strategy targeting the essential kinase catalytic lysine residue which has the potential to evade this resistance mechanism. Using structure based drug design, analogues of TCMDC-135051 (1) targeting Lys394 of *Pf*CLK3 were developed. Four compounds, all harbouring benzaldehyde-based warheads, covalently engaged Lys394 as determined by protein mass spectrometry. These analogues were highly potent against recombinant protein, with good parasiticidal potency and cytotoxicity profiles. These molecules 4, 5, 8, 9 are the first lysine-targeting covalent inhibitors reported for malaria and offer a promising general strategy for future antimalarial drug discovery.

## Abstract 2

A series of covalent *Pf*CLK3 inhibitors targeting catalytic Lys394 were designed, synthesised, and evaluated as antimalarials, providing a novel strategy to combat malaria resistance.

## Graphical abstract

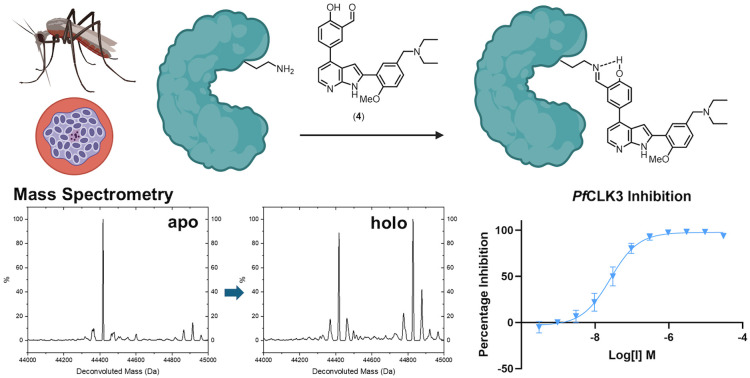

## Introduction

Malaria persists in its devastation of tropical developing regions, with approximately 600 000 deaths annually.^1^ In 2023, 76% of these deaths were of children under 5. With each novel therapeutic or insecticide comes new resistant strains of *Plasmodium falciparum* (*P. falciparum*), limiting therapeutic efficacy.^2^ After the development of chloroquine in 1934, resistance was first detected in in 1957.^3^ After sulfadoxine-pyrimethamine combination treatment was introduced in 1967 to replace chloroquine, resistance was detected later that year.^4^ A similar story is true of other frontline treatments such as proguanil and mefloquine.^5,6^ The release of artemisinin in the 1980 and 1990s brought new hope of eradication, and a rapid reduction in malaria cases and mortality.^7^ However, by 2008 strains of *P. falciparum* resistant to artemisinin were detected in Cambodia.^8^ Since then this resistance has spread to parts of Africa.^9^ The efficacy of artemisinin combination therapy (ACT) has now been compromised, threatening the progress that has been made in the last 20 years. The WHO's aspirational target of malaria eradication by 2030 is now highly unlikely to be met.^1^

Novel therapeutics with new mechanisms of action are therefore required to combat these resistant strains. We have validated an essential *P. falciparum* kinase *Pf*CLK3 as a novel drug target using our tool inhibitor TCMDC-135051 (1).^10^ Inhibition of *Pf*CLK3 could prove a therapeutic strategy to deliver a transmission blocking, prophylactic and curative agent.^11^ In an effort to improve the potency and selectivity of TCMDC-135051 (1), we have since developed a covalent kinase inhibitor targeting a cysteine not conserved across the human kinome.^12^ This compound showed improved potency, duration of action and selectivity relative to TCMDC-135051 (1), validating our strategy of developing a covalent inhibitor of this kinase.

Acquired resistance towards cysteine-targeting covalent inhibitors by single-point Cys to Ser mutations have been observed in the field of oncology, however.^13–17^ For this reason, several covalent inhibitors targeting other residues are currently in pre-clinical development.^18^ Kinase inhibitors in particular have been developed targeting the catalytic lysine of the ATP binding site.^19^ This residue is conserved across all protein kinases, and is involved in the phospho-transfer from ATP to the substrate.^20^ In 2017, Dalton *et al.* demonstrated for the first time that the catalytic lysine of PI3Kδ could be selectively targeted with amine-reactive covalent inhibitors.^21^ Since then, multiple kinase drug targets have been inhibited using this approach.^18,19^ This strategy should be unsusceptible to single-point mutations of the nucleophile, evading resistance by this mechanism. In this way inhibitors that operate *via* covalent modification of the catalytic lysine of protein kinases could categorised as “irresistible”. We therefore propose this as a strategy worthwhile investigating in the field of malaria.

As a first step in testing this hypothesis, we aimed to determine if it were possible to generate covalent inhibitors targeting the catalytic lysine of the essential protein kinase, *Pf*CLK3 (Lys394). In this work, several inhibitors were designed based on a scaffold of TCMDC-135051 (1). Some of which, 4, 5, 8 and 9, showed excellent biochemical potency, and covalent adduct formation *via* protein mass spectrometry. Inhibitors maintained moderate parasiticidal potency from this change of mechanism. These results demonstrate, for the first time, that the catalytic lysine of *Pf*CLK3 can be targeted by covalent inhibitors.

## Structure-based drug design

**Fig. 1:**
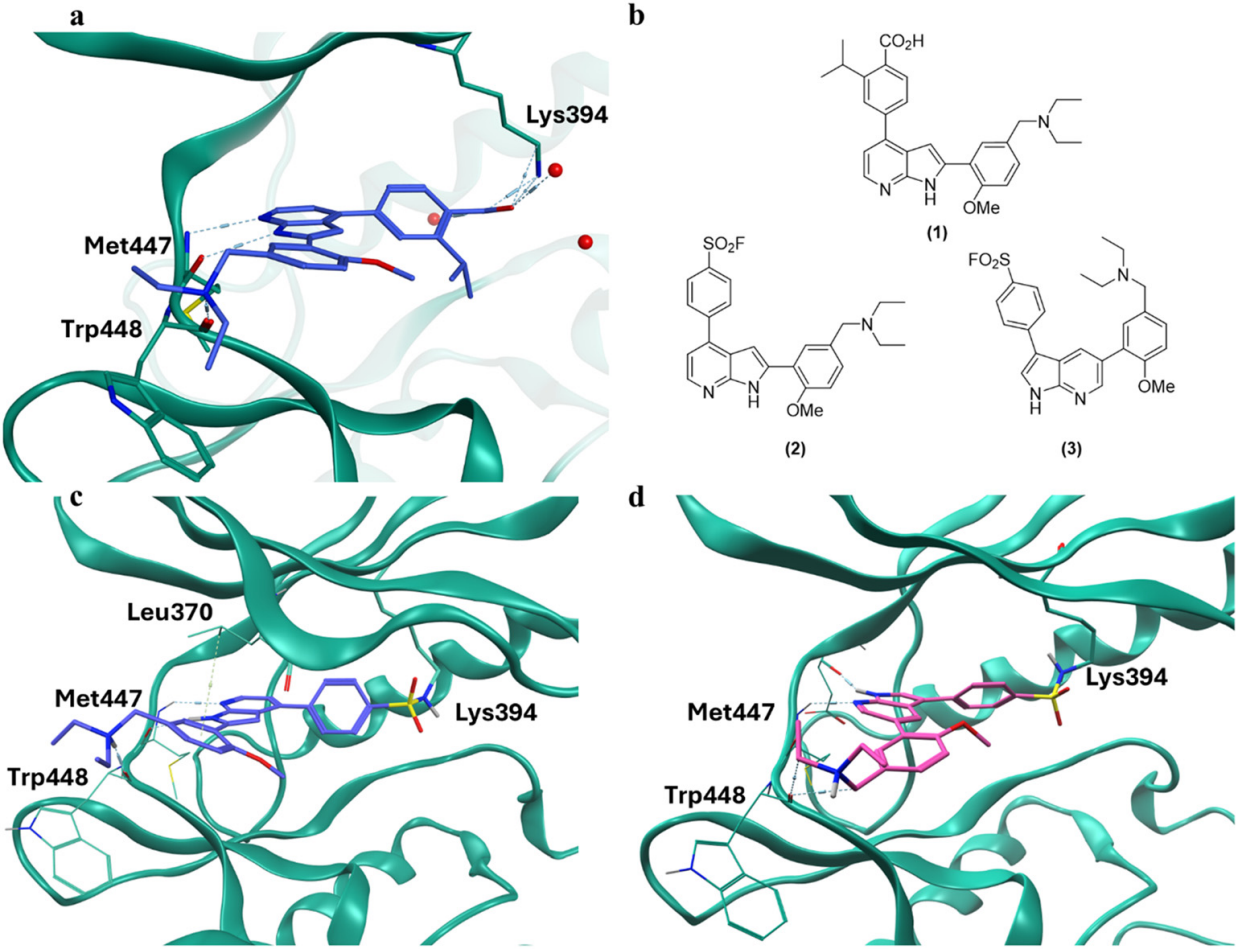
a, Previously published co-crystal structure of TCMDC-135051 in *Pf*CLK3 (PDB: 8RPC); b, structures of TCMDC-135051, compound 2 and 3; c, and d, molecular docking of compounds 2 and 3 respectively in the co-crystal structure.

We recently published a crystal structure of *Pf*CLK3 in complex with TCMDC-135051 (8RPC, Fig. 1a), which shows the benzoic acid of the ligand to for a salt bridge with catalytic Lys394 .^12^ We therefore sought to replace this benzoic acid with various amine-reactive groups. Sulfonyl fluorides have been shown to target a broad range of human kinases, which use sulfur(vi) fluoride exchange (SuFEX) chemistry to covalently bind the amine side chain of the catalytic lysine.^19,22,23^ Direct replacement of the benzoic acid of TCMDC-135051 (1) with a sulfonyl fluoride yielded compound 2 (Fig. 1b). The 3,5-substituted indole regioisomer 3 was also designed (Fig. 1b), inspired by examples in the literature targeting the catalytic lysine of kinases using this scaffold.^24^ These compounds were then docked into the co-crystal structure using the molecular operating environment's (MOE) covalent docking tool (Fig. 1c and d). Both regioisomers were predicted to bind in the ATP binding site and form a covalent bond after the SuFEX reaction with Lys394. Compound 2 appears to maintain the “flipped” azaindole binding-mode of TCMDC-135051 (1), while compound 3 binds in the “normal” binding-mode and forms hydrogen bonds with the hinge residues Met447 and Glu445 (Fig. 1e and f). This is consistent with the expected binding mode of the 3,5 substituted azaindoles in other kinase inhibitors.^25,26^ The interaction of the diethylamine and Trp448 in the TCMDC-135051-bound structure was also predicted to be maintained in compound 2.

**Fig. 2:**
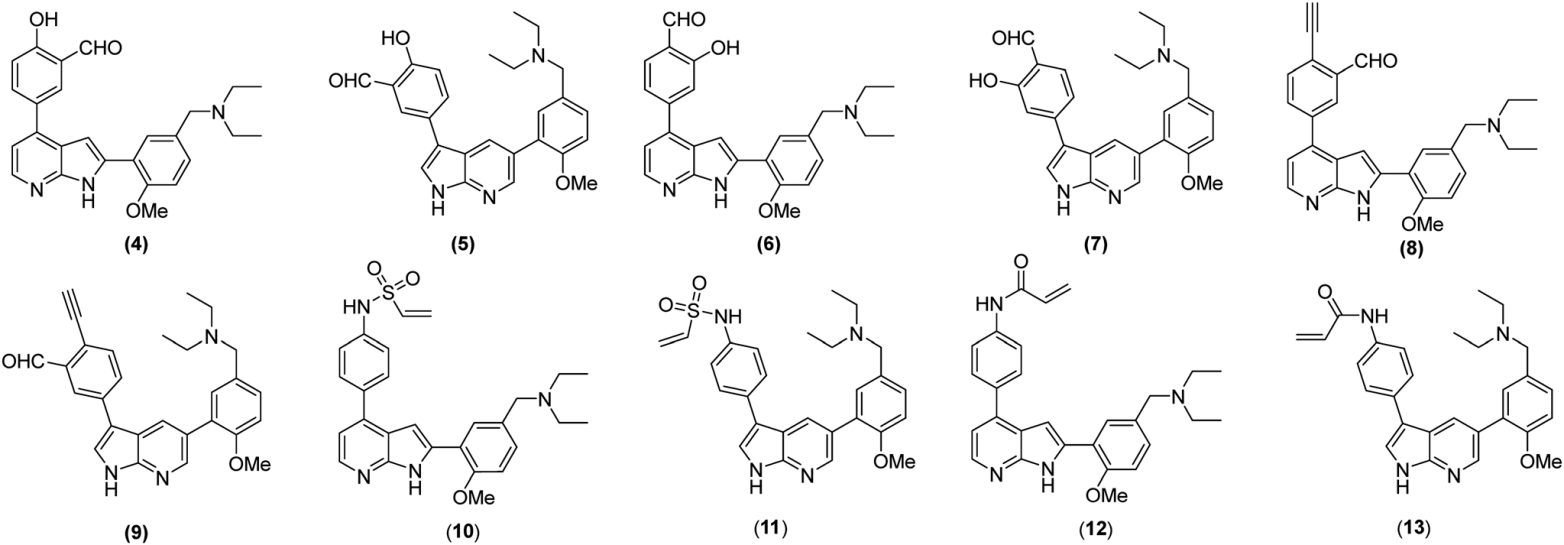
Designed covalent inhibitors 4–13 featuring salicylic aldehyde, ethynyl benzaldehyde, vinyl sulfone and acrylamide warheads.

Sulfonyl fluorides can prove unstable to hydrolysis and to human metabolism however.^27^ We therefore sought to diversify our library of potential covalent inhibitors by replacing the benzoic acid with a range of more stable warheads. Salicylic aldehydes have been reported in the literature to covalently bind globulins, kinases and regulator proteins. Voxelotor was approved to treat sickle cell disease in 2019.^28^ It uses a salicylic aldehyde to form a reversible Schiff-base linkage to the N-terminal valine of mutant haemoglobin.^29^ More recently, Chen *et al.* used this warhead to form the stabilised imine with Lys271 of BCR-ABL kinase, producing a cell active, selective inhibitor.^24^ Two regioisomers (compounds 4 and 5, Fig. 2) were therefore designed, maintaining the optimal substitution found in the azaindole inhibitors of BCR-ABL. Taunton and coworkers showed both *para*- and *meta*-substituted salicylic aldehydes could covalently bind >95 kinases in Jurkat cells, thus compounds 6 and 7 were designed.^30^ Irreversible ethynyl benzaldehyde-based inhibitors 8 and 9 were also designed, inspired by the work of Chen *et al.* who developed ethynyl benzaldehyde covalent inhibitors of BCR-ABL, EGFR and Mcl-1.^31^ These reactive groups first form an imine with the catalytic lysine, followed by a tandem cyclisation event with the alkyne to form an irreversible pyridinium adduct. In this work by Chen *et al.*, ethynyl benzaldehyde inhibitors showed superior potency to those harbouring salicylic aldehydes. Vinyl sulfonamides have also demonstrated promising reactivity with lysine residues, while acrylamides, which share the geometric features of sulfonamide warheads, offer the added advantage of improved metabolic stability.^19,32,33^ Sulfonamides 10–11 and acrylamides 12–13 were therefore designed. Molecular docking of all analogues was attempted, however limitations of the software meant these warheads were not compatible with the covalent molecular docking tool in MOE. This highlights limitations that are often encountered using current molecular docking capabilities with covalent inhibitors.

### Synthesis

**Scheme 1:**
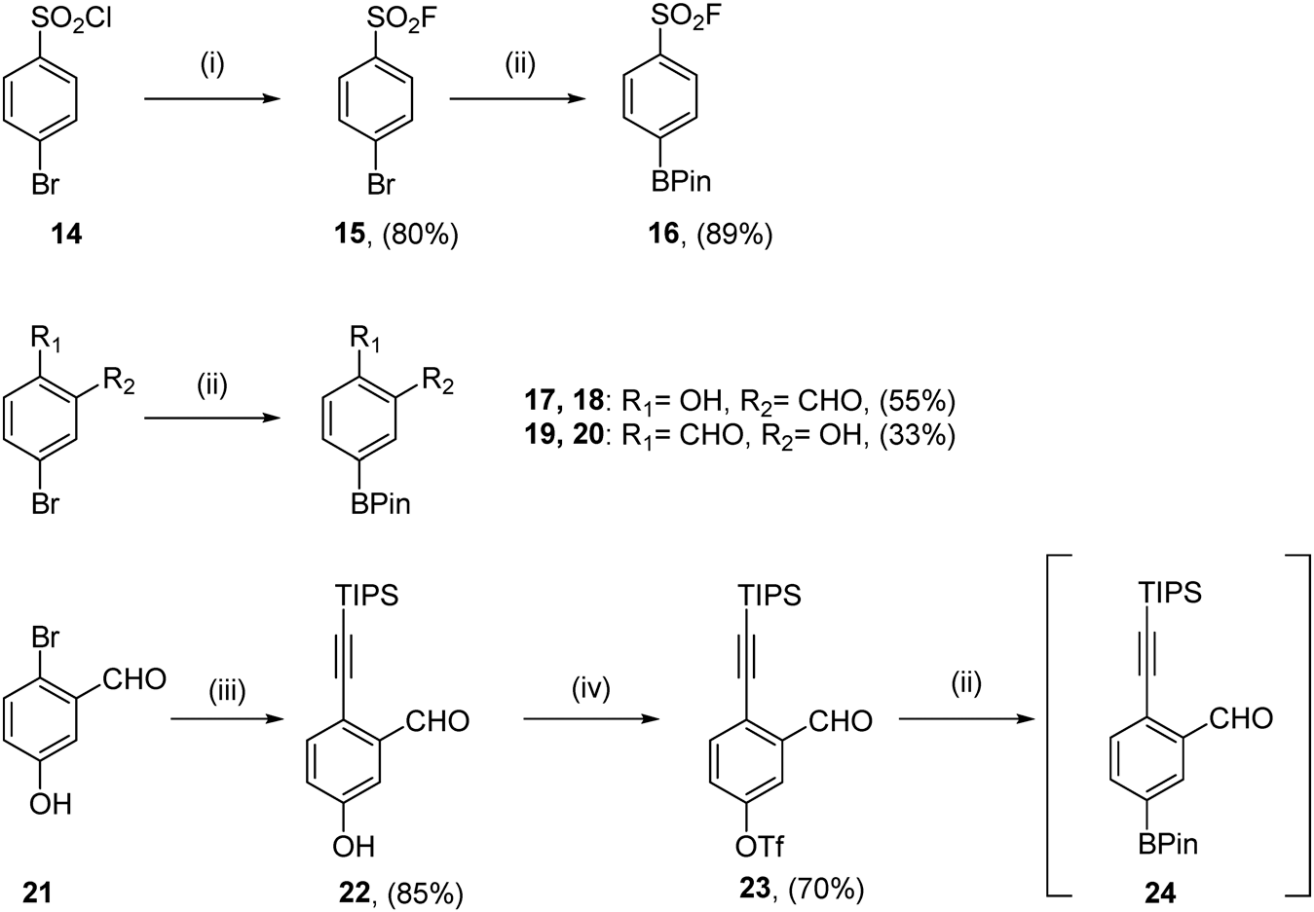
Synthesis of aryl warheads as boronic esters. (i) KHF_2_, H_2_O, MeCN, rt, 24 h; (ii) bis(pinacolato)diboron, KOAc, Pd(dppf)Cl_2_·CH_2_Cl_2_ (10 mol%), 1,4-dioxane, 80 °C, 18 h; (iii) Pd(dppf)Cl_2_·CH_2_Cl_2_, CuI, triisopropylsilylacetylene, NEt_3_, 50 °C, 3 h; (iv) trifluoromethanesulfonyl chloride, NEt_3_, CH_2_Cl_2_, 0 °C – rt, 2 h.

For all compounds, aryl motifs bearing warheads at one end and boronic esters at the other were synthesised (Scheme 1). Sulfonyl fluorides were synthesised from commercially available starting material 14 according to the protocol published by Lou and Willis.^34^ Halogen exchange yielded sulfonyl fluoride 15, before a Suzuki–Miyaura cross-coupling to install the boronate ester of 16 in good yield. Salicylic aldehyde coupling partners were synthesised from commercially available bromides using Suzuki–Miyaura cross-couplings giving 18 and 20 in moderate yields. TIPS protected ethynyl benzaldehyde coupling partners were obtained using the 3 step protocol outlined by Chen *et al.*^31^ A Sonogashira-coupling furnished alkyne 22 in 85% yield, followed by the installation of a triflate to give 23. Borylation of this triflate yielded crude material which was unstable to silica-based column chromatography, so compound 24 was progressed to coupling reactions without further purification.

**Scheme 2:**
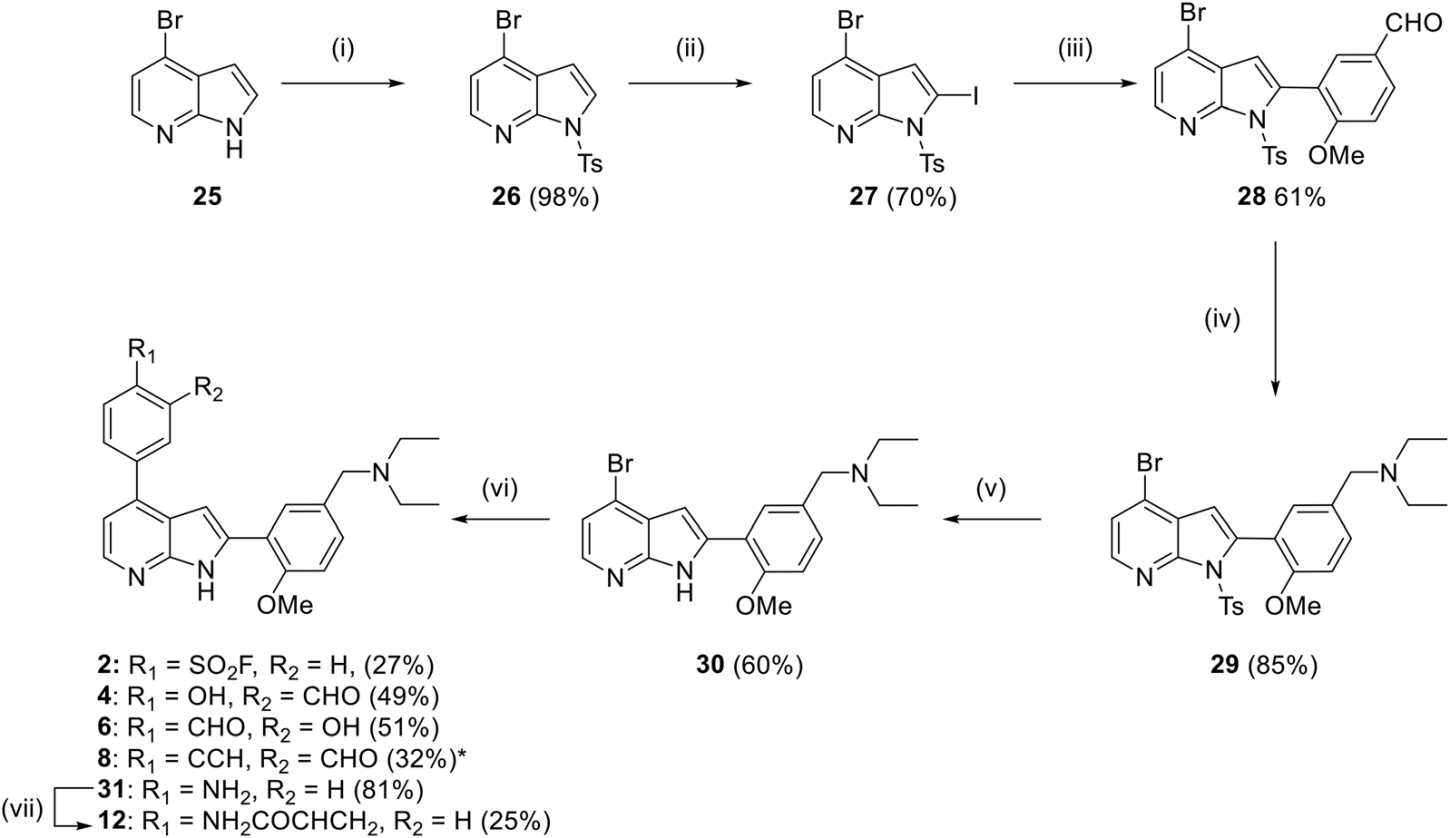
Synthesis of compounds 2, 4, 6 and 8. (i) TsCl, NaH, THF, 0 °C, 2 h; (ii) LDA, I_2_, THF, −78 °C, 3 h; (iii) (5-formyl-2-methoxyphenyl)boronic acid, Pd(PPh_3_)_4_ (10 mol%), Na_2_CO_3_, 1,4-dioxane, Δ, 12 h; (iv) diethylamine, NaBH(AcO)_3_, 1,4-dioxane, rt, 12 h; (v) CH_3_OH, KOH, Δ, 18 h; (vi) boronic ester, Pd(dppf)Cl_2_·CH_2_Cl_2_ (5 mol%), Na_2_CO_3_, 1,4-dioxane, Δ, 0.5 h, μW; (vii) acryloyl chloride, DIPEA, DMF, rt, 2 h. *Obtained using conditions (vi) followed by treatment with CsF in DMF, rt, 0.5 h.

Synthesis of compounds 2, 4, 6, 8 and 11 was achieved using the previously published synthesis of TCMDC-135051 to obtain common intermediate 30 (Scheme 2).^11^ Tosylation of commercially available 4-bromoazaindole 25 produced azaindole 26 in 98% yield. Selective iodination of the indole C-2 position was achieved through directed metalation to provide iodide 27, which was obtained 70% yield. Suzuki–Miyaura cross-coupling of the iodo-azaindole with 5-formyl-2-methoxyphenyl boronic acid gave aldehyde 28. Reductive amination installed the diethylamine in 85% yield. Indole deprotection in basic conditions afforded NH-indole 30 in 60% yield. Late-stage diversification using a series of Suzuki–Miyaura cross-couplings yielded final molecules 2, 4, 6 and 8. Aniline 31 was also obtained *via* this method. Standard conditions used for the installation of the benzoic acid of TCMDC-135051 were used in every case except analogue 2, whereby the equivalents of base was reduced, and potassium phosphate was used instead of sodium carbonate. This was to reduce the hydrolysis of the sulfonyl fluoride, which was observed using standard conditions. Treatment of 31 with acryloyl chloride and triethylamine yielded acrylamide 11. The vinyl sulfonamide 10 proved unstable to silica gel chromatography, and was not isolated. It should also be noted that while all final compounds were purified using reverse phase column chromatography, compounds 2 and 8 did not tolerate eluents spiked with 0.1% TFA, and 0.1% acetic acid was used instead. The weaker acid successfully limited hydrolysis and decomposition. All final compounds were obtained in 25–51% yield in purity >95%.

**Scheme 3:**
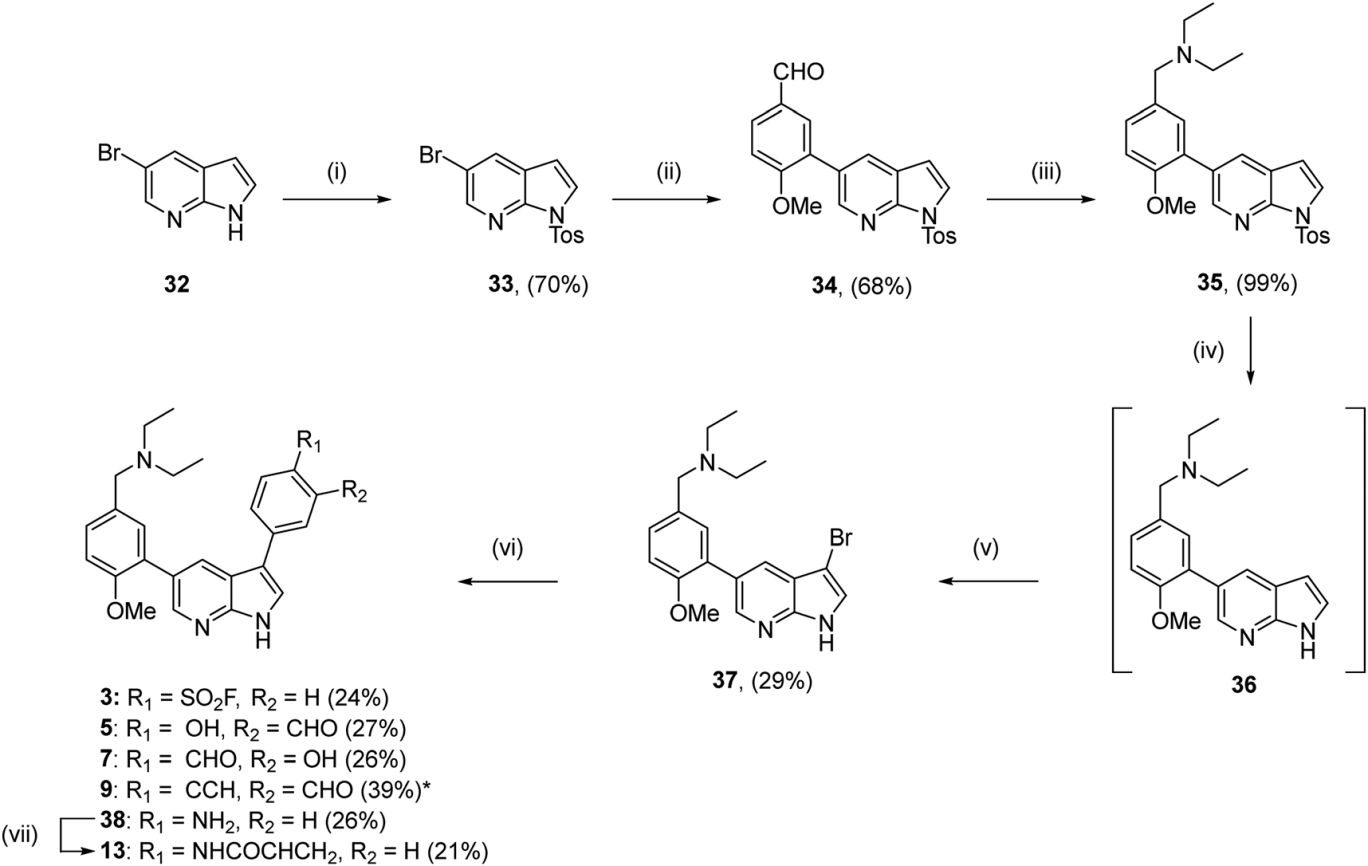
Synthesis compounds 3, 5, 7 and 9. (i) TsCl, NaH, THF, 0 °C, 2 h; (ii) (5-formyl-2-methoxyphenyl)boronic acid, Pd(PPh_3_)_4_ (10 mol%), Na_2_CO_3_, 1,4-dioxane, Δ, 12 h; (iii) diethylamine, NaBH(AcO)_3_, 1,4-dioxane, rt, 12 h; (iv) CH_3_OH, KOH, reflux, 18 h; (v) NBS, CH_2_Cl_2_, rt, 2 h; (vi) boronic ester, XPhos Pd G2 (5 mol%), Na_2_CO_3_, 1,4-dioxane, Δ, 0.5 h, μW; (vii) acryloyl chloride, DIPEA, DMF, rt, 2 h. *Obtained using conditions (vi) followed by treatment with CsF in DMF, rt, 0.5 h.

For the synthesis of the 3,5-substituted azaindoles 3, 5, 7, 9 and 13, a similar synthetic route was followed (Scheme 3). Tosylation of 5-bromo-azaindole served to ease the purification of subsequent compounds, rather than facilitating halogenation as with the 2,4-regioisomers. Suzuki–Miyaura cross-coupling to provide aldehyde 34 and subsequent reductive amination gave diethylamine 35 in 68% and 99% yields respectively. The tosyl group was then removed and the 3-position was brominated using *N*-bromo-succinimide. Bromination did not proceed when the tosyl group was present. Bromide 37 was obtained in poor yield due to its highly polar nature making purification challenging. Compounds 3, 5, 7, 9 and 38 were then obtained under similar conditions used for compounds 2, 4, 6, 8 and 31, with the key modification being the use of XPhos Pd G2 as the catalyst. This change was made after poor conversion was observed using Pd(dppf)Cl_2_·CH_2_Cl_2_. Acrylamide 13 was then obtained through acetylation of amine 38.

### Protein mass spectrometry

**Fig. 3:**
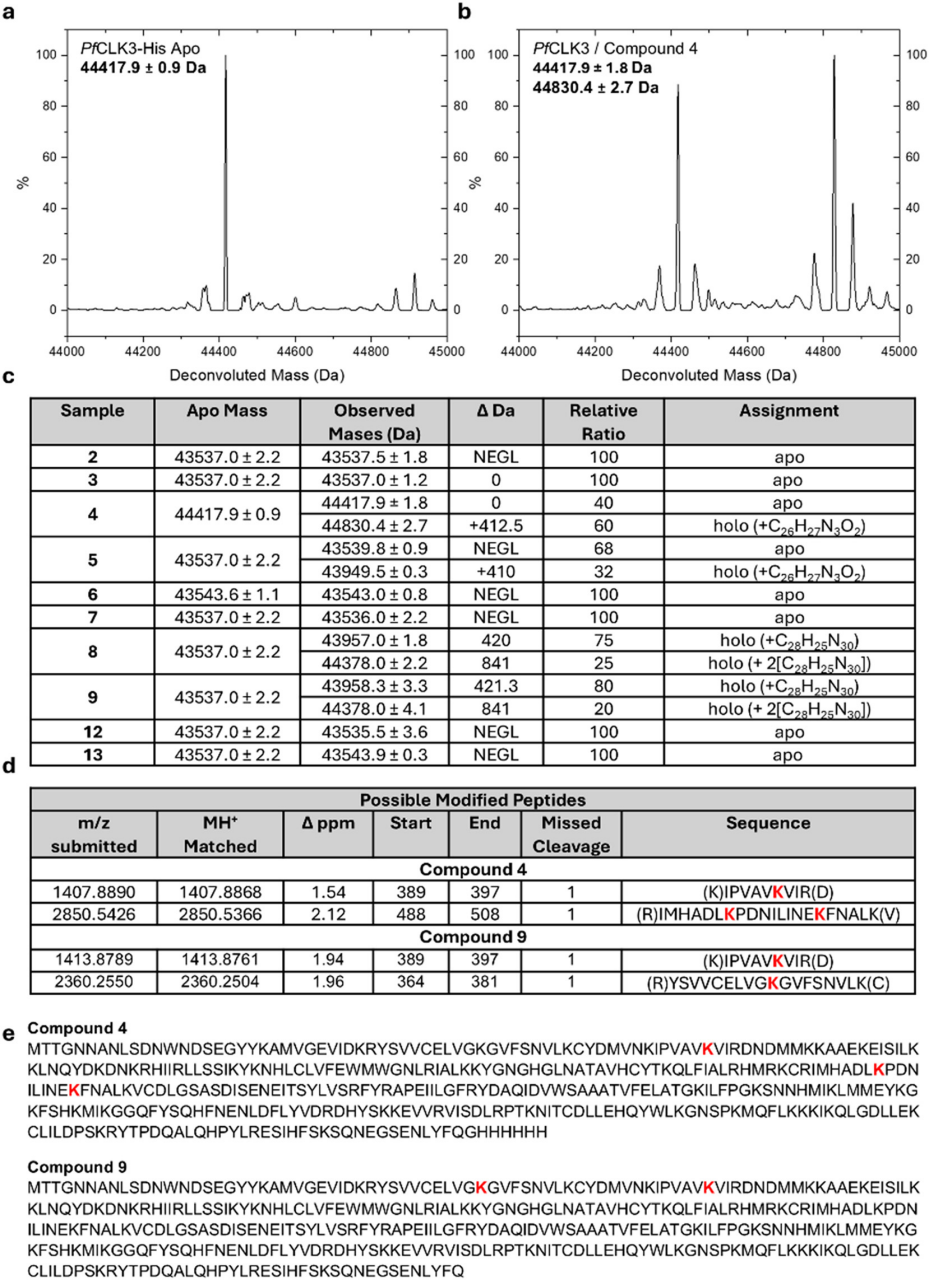
Protein mass spectrometry of compounds 2–13 incubated with *Pf*CLK3. a, *Pf*CLK3 apo spectrum b, *Pf*CLK3-compound 4 adduct spectrum. c, Intact mass spectrometry results of compounds 2–9, 12 and 13. For compound 4, *Pf*CLK3 with a C-terminal His-tag was used (sequence given in e). d, List of modified tryptic peptides observed after tryptic digest of compound 4 and 9 adducts. e, Sequences of *Pf*CLK3 constructs used. Modified lysines shown in red.

All compounds were then evaluated for covalent binding with *Pf*CLK3 kinase domain (amino acid residues 334–699) using intact protein LC-MS (Fig. 3). Compounds were incubated at pH 7.4 in a 1 : 5 protein to compound ratio and analysed for covalent adduct formation using ESI TOF. While salicylic aldehydes 4 and 5 and ethynyl benzaldehydes 8 and 9 showed covalent adduct formation, all others yielded the mass of apo protein only. Compounds 4 and 5 showed partial covalent bond formation (32–60% modification) consistent with reversible covalent adduct formation.^24^ These modified species had a mass consistent with imine formation. Compounds 8 and 9 yielded a fully modified species at 43 957 Da, which can be attributed to the mass of the apo protein + the mass of the pyridinium product adduct. A minor mass (20–25%) of 44 378 Da was also detected, consistent with a doubly modified *Pf*CLK3 kinase domain. This implies that cross reactivity with non-catalytic lysine residues is possible using the ethynyl benzaldehyde functionality, although a minor product.

**Fig. 4:**
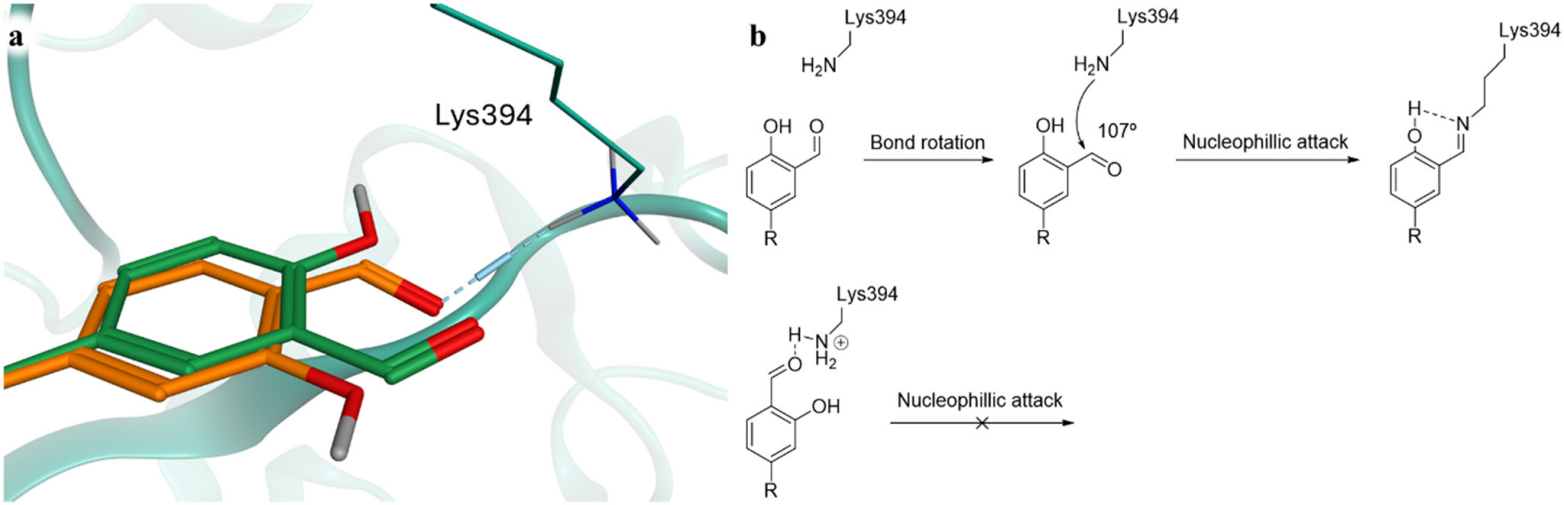
a, Lysine interacting moiety of compounds 4 and 6, docked into *Pf*CLK3 (PDB: 8RPC) and overlayed. b, Proposed mechanism of 4's covalent reaction with Lys394, and the inability of 6 to react.^24^

*Pf*CLK3 pretreated with either compound 4 or 9 was then digested with trypsin, and the resulting tryptic peptides analysed by tandem mass spectrometry (Fig. 4d and e). This subset of compounds sampled azaindole regioisomers as well as both aldehyde warheads used. For salicylic aldehyde 4, spectrometry analysis determined that Lys394 was the predominant site of modification, and this was confirmed by tandem MS. However, possible modification of a peptide containing Lys494 and Lys503 was also observed. This is a curious result, as a secondary adduct was not observed in the intact spectrum. Lys503 is distal from the ATP binding site, whereas Lys494 lies at its entrance. Adduct formation may be occurring with Lys494 as 4 approaches the binding-site.

For the tryptic digest of the *Pf*CLK3-9 adduct, a pyridinium modification was observed in peptides containing both the target Lys394, and Lys373. This is consistent with the intact mass spectrum, which detected a doubly modified species in ∼20% abundance *versus* the singly modified protein. Similar to the results of 4, Lys373 also lies at the entrance of the ATP binding site, and may be forming an adduct as the ethynyl benzaldehyde warhead enters the catalytic pocket. Overall, aldehydes 4 and 9 were successful in covalently binding *Pf*CLK3 with relatively good selectivity for Lys394.

An interesting observation was that only those compounds functionalised with an aldehyde at the *meta*-position (compounds 4, 5, 8 and 9) formed a covalent adduct with *Pf*CLK3. Those featuring a *para*-substituted aldehyde (compounds 6 and 7) were unable to covalently bind the protein. Rationalising this experimental data, we theorise that compounds 6 and 7 are unable to be attacked by Lys394 from the necessary Bürgi–Dunitz angle for nucleophilic attack of a carbonyl, and therefore unable to form the imine (Fig. 4c). Looking at compounds 4 and 6 docked reversibly into *Pf*CLK3 (Fig. 4A), compound 6 (orange) is predicted to form a hydrogen bond with Lys394, whereas compound 4 (green) does not, appearing to be too far away. This is consistent with experimental data from our previous work, which showed that a regioisomer of TCMDC-135051, where the carboxylic acid and isopropyl group were swapped, was inactive, implying a *meta*-substituted carbonyl is unable to interact with Lys394.^11^ Were compound 6 to form this hydrogen bond, this would restrict rotation of the C–C benzaldehyde bond, preventing the presentation of the carbonyl carbon towards Lys394 at a 107° angle. Compound 4 would have no such restriction, and could thus form the imine. This logic may equally apply to compounds 5, 7, 8 and 9.

### Kinase activity

**Fig. 5:**
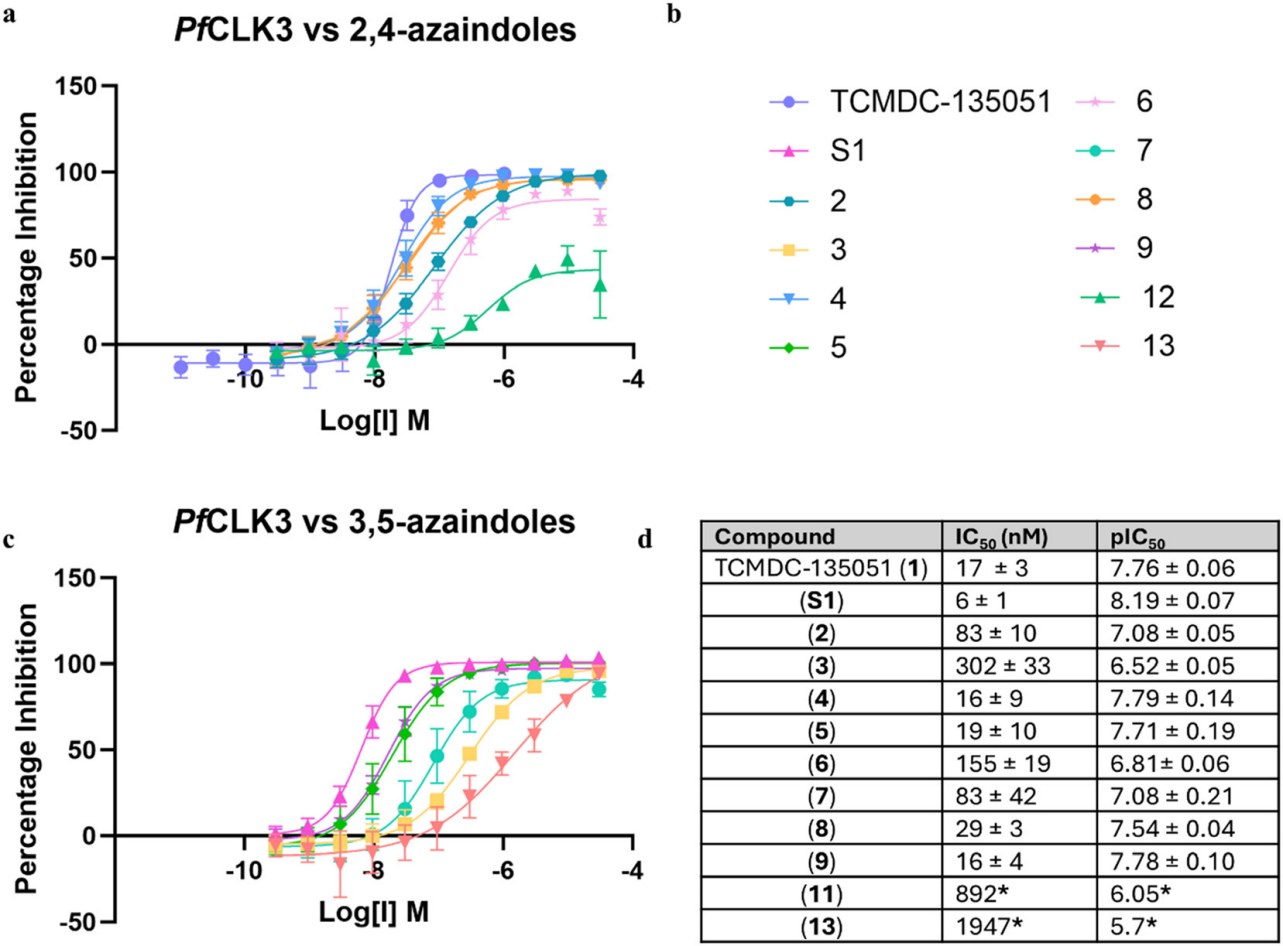
Potency of compounds 1–9 against recombinant *Pf*CLK3. a, Concentration response curve for the 2,4-substituted azaindoles (2, 4, 6 and 8) b, graph legend. c, Concentration response curve for the 3,5-substituted azaindoles (3, 5, 7 and 9). d, Table of apparent IC_50_ values all tested compounds. Errors given as standard deviations, *N* ≥ 3. *Concentration response curve not complete.

All compounds were then evaluated against recombinant *Pf*CLK3 using a TR-FRET kinase assay (Fig. 5). As a reversible control for the 3,5-substituted azaindoles, a regioisomer of TCMDC-135051 was used (compound S1, see ESI†). Consistent with the mass spectrometry, compounds 2, 4, 5, 8 and 9, which demonstrated covalent adduct, showed comparable potency to TCMDC-135051 (1). pIC_50_ values ranged from 7.12–7.92. This indicated that covalent adduct formation may be forming within the 2 hour incubation period of the kinase assay. Compounds 3, 6 and 7 which had no detectable adduct formation were less potent, with pIC_50_ values of 6.48–6.87. This is to be expected as previous work established that the benzoic acid-Lys394 salt bridge of TCMDC-135051 is crucial for kinase activity. Disruption of this ionic interaction, with the absence of covalent adduct formation, is therefore not well tolerated. The establishment of a covalent bond, on the other hand, maintains low nanomolar potency. Acrylamide 11 was poorly active, not reaching 100% inhibition at the highest concentration tested, whereas acrylamide 13 showed significantly lower activity than all other analogues.

Compounds which formed covalent bonds with *Pf*CLK3 were then tested in the same assay using an increased concentration of ATP (3 mM) rather than the Km for *Pf*CLK3 (5 μM). This was designed to emulate physiological concentrations of ATP in the parasite. Our previous work has shown this to be predictive of parasiticidal activity.^12^ Kinase activity at 3 mM ATP was obtained for covalent compounds 4, 5, 8 and 9. All compounds lost significant potency in these assays (Fig. S1†). This is hypothesised to be explained by the disruption of the key carboxylic acid-Lys394 reversible interaction of TCMDC-135051 (1). Covalent binding is a two-step process, a reversible binding event (measured by the dissociation constant *k*_off_/*k*_on_) followed by nucleophilic attack (measured by the rate constant *k*_inact_) once the electrophile is placed in proximity to the nucleophile.^18^ It is therefore hypothesised that at physiological ATP concentrations, *k*_on_ for 4, 5, 8 and 9 is reduced due to the absence of the carboxylic acid-Lys394 salt bridge, and the covalent reaction cannot occur.

### Parasiticidal activity

**Fig. 6:**
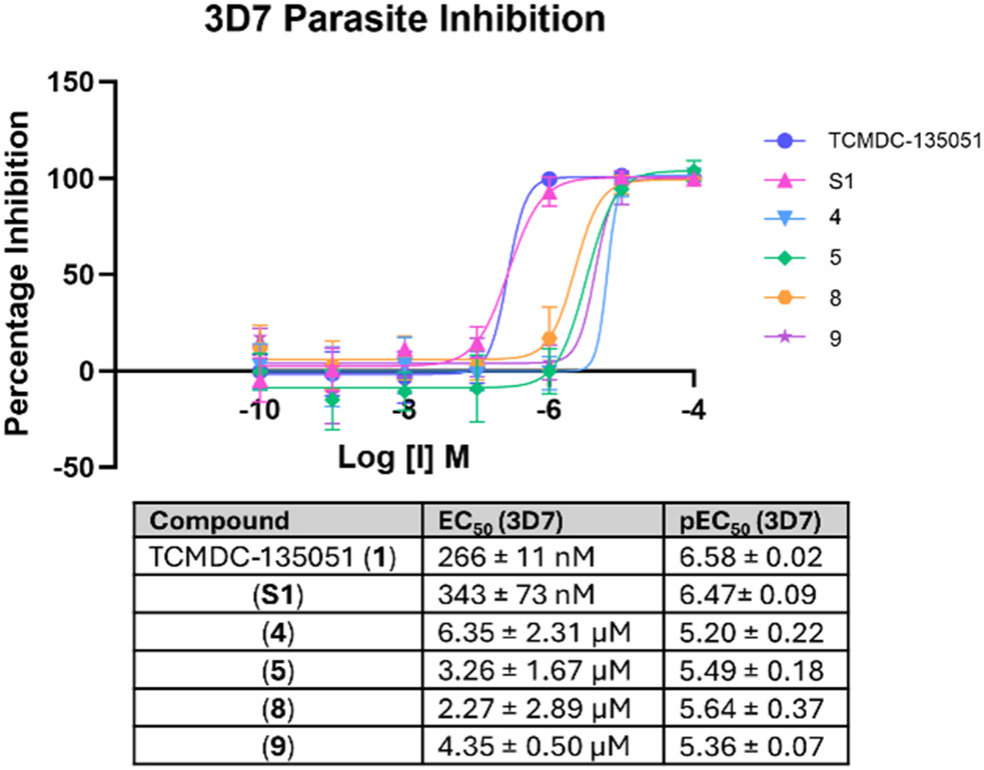
Parasiticidal potency of covalent compounds against *Pf*3D7 parasites. Errors given as standard deviations, *N* ≥ 3.

Compounds 4, 5, 8 and 9 were then assessed against 3D7 *Plasmodium falciparum* parasites (Fig. 6). This is a well-characterised and widely used cell line within the malaria community, providing a good metric with which to benchmark our inhibitors against others in the field.^35^ This strain has also shown resistance to anti-malarial drugs such as dihydroorotate dehydrogenase (DHODH) inhibitors and artemisinin, which this study aims to avoid.^36^ While the covalent compounds displayed lower potency than their reversible parent compounds, all were still active at micromolar concentrations, with pEC_50_ values of 5.20–5.64. This reduction in activity could be explained by elevated ATP levels in the parasite. The *in vitro* kinase assay was performed at Km ATP (log[ATP] = 5.30), while in the parasite log[ATP] = 2.52 is typical.^37^ Disrupting the benzoic acid-Lys394 interaction of TCMDC-135051 has clearly decreased the compound's affinity to the kinase, decreasing *k*_on_ for *Pf*CLK3 at high ATP concentrations, as predicted in the [ATP] = 3 mM assays. Optimisation of the ligand's *k*_off_/*k*_on_ should therefore provide lysine targeting covalent inhibitors with enhanced parasiticidal activity.

### Selectivity

**Fig. 7:**
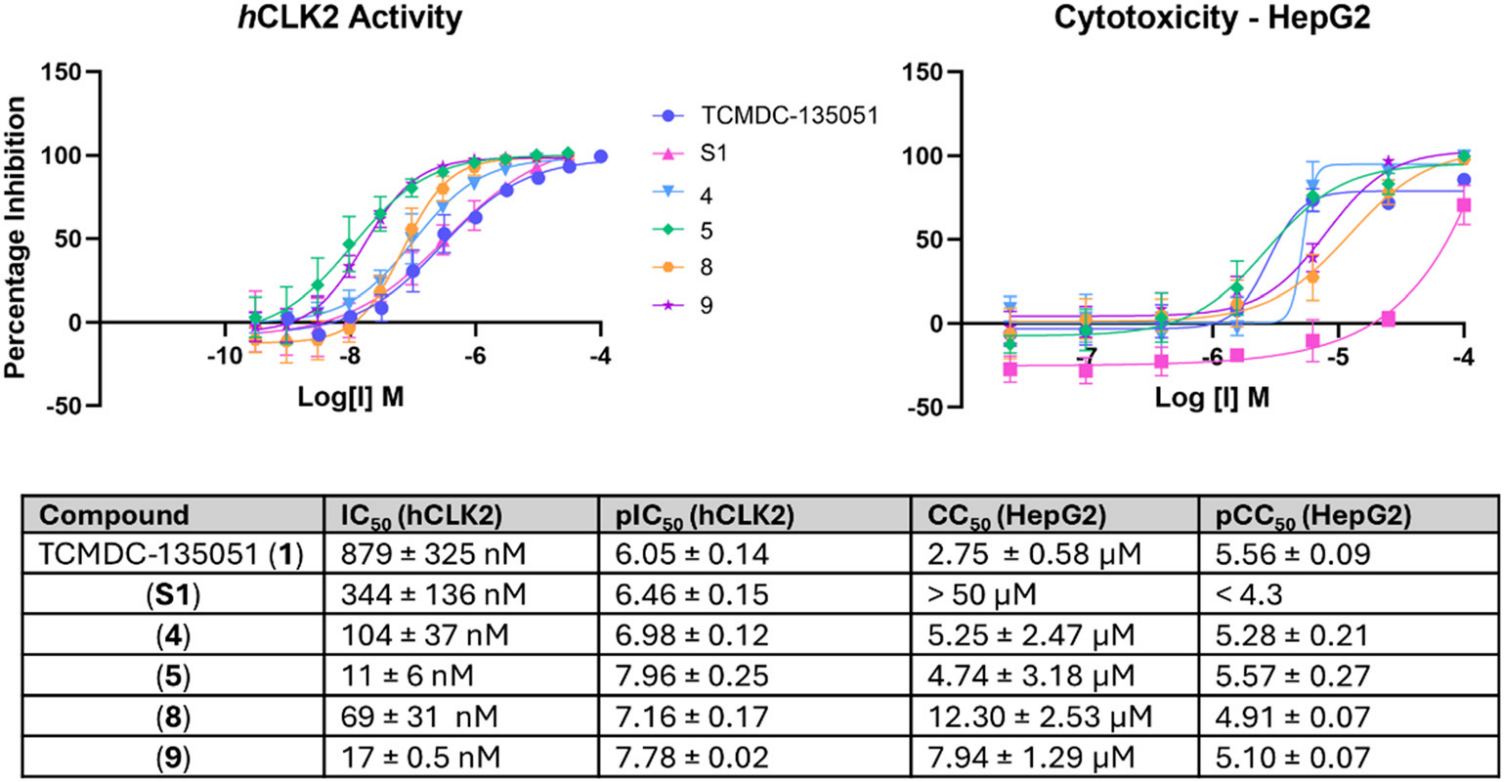
Selectivity of compounds 1, S1, 4, 5, 8 and 9 against hCLK2 and cytotoxicity against HepG2 cells. Errors given as standard deviations, *N* ≥ 3.

Selectivity is an important factor for all drug discovery campaigns, but particularly for kinase inhibitors, as protein kinases have a well conserved ATP binding site. Given this work aims to target a conserved residue, selectivity must be derived from the reversible ligand rather than the covalent interaction. As a result, a selectivity profile similar to that of TCMDC-135051 (1) would be expected. Selectivity for compounds 4, 5, 8 and 9 was evaluated against human (h)CLK2, the closest human kinase sequence to *Pf*CLK3 (Fig. 7). This set was expected to demonstrate a similar selectivity profile to TCMDC-135051 and S1 as the ligands remain constant and the catalytic lysine is conserved in both kinase enzymes. Interestingly, all covalent compounds were found to be significantly more potent against hCLK2. The 3,5-substituted azaindoles were more potent against hCLK2 than the 2,4-substituted series, implying this scaffold may be favourable for inhibition of this human kinase. In particular, compounds 5 and 9 were 20 and 31-fold more potent respectively than their parent S1. This implies that 3,5-substituted azaindoles with aldehyde-based warheads may be suitable for targeting hCLK2's catalytic lysine, which has not previously been reported in the literature.^19,38^

Cytotoxicity was assessed in HepG2 cells to complement the selectivity evaluations (Fig. 7). All compounds showed comparable cytotoxicity (pCC_50_ values of 4.90–5.57) to that of the parent compound TCMDC-135051 (5.56). This could suggest a similar kinome and proteome-wide selectivity to that of TCMDC-135051. This is gratifying in the development of covalent inhibitors, implying no additional toxic off-target effects are observed. Enhancing selectivity further requires improvement of the reversible interactions of the scaffold with *Pf*CLK3 relative to off-targets. A significant drug discovery effort is currently underway to achieve this.

## Conclusion

In this work, covalent inhibitors of *Pf*CLK3 targeting the catalytic Lysine-394 have been developed. Inhibitors were designed using structure-based drug design, guided by our previously published co-crystal structure of *Pf*CLK3 kinase domain with TCMDC-135051 (1).^12^ Ultimately, four out of ten designed compounds demonstrated covalent adduct formation, validating Lys394's susceptibility to this inhibition strategy. These analogues also maintained the potency of the hit compound when screened against recombinant *Pf*CLK3. Given the critical role of the benzoic acid for TCMDC-135051's activity, a substantial modification to a covalent warhead was well tolerated. Selectivity was also largely maintained, as would be expected from using the same reversible scaffold to target a conserved residue. While compounds 5, 8 and 9 were less selective for *Pf*CLK3's over closest human analogue hCLK2 than their reversible parent compounds, this may open an avenue for the development of benzaldehyde-based inhibitors targeting Lys193 of this human kinase. Compounds were well tolerated in a cytotoxicity screen against HepG2 cells, implying a good level of kinome and proteome-wide selectivity despite potent hCLK2 inhibition.

From the parasiticidal potency of these compounds, it is clear that the benzoic acid-Lys394 interaction is key for reversible binding of TCMDC-135051 (1), and the covalent molecules disrupt this. As covalent binding is a two-step process, involving reversible binding (*k*_i_) followed by a covalent binding event (*k*_inact_), the *k*_i_ of these compounds at higher ATP concentrations must be optimised to facilitate *k*_inact_ and therefore overall potency. By improving the reversible binding of the scaffold such that the potency is less reliant on the ionic interaction with Lys394, covalent bod formation should be facilitated in parasite. This work presents proof-of-concept molecules that covalently bind to and inhibit *Pf*CLK3 at [ATP] = *K*_m_ concentrations.

This strategy may be of use against other malarial kinases, all of which harbour catalytic lysine residues. MMV390048 for instance, which reached phase II clinical trials, features a methylsulfonylphenyl group which is believed to interact with the catalytic Lys1308 of *Pf*PI4K.^39^ Substitution with a sulfonyl fluoride or an aldehyde-based warhead, as in this current work, may prove an effective strategy. Covalently engaging the catalytic residue may evade future resistance to clinical candidate MMV390048, which showed a high propensity for resistance in drug pressure studies.^40,41^

In conclusion, this study validates the strategy of covalent inhibition of *Pf*CLK3 by targeting catalytic Lys394, demonstrating its potential as a novel approach for malaria drug discovery. By leveraging the appropriate scaffold, this inhibitor class could enable the development of new therapeutics against this essential kinase. Furthermore, this covalent inhibition strategy may extend to other malarial kinases that possess catalytic lysine residues. Engaging the catalytic lysine covalently may also reduce the likelihood of resistance emerging. Our work represents the first reported lysine-targeting covalent inhibitor for malaria, laying the foundation for future advancements in this field.

## Author contributions

S. B. B. and A. G. J. conceived of the study. S. B. B. and A. M. synthesised compounds. S. B. B. and D. J. C. performed protein mass spectrometry experiments. G. C., A. B. and S. S. carried out biochemical kinase assays, and cytotoxicity screening against HepG2. S. S. and L. V. C. performed parasite assays. A. G. J., A. B. T. and G. M. acquired funding to carry out the project. S. B. B. wrote the manuscript, and all authors contributed to manuscript editing.

## Conflicts of interest

A. G. J. and S. B. B. are inventors on a provisional patent (008521262) filed by the University of Glasgow on covalent anti-malarial inhibitors and their analogs that target *Pf*CLK3. A. B. T., A. G. J. & G. M. are shareholders of and employees of Keltic Pharma Therapeutics Ltd. The other authors declare no competing interests.

## Supplementary Material

MD-OLF-D5MD00335K-s001

MD-OLF-D5MD00335K-s002

MD-OLF-D5MD00335K-s003

MD-OLF-D5MD00335K-s004

## Data Availability

The ESI† includes detailed experimental procedures, compound characterisation data, biochemical and parasiticidal activity data, as well as copies of ^1^H and ^13^C NMR spectra and analytical HPLC traces for all final compounds (PDF). All molecular docking poses are provided as PDB files, and tryptic peptide masses are provided also.
