# Supplementary material for: Lysine targeting covalent inhibitors of malarial kinase PfCLK3
Source: RSC Med Chem. 2025 May 27;16(8):3530–40. doi: 10.1039/d5md00335k (PMC12164071; doi:10.1039/d5md00335k)
Supplement: MD-016-D5MD00335K-s003 [file MD-016-D5MD00335K-s003.pdf]

Supplementary Information (SI) for RSC Medicinal Chemistry.
This journal is © The Royal Society of Chemistry 2025

Lysine targeting covalent inhibitors of malarial kinase *Pf*CLK3

Skye B. Brettell,† Gillian Cann,§ Abbey Begen,§ Saumya Sharma,§ Amit Mahindra,† Lauren Carruthers§, Graeme Milligan,‡ David J Clarke,¥ Andrew B. Tobin,‡ and Andrew G. Jamieson†*

†School of Chemistry, The Advanced Research Centre, University of Glasgow, 11 Chapel Lane, G11 6EW, U.K. ‡Centre for Translational Pharmacology, The Advanced Research Centre, University of Glasgow, 11 Chapel Lane, G11 6EW, U.K. §KelticPharma Therapeutics, The Advanced Research Centre, University of Glasgow, 11 Chapel Lane, G11 6EW, U.K. ¥EaSTCHEM School of Chemistry, University of Edinburgh, Joseph Black Building, David, Brewster Road, Edinburgh, EH9 3FJ, U.K. *andrew.jamieson.2@glasgow.ac.uk

Contents

Molecular Docking 2

Chemical Synthesis and Characterisation Data 3

Protein purification 19

Protein Mass Spectrometry 19

Time Resolved Förster Resonance Energy Transfer (TR-FRET) Assay 20

***P. falciparum* (3D7) culture and synchronisation.** 20

Ex vivo *P. Falciparum* (3D7) inhibition assay. 21

Human cell viability assay. 21

***Pf*CLK3 Activity at Elevated ATP Concentrations** 22

NMR Spectra for Novel Compounds 22

HPLC data for all tested compounds 39

Molecular Docking

All molecular docking was performed using MOE 2020.0901, using their in-house Amber10:EHT force field. Crystal structure 8RPC prepared using the “Quickprep” function in MOE. The ATP binding site was defined by “Compute” > “Sitefinder” > “Apply”. “Dummy atoms” were then created to characterize the binding site.

All ligands were drawn in ChemDraw and their 3D structure was generated by “Compute” > “Prepare” > “Quickprep”. Prepared ligands were saved to the working directory as a new database. Docking was carried out using the “Induced fit” function.

For covalent docking of cysteine-reactive molecules, one “dummy atom” created using the Site Finder tool was moved to sit adjacent to Cys368, and dummies were used to define the binding site. The reactive site was set to “selected atoms” and the thiol of Cys368 was selected in the visualiser. The beta-mercapto carbonyl 1,4-addition reaction was selected, and “Rigid Receptor” refinement was used. For covalent docking of lysine reactive molecules, the reactive site was set to “selected atoms” and the amine of Lys394 was selected in the visualizer. For the covalent reaction, an appropriate .rxn file created in ChemDraw and selected in MOE. The “Induced fit” refinement was used for lysine reactive compounds.

After solutions were generated, the “Complex” field from the results database was then copied into MOE for each ligand. The ligands “Tag” was changed to that of the receptor in the System Manager, and the complex was minimised using the “Quick Prep” function, with “Structure Preparation” and “Protonate3D” options deselected. This minimised covalent complexes which could then be analysed using the S score, E_conf, binding pose and observed clash.

Chemical Synthesis and Characterisation Data

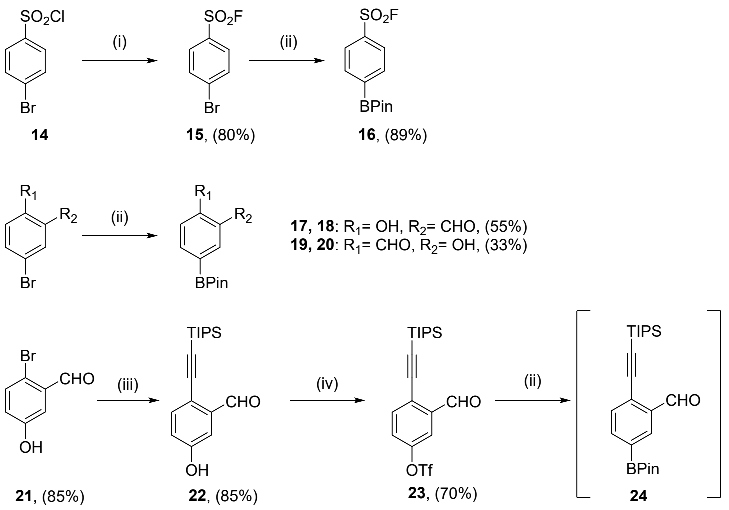

4-Bromobenzenesulfonyl fluoride (15)

A round bottom flask was charged with KHF_2 (70 mg, 6.4 mmol, 2.3 equiv.) in H_2O (1.4 mL, 4.6 M), and 4-bromobenzenesulfonyl chloride (700 mg, 2.8 mmol, 1 equiv.) in ACN (2.8 mL, 1M) and stirred at room temperature for 24 hours. The reaction was then partitioned between water and EtOAc. The organic layer was dried over magnesium sulfate and concentrated *in vacuo* to give compound **15** (527 mg, 80% yield); **R_f**: 0.23 (100% petroleum ether); **^{1}H NMR** (400 MHz, $CDCl_3$) δ 7.88 (d, J = 8.8 Hz, 2H), 7.79 (d, J = 8.8 Hz, 2H); **^{13}C NMR** (101 MHz, $CDCl_3$) δ 133.3, 132.1 (d, $^2J_{CF}$ = 25.6), 131.5, 130.0; **^{19}F NMR** (376 MHz, $CDCl_3$) δ 66.4. All characterisation data was in accordance with reported literature values.[1]

4-(4,4,5,5-Tetramethyl-1,3,2-dioxaborolan-2-yl)benzenesulfonyl fluoride (16)

To a solution of compound **15** (527 mg, 2.2 mmol, 1 equiv.) in degassed anhydrous dioxane was added bis(pinacolato)diboron (843 mg, 3.3 mmol, 1.5 equiv.), potassium acetate (650 mg, 6.6 mmol, 3 equiv.) and 1,1 -Bis(diphenylphosphino)ferrocene dichloropalladium, complex (180 mg, 0.22 mmol, 10 mol%) under inert atmosphere. The reaction was heated at 80°C overnight before being filtered through a bed of celite, eluted with methanol and concentrated *in vacuo*. The residue was partitioned between ethyl acetate and water, and the organic layer dried over magnesium sulfate. The crude mixture was purified by automatic column chromatography (10-100% EtOAc in petroleum ether) to afford compound **16** as an off-white solid (562 mg, 89% yield); **R_f**: 0.26 (10% EtOAc in petroleum ether); **^{1}H NMR** (400 MHz, $CDCl_3$) δ 8.03 (d, J = 8.0 Hz, 2H), 7.98 (d, J = 8.0 Hz, 2H), 1.36 (s, 12H); **^{13}C**

NMR (101 MHz, $CDCl_3$) δ 135.8, 135.2 (d, $^2J_{CF}$ = 23.9 Hz), 127.4, 84.9, 25.0 (1C missing); **^{19}F NMR** (376 MHz, $CDCl_3$) δ 65.7; **^{11}B NMR** (128 MHz, $CDCl_3$) δ 21.4. All characterisation data was in accordance with reported literature values.[1]

2-hydroxy-4-(4,4,5,5-tetramethyl-1,3,2-dioxaborolan-2-yl)benzaldehyde (20)

To a solution of commercially available 4-bromosalicyclic aldehyde (100 mg, 0.50 mmol, 1 equiv.) in degassed anhydrous 1,4-dioxane (2.7 mL, 0.19 M) was added bis(pinacolato)diboron (189 mg, 0.75 mmol, 1.5 equiv.), potassium acetate (146 mg, 1.50 mmol, 3 equiv.) and 1,1 -Bis(diphenylphosphino)ferrocene dichloropalladium, complex (40 mg, 0.05 mmol, 10 mol%) under inert atmosphere. The reaction was heated at 80 °C overnight before being filtered through a bed of celite, eluted with methanol and concentrated *in vacuo*. The residue was partitioned between ethyl acetate and water, and the organic layer dried over magnesium sulfate. The crude mixture was purified by automatic column chromatography (10-50% EtOAc in petroleum ether) to afford compound **20** as a white solid (40 mg, 33% yield); **R_f**: 0.7 (50% EtOAc in petroleum ether); **^{1}H NMR** (400 MHz, $CDCl_3$) δ 10.82 (s, 1H,), 9.92 (s, 1H), 7.54 (d, J= 8.1 Hz, 1H), 7.44 – 7.40 (m, 2H), 1.35 (s, 12H); **^{13}C NMR (101 MHz, $CDCl_3$)** δ 196.8, 160.6, 132.6, 125.4, 123.8, 122.0, 84.4, 24.8; **HRMS** m/z calcd for $C_{13}H_{18}BO_4$ [M+H]+ calc for 249.1295 found 249.1285. All characterisation data was in accordance with reported literature values.[2]

2-Hydroxy-5-(4,4,5,5-tetramethyl-1,3,2-dioxaborolan-2-yl)benzaldehyde (18)

To a solution of commercially available 3-bromosalicyclic aldehyde (2.0 g, 10.0 mmol, 1 equiv.) in degassed anhydrous 1,4-dioxane (30 mL, 0.33 M) was added bis(pinacolato)diboron (3.8 g, 15.0 mmol, 1.5 equiv..), potassium acetate (2.9 g, 30.0 mmol, 3 equiv.) and 1,1 -Bis(diphenylphosphino)ferrocene dichloropalladium, complex (728 mg, 1.0 mmol, 10 mol%) under inert atmosphere. The reaction was heated at 80 °C overnight, cooled to room temperature and concentrated *in vacuo* before being partitioned between EtOAc (30 mL) and water (30 mL). The aqueous layer was washed with EtOAc, and the combined organic layers dried over magnesium sulfate. The crude mixture was purified by automatic column chromatography to give compound **18** as a white solid (1.35 g, 55% yield); **R_f** 0.3 (10% EtOAc in Petroleum ether); **^{1}H NMR (400 MHz, $CDCl_3$)** δ 11.22 (t, J = 1.1 Hz, 1H), 9.92 (t, J = 1.5 Hz, 1H), 8.04 (t, J = 1.5 Hz, 1H), 7.94 (dt, J = 8.4, 1.5 Hz, 1H), 6.97 (dt, J = 8.4, 1.5 Hz, 1H), 1.35 (m, 12H); **^{13}C NMR (101 MHz, $CDCl_3$)** δ 196.9, 164.0, 143.2, 141.4,

120.4, 117.1, 84.1, 24.9. All characterisation data was in accordance with reported literature values.[2]

5-hydroxy-2-((triisopropylsilyl)ethynyl)benzaldehyde (22)

To a solution of commercially available 5-hydroxy-2-bromo-benzaldehyde(200 mg, 0.1 mmol, 1 equiv.) in degassed triethylamine (6 mL, 0.17 M) was added Bis(triphenylphosphine)palladium(II) dichloride (35 mg, 0.005 mmol, 5 mol%), copper iodide (6 mg, 0.003 mmol, 5 mol%) and triisopropylsilylacetylene (0.67 mL, 3.0 mmol, 3 equiv.). The resulting mixture was heated at 50 °C for 3 hours, then was cooled to room temperature and concentrated *in vacuo.* This residue was then partitioned between EtOAc (10 mL) and water (10 mL). The organic layer was then washed with brine (10 mL) and then dried over sodium sulfate, filtered and concentrated *in vacuo*. The resulting residue was then purified by automated column chromatography, eluting in 10-50% EtOAc in petroleum ether to give **22** as a white solid (256 mg, 85% yield); **R_f** 0.38 (30% EtOAc in petroleum ether) **^{1}H NMR (400 MHz, $CDCl_3$)** δ 10.52 (s, 1H), 7.76 (br s, 1H), 7.49 (d, *J* = 8.5 Hz, 1H), 7.49 (d, *J* = 2.8 Hz, 1H), 7.12 (dd, *J* = 8.5, 2.8 Hz, 1H), 1.21 – 1.04 (m, 21H); **^{13}C NMR (101 MHz, $CDCl_3$)** δ 192.3, 156.4, 137.5, 135.7, 121.9, 119.9, 112.7, 101.9, 97.1, 18.7, 11.3. All other characterisation data was in accordance with reported literature values.[3]

5-formyl-4-((triisopropylsilyl)ethynyl)phenyl trifluoromethanesulfonate (23)

To a solution of **22** (237 mg, 0.78 mmol, 1 equiv.) and triethylamine (0.213 mL, 1.57 mmol, 2 equiv.) in anhydrous DCM (2.6 mL, 0.3 M) at 0°C was added dropwise Trifluoromethanesulfonyl chloride (83 µL, 0.78 mmol, 1 equiv.). A pale precipitate formed. The solution was allowed to warm to room temperature and stirred for 2 hours. Water (10 mL) was added, and the aqueous layer was washed with DCM (3 x 10 mL). The combined organic layers were dried over sodium sulfate, filtered and concentrated *in vacuo*. The resulting residue was purified by automated column chromatography, eluting with 0-30% EtOAc in petroleum ether to give compound **23** (237 mg, 70% yield); **R_f** 0.48 (10% EtOAc in petroleum ether); **^{1}H NMR (400 MHz, $CDCl_3$)** δ 10.56 (s, 1H), 7.79 (d, *J* = 2.7 Hz, 1H), 7.69 (d, *J* = 8.6 Hz, 1H), 7.46 (dd, *J* = 8.6, 2.7 Hz, 1H), 1.17 – 1.11 (m, 21H); **^{13}C NMR (101 MHz, $CDCl_3$)** δ 189.7, 149.3, 138.2, 136.1, 127.2, 126.7, 119.8, 118.8 ($^4J_{CF}$, *J* = 362. 6 Hz), 102.2, 100.2, 18.8, 11.3; **^{19}F NMR (377 MHz, $CDCl_3$)** δ -72.70; **HRMS** Mass ion not found.

5-(4,4,5,5-tetramethyl-1,3,2-dioxaborolan-2-yl)-2-{2-[tris(propan-2-yl)silyl]ethynyl}benzaldehyde (24)

To a solution of compound **23** (100 mg, 0.23 mmol, 1 equiv.) in anhydrous dioxane (2.3 mL, 0.1 M) was added bis(pinacolato)diboron (70 mg, 0.28 mmol, 1.2 equiv.), and potassium acetate (68 mg, 0.69 mmol, 3 equiv.). The solution was degassed with nitrogen for 5 minutes before Bis(diphenylphosphino)ferrocene dichloropalladium complex (19 mg, 0.02 mmol, 10 mol%) was added. The reaction was heated to reflux for 4 hours, before being cooled to room temperature and partitioned between EtOAc (10 mL) and water (10 mL). The aqueous layer was washed with EtOAc (3 x 10 Ml) and the organic layers were dried over sodium sulfate, filtered and concentrated *in vacuo.* The resulting residue proved unstable to column chromatography, and was therefore used in the next step without further purification.

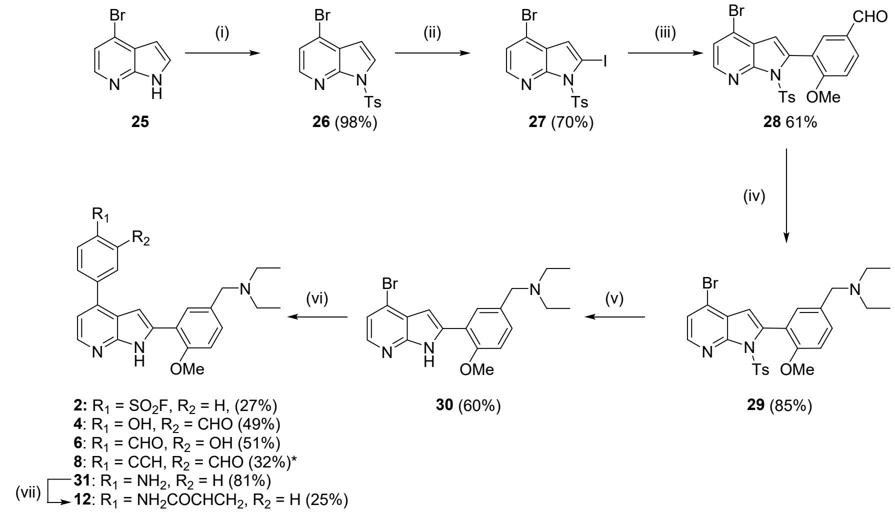

4-Bromo-1-tosyl-1H-pyrrolo[2,3-b]pyridine (26)

To a stirring solution of 4-bromo-1H-pyrrolo[2,3-b]pyridine (2.5 g, 12.7 mmol, 1.0 eq) in anhydrous dichloromethane (40 mL) cooled in an ice-water bath to 0 °C was added sodium hydride (60% in mineral oil, 1.5 g, 38.1 mmol, 3.0 eq) and the mixture was stirred under nitrogen for 15 min. Toluene sulphonyl chloride (7.3 g, 38.1 mmol, 3.0 eq) was added and the mixture was left to warm to rt whilst stirring under nitrogen for 18 h. The reaction mixture was slowly quenched with water and diluted with 1:1 water:DCM and two layers were separated. The aqueous layer was extracted with DCM and the combined organic layers were dried over magnesium sulphate and concentrated in vacuo to give a brown solid. This was purified using automated flash column chromatography eluting with 0-60% ethyl acetate:petroleum ether. The desired fractions were combined and concentrated in vacuo to give compound **26** as a

white solid (4.4 g, 98%). **R$_f$**: 0.55 (20% EtOAc in petroleum ether); **^{1}H NMR** (400 MHz, $CDCl_3$) δ 8.22 (d, *J* = 5.2 Hz, 1H), 8.06 (d, *J* = 8.4 Hz, 2H), 7.78 (d, *J* = 4.0 Hz, 1H), 7.35 (d, *J* = 5.3 Hz, 1H), 7.28 (d, *J* = 8.0 Hz, 2H), 6.64 (d, *J* = 4.0 Hz, 1H), 2.37 (s, 3H); **^{13}C NMR** (101 MHz, $CDCl_3$) δ: 146.8, 145.5, 145.0, 135.1, 129.7, 128.2, 127.0, 125.7, 124.4, 122.1, 104.9, 21.7; **HRMS** *m/z* calcd for $C_{14}H_{12}BrN_2O_2S$ $[M+H]^+$ 350.9797 found 350.9796. All characterisation was in accordance with that of the literature.[4]

4-Bromo-2-iodo-1-tosyl-1H-pyrrolo[2,3-b]pyridine (27)

To a two necked flask containing **26** (2.4 g, 6.8 mmol, 1.0 equiv..) in THF (80 mL) stirring at -78˚C under argon atmosphere was added Lithium diisopropylamide (2 M solution in THF, 4.6 mL, 8.8 mmol, 1.3 equiv..). The resulting solution was then stirred at -78 ºC for 90 mins. Iodine (2.6 g, 9.9 mmol, 1.5 equiv..) was added in one portion, and the reaction mixture was stirred at -78 °C for 60 mins. The reaction was quenched with saturated ammonium chloride solution and the organic layer was washed with aqueous sodium thiosulphate and brine before drying over magnesium sulphate. The residue was then purified by column chromatography (20% ethyl acetate-hexane) to give **27** as a colourless solid (2.26 g, 70%); **R$_f$**: 0.5 (20% EtOAc in petroleum ether); **^{1}H NMR** (400 MHz, $CDCl_3$) δ 8.11 (d, *J* = 5.2 Hz, 1H), 8.01 (d, *J* = 8.5 Hz, 2H), 7.23 (d, *J* = 5.2 Hz, 1H), 7.22-7.19 (m, 2H), 6.96 (s, 1H), 2.30 (s, 3H); **^{13}C NMR** (101 MHz, $CDCl_3$) δ: 149.1, 145.7, 144.7, 135.4, 129.8, 128.3, 125.3, 123.6, 122.4, 119.4, 21.7; ; **HRMS** *m/z* calcd for $C_{14}H_{11}BrIN_2O_2S$ $[M+H]^+$ 477.8671 found 478.8742. All characterisation was in accordance with that of the literature.[4]

3-[4-Bromo-1-tosyl-1H-pyrrolo[2,3-b]pyridin-2-yl]-4-methoxy-benzaldehyde (28)

To a solution of **27** (2.2g, 4.7 mmol, 1.0 equiv..) and tetrakis(triphenylphosphine)palladium(0) (0.27 g, 0.23 mmol, 0.05 equiv..) in 1,4-dioxane was added 5-formyl-2-methoxyphenyl boronic acid (0.841 g, 4.7 mmol, 1.0 equiv..) under a nitrogen atmosphere. Aqueous sodium carbonate (2 M, 16.3 mL, 33.9 mmol, 7.0 equiv..) was then added and the reaction mixture left to stir at 110 ºC for 18 hrs. Solvent was removed under vacuum and the crude was dissolved in ethyl acetate, poured into water and extracted with ethyl acetate. The organic layer was washed with brine before drying over magnesium sulphate and purified by flash column chromatography (30% ethyl acetate-hexane) to afford **28** as a yellow foam (1.37 g, 61%); **R$_f$**: 0.58 (50% EtOAc in petroleum ether; **^{1}H NMR** (400 MHz, $CDCl_3$) 9.90 (s, 1H), 8.15 (d, *J* = 5.3 Hz, 1H), 7.95 (dd, *J* = 8.5, 2.1 Hz, 1H), 7.84 (d, *J*

= 2.1 Hz, 1H), 7.73 (d, *J* = 8.5 Hz, 2H), 7.28 (d, *J* = 5.3 Hz, 1H), 7.13 (d, *J* = 8.5 Hz, 2H), 7.04 (d, *J* = 8.5 Hz, 1H), 6.52 (s, 1H), 3.85 (s, 3H), 2.27 (s, 3H); **^{13}C NMR** (101 MHz, $CDCl_3$) δ: 190.4, 163.5, 148.7, 145.1, 144.8, 137.6, 135.8, 134.4, 131.4, 129.4, 129.4, 128.1, 125.1, 123.3, 123.1, 122.3, 110.6, 107.9, 56.1, 21.6 ; **HRMS** *m/z* calcd for $C_{22}H_{18}BrN_2O_4S$ $[M+H]^+$ 485.0165 found 485.0164. All characterisation was in accordance with that of the literature.[4]

<u>*N*-(3-(4-bromo-1-tosyl-1*H*-pyrrolo[2,3-*b*]pyridin-2-yl)-4-methoxybenzyl)-*N*-Ethylethanamine (29)</u>

To a reaction vessel containing **28** (190 mg, 0.39 mmol, 1 equiv..) in 1,2-dichloroethane (6 mL) was added diethylamine (0.12 ml, 1.17 mmol, 3.0 equiv..) and titanium isopropoxide (0.23 mL, 0.78 mmol, 2 equiv..) and left to stir for 5 minutes. Sodium triacetoxyborohydride (200 mg, 0.98 mmol, 2.5 equiv..) was then added as one portion and the reaction left to stir for 3 hours. Another portion of sodium triacetoxyborohydride (83 mg, 0.39 mmol, 1.0 equiv..) was added, and the reaction was left to stir for 18 h. The reaction was then quenched by the addition of ammonium hydroxide solution and extracted with dichloromethane. The organic layer was washed with water and dried over magnesium sulphate. The crude was then purified using column chromatography (0-15% MeOH in DCM) and afforded **29** as a brown oil (181 mg, 85 %); **^{1}H NMR (400 MHz, CD_3OD)** δ: 8.15 (d, *J* = 5.3 Hz, 1H), 7.76 (d, *J* = 8.4 Hz, 2H), 7.65 (dd, *J* = 8.5, 2.4 Hz, 1H), 7.62 (d, *J* = 2.3 Hz, 1H), 7.47 (d, *J* = 5.3 Hz, 1H), 7.31 (d, *J* = 8.0 Hz, 2H), 7.21 (d, *J* = 8.5 Hz, 1H), 6.65 (s, 1H), 4.45-4.35 (m, 2H), 3.81 (s, 3H), 3.31-3.23 (m, 4H), 2.36 (s, 3H), 1.38 (t, *J* = 7.3 Hz, 7H); **^{13}C NMR (101 MHz, CD_3OD)** δ: 159.3, 148.4, 145.7, 144.3, 138.2, 135.5, 133.7, 133.6, 129.1, 127.6, 124.7, 123.3, 122.3, 122.3, 120.8, 111.1, 107.6, 55.2, 54.9, 20.1; **HRMS** *m/z calcd* for $C_{26}H_{28}BrN_3O_3S$ [M+H]+ 541.1035 *found* 542.1093; All characterisation was in accordance with that of the literature.[4]

<u>[(3-{4-bromo-1H-pyrrolo[2,3-b]pyridin-2-yl}-4-methoxyphenyl)methyl]diethylamine (30)</u>

Compound **29** (1.14 g, 2.10 mmol, 1.0 equiv.) was dissolved in MeOH and treated with potassium carbonate (1g, 7.33 mmol, 3.5 equiv.) and heated to reflux for 18 hours. The solvent was then concentrated *in vacuo* and the resulting residue was dissolved in DCM and filtered. The filtrate was concentrated in vacuo to afford a yellow oil **30** (570 mg, 60%); **^{1}H NMR (400 MHz, CD_3OD)** δ: 8.00 (d, *J* = 5.3 Hz, 1H), 7.83 (d, *J* = 2.3 Hz, 1H), 7.36 (dd, *J* = 8.5, 2.2 Hz, 1H), 7.29 (d, *J* = 5.3 Hz, 1H), 7.13 (d, *J* = 8.5 Hz, 1H), 6.91 (s, 1H), 4.00 (s, 3H),

3.75 (s, 2H), 3.31 (p, *J* = 1.6 Hz, 1H), 2.71 (q, *J* = 7.2 Hz, 4H), 1.15 (t, *J* = 7.2 Hz, 6H); **^{13}C NMR (101 MHz, CD3OD)** δ: 156.3, 147.9, 142.1, 136.9, 131.2, 129.7, 124.11, 122.5, 119.4, 118.8, 111.6, 98.8, 56.1, 54.9, 9.4; **HRMS** *m/z calcd* for $C_{19}H_{22}BrN_3O$ [M+H]+ 388.1019 *found* 388.1022.

4-(2-{5-[(diethylamino)methyl]-2-methoxyphenyl}-1H-pyrrolo[2,3-b]pyridin-4-yl)benzene-1-sulfonyl fluoride (2)

To a solution of compound **30** (25 mg, 0.06 mmol, 1 equiv..) in degassed dioxane (0.6 mL, 0.07M) was added compound **16** (28 mg, 0.10 mmol, 1.5 equiv.), 1,1-Bis(diphenylphosphino)ferrocene dichloropalladium complex (2 mg, 0.003 mmol, 5 mol%) and K_3PO_4 (2M aq, 0.096 mL, 0.3 mmol, 3 equiv..). The solution was purged with nitrogen for 5 mins and then microwaved at 110 ºC for 1 h. The reaction was allowed to cool to room temperature and the mixture was evaporated and the resulting residue was purified using reverse phase flash chromatography an Isolera one with a 25g C18 column (10-70% ACN +0.1% acetic acid in H_2O + 0.1% acetic acid) to give compound **2** as a yellow solid (7.1 mg, 27% yield, 90% purity*); **^{1}H NMR (400 MHz, DMSO)** δ 12.08 (s, 1H), 8.37 (d, *J* = 5.0 Hz, 1H), 8.32 (d, *J* = 8.2 Hz, 2H), 8.21 (d, *J* = 8.2 Hz, 2H), 7.85 (s, 1H), 7.38 (d, *J* = 8.7 Hz, 1H), 7.32 (d, *J* = 5.0 Hz, 1H), 7.21 – 7.06 (m, 2H), 3.92 (s, 3H), 3.67 (br s, 2H), 2.61 (br s, 4H), 1.91 (acetic acid salt), 1.05 (m, 6H)*; **^{13}C NMR (101 MHz, DMSO)** δ 172.0, 155.8, 149.7, 146.4, 143.1, 137.1, 136.7, 130.0, 129.2, 127.6, 126.2, 125.5, 119.4, 117.9, 114.9, 111.9, 98.7, 55.7**, 45.9, 21.0; **^{19}F NMR (376 MHz, DMSO)** δ 66.71; **HRMS** m/z calcd for $C_{25}H_{27}FN_3O_3S$ [M+H]+ calc for 468.1752 found 468.1761; **Retention Time** (min) 28.99 (5-95% ACN 0.1% TFA in H_2O 0.1% over 50 minutes) 90%* Purity. *Degradation during purification, isolation and NMR into hydrolysed product (sulfonic acid). **CH_2 peak occluded by peak at 55.7, HSQC cross-peak observed.

4-(2-{5-[(diethylamino)methyl]-2-methoxyphenyl}-1H-pyrrolo[2,3-b]pyridin-4-yl)-2-hydroxybenzaldehyde (6)

To a solution of compound **30** (17 mg, 0.04 mmol, 1 equiv..) in degassed dioxane (0.6 mL, 0.07M) was added compound **20** (12 mg, 0.06 mmol, 1.5 equiv.), 1,1-Bis(diphenylphosphino)ferrocene dichloropalladium complex (2 mg, 0.002 mmol, 5 mol%) and Na_2CO_3 (1M aq, 0.22 mL, 0.2 mmol, 5 equiv.). The solution was purged with nitrogen for 5 mins and then microwaved at 110 ºC for 0.5 h. The reaction was allowed to cool to room temperature and the mixture was evaporated and the resulting residue was purified

using reverse phase flash chromatography an Isolera one with a 25g C18 column (10-70% ACN +0.1% TFA in H_2O + 0.1% TFA) to give compound **6** as a yellow solid (9.6 mg, 51% yield, 98% purity); **^{1}H NMR (400 MHz, $CDCl_3$)** δ 14.06 (s, 1H), 11.35 (s, 1H), 10.08 (s, 1H), 8.21 (d, *J* = 5.9 Hz, 1H), 8.15 (s, 1H), 7.86 (d, *J* = 7.8 Hz, 1H), 7.54 -7.45 (m, 2H,), 7.44 -7.36 (m, 3H), 7.11 (d, *J* = 8.0 Hz, 1H), 4.23 (s, 2H), 4.09 (s, 3H), 3.38 – 3.02 (m, 4H), 1.37 (d, *J* = 7.0 Hz, 6H); **^{13}C NMR** (101 MHz, CDCl3) δ 196.0, 161.8, 158.1, 146.5, 144.1, 142.1, 140.0, 134.4, 133.5, 131.9, 131.3, 124.1, 121.5, 120.3, 119.1, 117.9, 114.7, 112.7, 100.7, 56.0, 55.8, 45.7, 8.2*; **HRMS** m/z calcd for $C_{26}H_{28}N_3O_3$ [M+H]+ calc for 430.2125 found 2430.2126; **IR** (cm^{-1}) 1674, 1432, 1347, 1181, 1132; **Retention Time** (min) 24.56 (5-95% ACN 0.1% TFA in H_2O 0.1% over 50 minutes), 98% purity; *One carbon peak missing, likely occluded by Ar-CH peak at 131.3.

5-(2-{5-[(diethylamino)methyl]-2-methoxyphenyl}-1H-pyrrolo[2,3-b]pyridin-4-yl)-2-hydroxybenzaldehyde (4)

To a solution of compound **30** (17 mg, 0.04 mmol, 1 equiv..) in degassed dioxane (0.6 mL, 0.07M) was added compound **18** (12 mg, 0.06 mmol, 1.5 equiv.), 1,1-Bis(diphenylphosphino)ferrocene dichloropalladium complex (2 mg, 0.002 mmol, 5 mol%) and Na_2CO_3 (1M aq, 0.22 mL, 0.2 mmol, 5 equiv..). The solution was purged with nitrogen for 5 mins and then microwaved at 110 ºC for 0.5 h. The reaction was allowed to cool to room temperature and the mixture was evaporated and the resulting residue was purified using reverse phase flash chromatography an Isolera one with a 25g C18 column (5-95% ACN +0.1% TFA in H_2O + 0.1% TFA) to give compound **4** as a yellow solid (9.24 mg, 49% yield, 99% purity) **^{1}H NMR (400 MHz, DMSO)** δ 12.11 (s, 1H), 11.21 (*br* s, 1H), 10.40 (s, 1H), 9.48 (s, 1H), 8.33 (d, *J* = 5.1 Hz, 1H), 8.12 (d, *J* = 2.4 Hz, 1H), 8.00 (m, 2H), 7.54 (dd, *J* = 8.5, 2.2 Hz, 1H), 7.28 (d, *J* = 8.5 Hz, 1H), 7.25 (s, 1H), 7.24 (d, *J* = 2.4 Hz, 1H), 7.13 (d, *J* = 2.2 Hz, 1H), 4.29 (d, *J* = 4.9 Hz, 2H), 3.97 (s, 3H), 3.22 – 3.04 (m, 4H), 1.26 (t, *J* = 7.2 Hz, 6H) **^{13}C NMR (101 MHz, DMSO)** δ 191.0, 161.2, 157.1, 148.6, 142.4, 139.7, 136.0, 135.5, 132.3, 131.3, 129.2, 128.4, 122.7, 122.2, 120.1, 118.3, 118.2, 114.2, 112.4, 99.8, 55.9, 54.5, 45.8, 8.4; **HRMS** m/z calcd for $C_{26}H_{28}N_3O_3$ [M+H]+ calc for 430.2125 found 430.2132; **Retention Time** (min) 24.14 (5-95% ACN 0.1% TFA in H_2O 0.1% over 50 minutes), 99% purity.

5-(2-{5-[(diethylamino)methyl]-2-methoxyphenyl}-1H-pyrrolo[2,3-b]pyridin-4-yl)-2-ethynylbenzaldehyde (8)

To a solution of compound **30** (40 mg, 0.08 mmol, 1 equiv..) in degassed dioxane (1.47 mL, 0.05M) was added crude compound **24** (0.24 mmol, 3.0 equiv.), Pd(dppf)Cl_2.DCM (4.7 mg, 0.0015 mmol, 5 mol%) and Na_2CO_3 (2M aq, 0.2 mL, 0.4 mmol, 5 equiv..). The solution was purged with nitrogen for 5 mins and then microwaved at 110 ºC for 30 minutes. The reaction was allowed to cool to room temperature and the mixture was evaporated and the resulting residue was purified using reverse phase flash chromatography an Isolera one with a 25g C18 column (5-95% ACN +0.1% TFA in H_2O + 0.1% TFA) to afford 33 mg of the TIPS protected intermediate, which was continued on to the next stage without further purification. To a solution of this intermediate (33 mg, 0.05 mmol, 1 equiv.) in DMF (0.5 mL, 0.1M) was added CsF (84 mg, 0.5 mmol, 10 equiv.). The solution was left to stir at room temperature for 30 minutes, during which the mixture turned dark brown. The solution was concentrated *in vacuo* and the resulting residue was purified using reverse phase flash chromatography an Isolera one with a 25g C18 column (5-95% ACN +0.1% acetic acid in H_2O + 0.1% acetic acid) to afford compound **8** as a yellow solid (7.7 mg, 32% yield, 98% purity); **^{1}H NMR (400 MHz, DMSO)** δ 12.05 (s, 1H), 10.47 (s, 1H), 8.32 (d, *J* = 5.0 Hz, 1H), 8.28 (d, *J* = 2.0 Hz, 1H), 8.15 (dd, *J* = 8.0, 2.0 Hz, 1H), 7.88 (d, *J* = 8.0 Hz, 1H), 7.82 (d, *J* = 2.1 Hz, 1H), 7.34 (dd, *J* = 8.2, 2.1 Hz, 1H), 7.28 (d, *J* = 5.0 Hz, 1H), 7.13 (d, *J* = 8.2 Hz, 1H), 7.11 (d, *J* = 2.0 Hz, 1H), 4.83 (s, 1H), 3.91 (s, 3H), 3.64 (s, 2H), 2.59 – 2.53 (m, 4H), 1.91 (acetic acid salt), 1.02 (t, *J* = 7.0 Hz, 6H); **^{13}C NMR (101 MHz, DMSO)** δ 190.9, 172.0, 155.7, 149.7, 143.1, 139.4, 137.3, 136.6, 134.7, 133.5, 130.1, 129.0, 127.0, 124.0, 119.4, 117.9, 114.4, 111.9, 99.0, 88.6, 79.2, 55.9, 55.6, 45.9, 21.1 (acetic acid), 11.1; **HRMS** m/z calcd for $C_{28}H_{28}N_3O_2$ [M+H]+ calc for 438.2179 found 438.2179; **Retention Time** (min) 26.69 (5-95% ACN 0.1% TFA in H_2O 0.1% over 50 minutes), 98% purity.

4-(2-{5-[(diethylamino)methyl]-2-methoxyphenyl}-1H-pyrrolo[2,3-b]pyridin-4-yl)aniline (31)

To a solution of compound **30** (50 mg, 0.13 mmol, 1 equiv..) in dioxane (1.29 mL, 0.1M) was added Na_2CO_3 (2M aq, 0.32 mL, 0.65 mmol, 5 equiv..) and 4-(4,4,5,5-tetramethyl-[1,3,2]dioxaborolan-2-yl)aniline (42 mg, 0.2 mmol, 1.5 equiv.). The solution was purged with nitrogen for 5 mins and Pd(dppf)Cl_2.DCM (5 mg, 0.0065 mmol, 5 mol%) then microwaved at 110 ºC for 30 minutes. The reaction was allowed to cool to room temperature and the mixture was evaporated and the resulting residue was purified using reverse phase flash

chromatography an Isolera one with a 30g C18 column. This afforded **31** as a yellow solid (42 mg, 81% yield). **^{1}H NMR (400 MHz, MeOD)** δ 8.19 (d, *J* = 6.3 Hz, 1H), 8.01 (d, *J* = 2.3 Hz, 1H), 7.78 (d, *J* = 8.6 Hz, 2H), 7.55 (dd, *J* = 8.5, 2.3 Hz, 1H), 7.48 (d, *J* = 6.3 Hz, 1H), 7.36 (s, 1H), 7.26 (d, *J* = 8.5 Hz, 1H), 7.00 (d, *J* = 8.6 Hz, 2H), 4.33 (s, 2H), 4.05 (s, 3H), 3.24 (p, *J* = 7.5 Hz, 4H), 1.37 (d, *J* = 7.5 Hz, 6H); **^{13}C NMR (101 MHz, MeOD)** δ 159.1, 150.5, 150.2, 142.0, 139.0, 137.4, 134.5, 134.1, 132.4, 131.8, 127.1, 123.6, 120.4, 117.3, 114.9, 113.9, 102.3, 56.7, 56.7, 47.8, 9.0; **HRMS** m/z calcd for $C_{25}H_{28}N_4O$ [M+H]+ calc for 401.2336 found 401.2334.

N-[4-(2-{5-[(diethylamino)methyl]-2-methoxyphenyl}-1H-pyrrolo[2,3-b]pyridin-4-yl)phenyl]prop-2-enamide (12)

To a solution of compound **30** (20 mg, 0.05 mmol, 1.0 equiv.) in anhydrous DMF (500 µL, 0.1 M) was added NEt_3 (21 µL, 0.15 mmol, 3.0 equiv.) and acryloyl chloride (6 µL, 0.075 mmol, 1.5 equiv.). The solution was stirred for 1 hour and quenched with water. The crude mixture was then purified using reverse phase flash chromatography an Isolera one with a 30g C18 column. This afforded **12** as a yellow solid (5.64 mg, 25% yield); **^{1}H NMR (400 MHz, DMSO)** δ 12.01 (s, 1H), 10.41 (s, 1H), 9.39 (s, 1H), 8.34 (s, 1H), 7.99 (d, *J* = 2.2 Hz, 1H), 7.90 (d, *J* = 8.7 Hz, 2H), 7.81 (d, *J* = 8.7 Hz, 2H), 7.52 (dd, *J* = 8.5, 2.2 Hz, 1H), 7.27 (d, *J* = 8.7 Hz, 1H), 7.24 (br s, 1H), 7.15 (s, 1H), 6.50 (dd, *J* = 17.0, 10.1 Hz, 1H), 6.31 (dd, *J* = 17.0, 2.0 Hz, 1H), 5.81 (dd, *J* = 10.1, 2.0 Hz, 1H), 4.29 (d, *J* = 5.0 Hz, 2H), 3.96 (s, 3H), 3.20 – 3.04 (m, 4H), 1.25 (t, *J* = 7.2 Hz, 6H); **^{13}C NMR (101 MHz, DMSO)** δ 163.4, 157.0, 148.9, 142.5, 140.3, 139.5, 135.4, 133.2, 132.2, 131.8, 131.3, 128.8, 127.2, 122.3, 120.2, 119.8, 114.4, 112.4, 99.7, 56.0, 54.6, 45.8, 8.4; **HRMS** m/z calcd for $C_{28}H_{30}N_4O_2$ [M+H]+ calc for 454.2369 found 454.2378; **Retention Time** (min) 23.67 (5-95% ACN 0.1% TFA in H_2O 0.1% over 50 minutes), 99% purity.

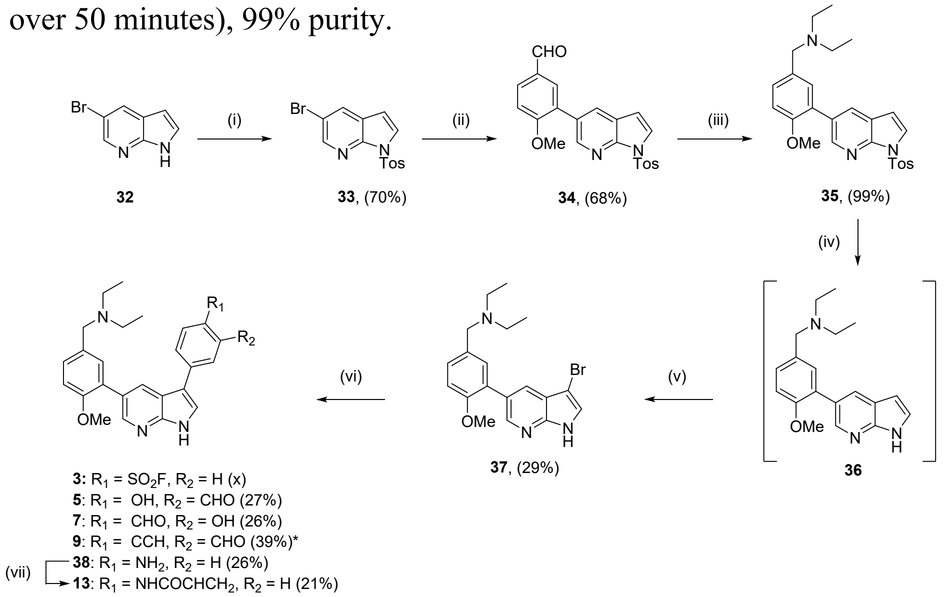

3: R_1 = SO_2F, R_2 = H (x)
5: R_1 = OH, R_2 = CHO (27%)
7: R_1 = CHO, R_2 = OH (26%)
9: R_1 = CCH, R_2 = CHO (39%)*
(vii) **38**: R_1 = NH_2, R_2 = H (26%)
→ **13**: R_1 = $NHCOCHCH_2$, R_2 = H (21%)
S1: R_1 = CO_2H, R_2 = $CH(CH_3)_2$ (76%)

5-Bromo-1-tosyl-1H-pyrrolo[2,3-b]pyridine (33)

To a stirring solution of 5-bromo-1H-pyrrolo[2,3-b]pyridine, **32** (5 g, 25.4 mmol, 1.0 eq) in anhydrous dichloromethane (80 mL) cooled in an ice-water bath to 0 °C was added sodium hydride (60% in mineral oil, 3 g, 76.2 mmol, 3.0 eq) and the mixture was stirred under nitrogen for 30 min. Toluene sulphonyl chloride (9.6 g, 50.8 mmol, 3.0 eq) was added and the mixture was left to warm to rt whilst stirring under nitrogen for 18 h. The reaction mixture was slowly quenched with water and diluted with 1:1 water:DCM and two layers were separated. The aqueous layer was extracted with DCM and the combined organic layers were dried over sodium sulphate and concentrated in vacuo to give a brown solid. This was purified using automated flash column chromatography eluting with 0-60% ethyl acetate:petroleum ether. The desired fractions were combined and concentrated in vacuo to give compound **33** as a white solid (4.4 g, 70%). $\mathbf{R_f}$ **=** 0.34 (20% EtOAc in Petroleum Ether); **^{1}H NMR (400 MHz, $CDCl_3$)** δ 8.33 (d, *J* = 2.2 Hz, 1H), 7.94 (d, *J* = 8.4 Hz, 2H), 7.85 (d, *J* = 2.2 Hz, 1H), 7.63 (d, *J* = 4.0 Hz, 1H), 7.17 (d, J = 8.4 Hz, 2H), 6.43 (d, J = 4.0 Hz, 1H), 2.27 (s, 3H). **^{13}C NMR (101 MHz, $CDCl_3$)** δ 146. 6, 145.6, 145.6, 135.2, 131.9, 129.9, 128.2, 128.1, 124.6, 115.3, 104.6, 21.8.

4-methoxy-3-[1-(4-methylbenzene-1-sulfonyl)-1*H*-pyrrolo[2,3-*b*]pyridin-5-yl]benzaldehyde (34)

To a solution of **33** (0.5 g, 1.4 mmol, 1.0 equiv.) and tetrakis(triphenylphosphine)palladium(0) (0.082 g, 0.07 mmol, 0.05 equiv..) in 1,4-dioxane was added 5-formyl-2-methoxyphenyl boronic acid (0.384 g, 2.1 mmol, 1.5 equiv.) under a nitrogen atmosphere. Aqueous sodium carbonate (2 M, 5.0 mL, 9.8 mmol, 7.0 equiv.) was then added and the reaction mixture left to stir at 110 ºC for 18 hrs. Solvent was removed under vacuum and the crude was dissolved in ethyl acetate, poured into water and extracted with ethyl acetate. The organic layer was washed with brine before drying over magnesium sulphate and purified by flash column chromatography (0-100% ethyl acetate-petroleum ether) to afford **34** as a yellow foam (0.467 g, 68%); $\mathbf{R_f}$: 0.58 (50% EtOAc in petroleum ether); **^{1}H NMR (400 MHz, $CDCl_3$)** δ 9.84 (s, 1H), 8.48 (d, *J* = 2.1 Hz, 1H), 8.02 (d, *J* = 8.4, 2H), 7.90 (d, *J* = 2.1 Hz, 1H), 7.80 (dd, *J* = 8.5, 2.1 Hz, 1H), 7.73 (d, *J* = 2.1 Hz, 1H), 7.66 (d, *J* = 4.0 Hz, 1H), 7.20 (d, *J* = 8.4 Hz, 2H), 7.03 (d, *J* = 8.5 Hz, 1H), 6.53 (d, J = 4.0 Hz, 1H),

3.81 (s, 3H), 2.28 (s, 3H); ^{13}C NMR (101 MHz, $CDCl_3$) δ 190.8, 161.7, 146.5, 145.9, 145.4, 135.6, 132.4, 132.3, 130.5, 130.2, 129.9, 128.5, 128.5, 128.3, 127.1, 122.6, 111.3, 105.5, 56.2, 21.8; **HRMS** *m/z calcd* for $C_{22}H_{18}N_2O_4S$ [M+H]+ 407.1060 *found* 407.1069

diethyl({4-methoxy-3-[1-(4-methylbenzenesulfonyl)-1H-pyrrolo[2,3-b]pyridin-5-yl]phenyl}methyl)amine (35)

To a solution of compound **34** (2.43 g, 5.9 mmol, 1 equiv.) in anhydrous dichloroethane (30 mL, 0.25) was added Titanium isopropoxide (3.5 mL, 11.9 mmol, 2 equiv.), diethylamine (1.86 mL, 17.91 mmol, 3 equiv.) and sodium triacetoxyborohydride (3.7 g, 14.9 mmol, 2.5 equiv.). The solution was left to stir for 3 hours at room temperature, when a further 1 equiv.alent (1.27 g, 5.97 mmol) of sodium triacetoxyborohydride was added. The resulting solution was stirred for 18 hours at room temperature, after which ammonium hydroxide (10 mL) was added to quench the reaction. The solution was partitioned the dichloroethane and water (20 mL), and the aqueous layer was washed with DCM (2 x 20 mL) and dried over magnesium sulfate. The filtered solution was concentrated *in vacuo* to give a brown oil (2.74 g, 99%); $\mathbf{R_f}$ 0.35 (10% MeOH in DCM); **^{1}H NMR (400 MHz, $CDCl_3$)** δ 8.57 (d, *J* = 2.1 Hz, 1H), 8.06 (d, *J* = 8.4 Hz, 2H), 7.98 (d, *J* = 2.1 Hz, 1H), 7.67 (d, *J* = 4.0 Hz, 1H), 7.30 – 7.23 (m, 2H), 7.20 (d, *J* = 8.4 Hz, 2H), 6.89 (d, *J* = 9.0 Hz, 1H), 6.56 (d, *J* = 4.0 Hz, 1H), 3.73 (s, 3H), 3.52 (s, 2H), 2.50 (q, *J* = 7.1 Hz, 4H), 2.28 (s, 3H), 1.01 (t, *J* = 7.1 Hz, 6H); **^{13}C NMR (101 MHz, $CDCl_3$)** δ 155.1, 145.7, 145.7, 144.7, 135.1, 131.7, 131.2, 129.9, 129.4, 129.2, 127.6, 126.6, 126.2, 121.9, 110.6, 105.2, 56.3, 55.2, 46.2, 21.1, 11.3; HRMS *m/z calcd* for $C_{26}H_{29}N_3O_3S$ [M+H]+ 464.2002 *found* 464.2005

[(3-{3-bromo-1H-pyrrolo[2,3-b]pyridin-5-yl}-4-methoxyphenyl)methyl]diethylamine (37)

500 mg of **35** (1.0 mmol, 1 equiv.) was dissolved in MeOH (10.0 mL, 0.1 M) and treated with KOH (300 mg, 5.0 mmol, 5 equiv.) and heated at reflux for 18 hours. The solution was cooled to room temperature, diluted with DCM (90 mL), filtered and concentrated *in vacuo*. Half of the resulting residue (0.5 mmol) was then dissolved in DCM (5.0 mL, 0.1 M) and subjected to *N*-bromo-succinimide (96 mg, 0.5 mmol, 1 equiv.) and stirred at room temperature. After 2 hours, the solution was concentrated *in vacuo* and purified by reverse phase automated column chromatography using an isolera one to give compound **37** as an amorphous off-white solid (123 mg, 29% yield); $\mathbf{R_f}$ 0.26 (10% MeOH in DCM); **^{1}H NMR (400 MHz, $CDCl_3$)** δ 13.66 (s, 1H), 11.87 (br s, 1H,), 8.49 (d, *J* = 1.7 Hz, 1H), 8.46 (d, *J* =

1.7 Hz, 1H, H-4), 7.60 – 7.51 (m, 3H), 7.09 (d, *J* = 8.4 Hz, 1H), 4.23 (s, 2H), 3.88 (s, 3H), 3.26 (m, 2H), 3.18 – 3.04 (m, 2H), 1.37 (t, *J* = 7.3 Hz, 6H); **^{13}C NMR (101 MHz, $CDCl_3$)** δ 157.6, 140.1, 135.7, 135.6, 133.3, 133.2, 128.6, 126.4, 125.6, 124.7, 121.9, 112.3, 91.4, 56.1, 55.6, 45.7, 8.4; HRMS *m/z calcd* for $C_{19}H_{22}BrN_3O$ [M+H]+ 388.1019 *found* 388.1013; **Retention Time** (min) 4.88 (5-95% ACN 0.1% TFA in H_2O 0.1% over 10 minutes).

4-(5-{5-[(diethylamino)methyl]-2-methoxyphenyl}-1H-pyrrolo[2,3-b]pyridin-3-yl)benzene-1-sulfonyl fluoride (3)

To a solution of compound **37** (50 mg, 0.13 mmol, 1 equiv.) in degassed dioxane (0.6 mL, 0.07M) was added compound **16** (82 mg, 0.52 mmol, 4.0 equiv.), 1,1-Bis(diphenylphosphino)ferrocene dichloropalladium complex (3.6 mg, 0.007 mmol, 5 mol%) and K_3PO_4 (2M aq, 0.160 mL, 0.33 mmol, 2.5 equiv..). The solution was purged with nitrogen for 5 mins and then microwaved at 110 ºC for 3 h. The reaction was allowed to cool to room temperature and the mixture was evaporated and the resulting residue was purified using reverse phase flash chromatography an Isolera one with a 25g C18 column (5-95% ACN +0.1% acetic acid in H_2O + 0.1% acetic acid) to give compound **3** as a yellow solid (6.5 mg, 24% yield, 95% purity); **^{1}H NMR (400 MHz, DMSO)** δ 8.48-8.41 (m, 2H), 8.31 (d, *J* = 2.7 Hz, 1H), 8.16 (d, *J* = 8.8 Hz, 2H), 8.16 (d, *J* = 8.8 Hz, 2H), 7.57 – 7.51 (m, 1H), 7.47 (d, *J* = 8.6 Hz, 1H), 7.21 (d, *J* = 8.6 Hz, 1H), 4.11 (br s, 2H), 3.82 (s, 3H), 2.93 (br s, 4H), 1.15 (br s, J = 8.6 Hz, 6H)*; **^{13}C NMR (101 MHz, DMSO)** δ 148.4, 144.4, 143.5, 133.5, 129.2, 128.3, 128.0, 127.8, 127.6, 127.2, 126.9, 125.5, 124.4, 116.4, 112.2, 111.8, 55.8, 45.8, 24.7, 9.1**; **^{19}F NMR (376 MHz, DMSO)** δ 67.27; **HRMS** m/z calcd for $C_{25}H_{27}FN_3O_3S$ [M+H]+ calc for 468.1752 found 468.1749; **Retention Time** (min) 29.25 (5-95% ACN 0.1% TFA in H_2O 0.1% over 50 minutes) 90% Purity.*Sample degradation during NMR.**CSO_2F not visible due to ^{19}F splitting.

4-(5-{5-[(diethylamino)methyl]-2-methoxyphenyl}-1H-pyrrolo[2,3-b]pyridin-3-yl)-2-hydroxybenzaldehyde (7)

To a solution of compound **37** (20 mg, 0.04 mmol, 1 equiv..) in degassed dioxane (0.7 mL, 0.06M) was added compound **20** (30 mg, 0.12 mmol, 3.0 equiv.), 2nd Generation XPhos Precatalyst (1.6 mg, 0.0015 mmol, 5 mol%) and Na_2CO_3 (2M aq, 0.1 mL, 0.16 mmol, 5 equiv..). The solution was purged with nitrogen for 5 mins and then microwaved at 110 ºC for 6 h. 11

5-(5-{5-[(diethylamino)methyl]-2-methoxyphenyl}-1H-pyrrolo[2,3-b]pyridin-3-yl)-2-hydroxybenzaldehyde (5)

To a solution of compound **37** (20 mg, 0.04 mmol, 1 equiv..) in degassed dioxane (0.7 mL, 0.06M) was added compound **18** (30 mg, 0.12 mmol, 3.0 equiv.), 2nd Generation XPhos Precatalyst (1.6 mg, 0.0015 mmol, 5 mol%) and Na_2CO_3 (2M aq, 0.1 mL, 0.16 mmol, 5 equiv..). The solution was purged with nitrogen for 5 mins and then microwaved at 110 ºC for 6 h. The reaction was allowed to cool to room temperature and the mixture was evaporated and the resulting residue was purified using reverse phase flash chromatography an Isolera one with a 25g C18 column (5-95% ACN +0.1% TFA in H_2O + 0.1% TFA) to give compound **5** as an off-white solid (8.1 mg, 27% yield, 98% purity); **^{1}H NMR (400 MHz, DMSO)** δ 11.99 (d, *J* = 2.7 Hz, 1H), 10.78 (s, 1H), 10.34 (s, 1H), 9.39 (s, 1H), 8.41 (d, *J* = 2.0 Hz, 1H), 8.31 (d, *J* = 2.0 Hz, 1H), 7.97 (d, *J* = 2.4 Hz, 1H), 7.89 (dd, *J* = 8.6, 2.4 Hz, 1H), 7.87 (d, *J* = 2.7 Hz, 1H), 7.60 (d, *J* = 2.3 Hz, 1H), 7.52 (dd, *J* = 8.5, 2.3 Hz, 1H), 7.24 (d, *J* = 8.5 Hz, 1H), 7.11 (d, *J* = 8.6 Hz, 1H), 4.31 (d, *J* = 5.0 Hz, 2H), 3.85 (s, 3H), 3.19 – 3.03 (m, 4H), 1.24 (t, *J* = 7.2 Hz, 6H); **^{13}C NMR (101 MHz, DMSO)** δ 191.6, 159.1, 157.1, 148.0, 143.6, 134.7, 133.5, 131.6, 128.3, 127.7, 126.4, 126.0, 125.5, 123.9, 122.5, 122.3, 118.1, 116.7, 113.4, 112.0, 55.8, 54.5, 45.7, 8.4; **HRMS** m/z calcd for $C_{26}H_{28}N_3O_3$ [M+H]+ calc for 430.2125 found 430.2128; **Retention Time** (min) 24.88 (5-95% ACN 0.1% TFA in H_2O 0.1% over 50 minutes), 98% purity.

5-(5-{5-[(diethylamino)methyl]-2-methoxyphenyl}-1H-pyrrolo[2,3-b]pyridin-3-yl)-2-ethynylbenzaldehyde (9)

To a solution of compound **37** (47 mg, 0.09 mmol, 1 equiv..) in degassed dioxane (1.2 mL, 0.08M) was added crude compound **24** (0.28 mmol, 3.0 equiv.), 2nd Generation XPhos Precatalyst (5.0 mg, 0.0015 mmol, 5 mol%) and Na_2CO_3 (2M aq, 0.3 mL, 0.4 mmol, 5 equiv..). The solution was purged with nitrogen for 5 mins and then microwaved at 110 ºC for 30 minutes. The reaction was allowed to cool to room temperature and the mixture was evaporated and the resulting residue was purified using reverse phase flash chromatography an Isolera one with a 25g C18 column (5-95% ACN +0.1% TFA in H_2O + 0.1% TFA) to afford 22 mg of the TIPS protected intermediate, which was continued on to the next stage without further purification.

To a solution of this protected intermediate (22 mg, 0.04 mmol, 1 equiv..) in DMF (0.4 mL, 0.1M) was added CsF (56 mg, 0.4 mmol, 10 equiv..). The solution was left to stir at room temperature for 30 minutes. The solution was concentrated *in vacuo* and the resulting residue was purified by preparative reverse phase HPLC (5-95% ACN +0.1% acetic acid in H_2O + 0.1% acetic acid) to afford compound **9** as a yellow solid (6.2 mg, 39% yield, 98% purity); **^{1}H NMR (400 MHz, DMSO)** δ 12.21 (d, *J* = 2.7 Hz, 1H), 10.43 (s, 1H), 8.39 (d, *J* = 2.0 Hz, 1H), 8.35 (d, *J* = 2.0 Hz, 1H), 8.18 (d, *J* = 2.0 Hz, 1H), 8.15 (d, *J* = 2.7 Hz, 1H), 8.09 (dd, *J* = 8.1, 2.0 Hz, 1H), 7.73 (d, *J* = 8.1 Hz, 1H), 7.39 (s, 1H), 7.35 (d, *J* = 8.4 Hz, 1H), 7.13 (d, *J* = 8.4 Hz, 1H), 4.68 (s, 1H), 3.81 (s, 3H), 3.71 (s, 2H), 2.61 (s, 4H), 1.91 (acetic acid salt), 1.04 (t, *J* = 7.1 Hz, 6H); **^{13}C NMR (101 MHz, DMSO)** δ 191.2, 172.0, 155.6, 148.2, 144.1, 136.6, 136.4, 134.6, 131.6, 131.4, 129.6, 127.7, 127.5, 126.7, 126.0, 124.2, 121.1, 116.4, 112.5, 111.7, 87.4, 79.6, 55.7, 45.9, 21.1 (acetic acid salt), 11.0;* **HRMS** m/z calcd for $C_{28}H_{27}N_3O_2$ [M+H]+ calc for 438.2176 found 438.2184; **Retention Time** (min) 27.45 (5-95% ACN 0.1% TFA in H_2O 0.1% over 50 minutes), 98% purity.* CH2 not observed.

4-(5-{5-[(diethylamino)methyl]-2-methoxyphenyl}-1H-pyrrolo[2,3-b]pyridin-3-yl)aniline (38)

To a solution of compound **37** (50 mg, 0.13 mmol, 1 equiv.) in dioxane (1.29 mL, 0.1M) was added Na_2CO_3 (2M aq, 0.32 mL, 0.65 mmol, 5 equiv.) and compound 4-(4,4,5,5-tetramethyl-[1,3,2]dioxaborolan-2-yl)aniline (42 mg, 0.2 mmol, 1.5 equiv.). The solution was purged with nitrogen for 5 mins and Pd(dppf)Cl_2.DCM (5 mg, 0.0065 mmol, 5 mol%) then microwaved at 110 ºC for 30 minutes. The reaction was allowed to cool to room temperature and the mixture was stirred at room temperature for 72 hours. The crude mixture was then evaporated and the resulting residue was purified using reverse phase flash chromatography an Isolera one with a 30g C18 column. This afforded **38** as an off-white solid (13.3 mg, 26% yield). **^{1}H NMR (400 MHz, MeOD)** δ 8.53 (d, *J* = 1.7 Hz, 1H), 8.47 (br s, 1H), 7.88 – 7.83 (m, 3H), 7.60 (d, *J* = 2.4 Hz, 1H), 7.55 (dd, *J* = 8.5, 2.4 Hz, 1H), 7.48 (d, *J* = 8.6 Hz, 2H), 7.25 (d, *J* = 8.5 Hz, 1H), 4.36 (s, 2H), 3.89 (s, 3H), 3.28 – 3.19 (m, 4H), 1.37 (t, *J* = 7.3 Hz, 6H); **^{13}C NMR (101 MHz, MeOD)** δ 159.4, 147.5, 143.3, 137.4, 136.5, 134.7, 133.7, 131.6, 130.8, 129.9, 129.4, 126.2, 124.4, 123.2, 121.5, 116.3, 113.3, 56.8, 56.4, 47.7, 9.0; **HRMS** m/z calcd for $C_{25}H_{28}N_4O$ [M+H]+ calc for 401.2263 found 401.2264

N-[4-(5-{5-[(diethylamino)methyl]-2-methoxyphenyl}-1H-pyrrolo[2,3-b]pyridin-3-yl)phenyl]prop-2-enamide (13)

To a solution of compound **37** (13.3 mg, 0.33 mmol, 1.0 equiv.) in anhydrous DMF (335 µL, 0.1 M) was added NEt_3 (13 µL, 0.10 mmol, 3.0 equiv.) and acryloyl chloride (4 µL, 0.05 mmol, 1.5 equiv.). The solution was stirred for 30 minutes and quenched with water. The crude mixture was then purified using reverse phase flash chromatography an Isolera one with a 30g C18 column. This afforded **13** as a yellow solid (3.23 mg, 21% yield); **^{1}H NMR (400 MHz, DMSO)** δ 11.97 (s, 1H), 10.26 (s, 1H), 9.58 (s, 1H), 8.41 (s, 1H), 8.35 (d, *J* = 1.9 Hz, 1H), 7.86 (d, *J* = 2.6 Hz, 1H), 7.75 (d, *J* = 8.8 Hz, 2H), 7.69 (d, *J* = 8.8 Hz, 2H), 7.62 (d, *J* = 2.3 Hz, 1H), 7.52 (dd, *J* = 8.5, 2.3 Hz, 1H), 7.22 (d, *J* = 8.5 Hz, 1H), 6.46 (dd, *J* = 17.0, 10.1 Hz, 1H), 6.26 (dd, *J* = 17.0, 2.0 Hz, 1H), 5.76 (dd, *J* = 10.1, 2.0 Hz, 1H), 4.29 (d, *J* = 5.3 Hz, 2H), 3.82 (s, 3H), 3.17 – 3.00 (m, 4H), 1.24 (t, *J* = 7.2 Hz, 6H); **^{13}C NMR (101 MHz, DMSO)** δ 163.2, 157.2, 148.0, 143.8, 137.0, 133.7, 132.0, 131.7, 130.4, 128.5, 128.1, 126.9, 126.8, 125.7, 124.0, 122.5, 120.1, 117.0, 114.4, 112.0, 55.9, 54.6, 45.7, 8.4. **HRMS** m/z calcd for $C_{28}H_{30}N_4O_2$ [M+H]+ calc for 455.2441 found 455.2442; **Retention Time** (min) 23.24 (5-95% ACN 0.1% TFA in H_2O 0.1% over 50 minutes), 99% purity.

4-(5-(5-((diethylamino)methyl)-2-methoxyphenyl)-1H-pyrrolo[2,3-b]pyridin-3-yl)-2-isopropylbenzoic acid, S1

To a 10 mL microwave vial compound **37** (1 equiv) in 1,4- dioxane was added 2-isopropyl-4-(4,4,5,5-tetramethyl-1,3,2-dioxaborolan-2-yl)benzoic acid (1.1 equiv), Pd(dppf)Cl_2·DCM complex (0.05 equiv.) under a nitrogen atmosphere. The solution was purged with nitrogen for 5 mins and the reaction microwaved at 110 ºC for 0.5 h. The reaction was allowed to cool to room temperature and the mixture was filtered through celite eluting with methanol. The filtrate was evaporated and the resulting residue was purified by preparative HPLC (10-95% MeCN/H_2O + 0.1% TFA) to give **S1** in a (19 mg, 76%); ^{1}H NMR (400 MHz, CD_3OD) δ: 8.49 (d, *J* = 2.0 Hz, 1H), 8.41 (d, *J* = 1.9 Hz, 1H), 7.88 (d, *J* = 8.1 Hz, 1H), 7.83 (s, 1H), 7.77 (d, *J* = 1.7 Hz, 1H), 7.62 – 7.55 (m, 2H), 7.53 (dd, *J* = 8.4, 2.4 Hz, 1H), 7.26 (d, *J* = 8.5 Hz, 1H), 4.36 (s, 2H), 3.98 (h, *J* = 7.2 Hz, 1H), 3.91 (s, 3H), 3.24 (p, *J* = 7.2 Hz, 4H), 1.41 – 1.27 (m, 12H); ^{13}C NMR (101 MHz, CD_3OD) δ: 158.0, 150.4, 133.2, 131.6, 130.7, 127.6, 126.1, 124.6, 124.1, 123.4, 121.8, 111.9, 55.5, 55.1, 29.1, 23.1, 7.6; HRMS *m/z calcd* for $C_{29}H_{34}N_3O_3$ $[M+H]^+$ 472.2522 *found* 472.2576.

Protein purification

As previously described full-length PfCLK3 construct was expressed in E. coli strain C43 (DE3). (5) Protein was purified using IMAC, TEV cleavage, and a second IMAC step before

dialyzing the protein into a final buffer containing 20 mM HEPES pH 7.4, 150 mM NaCl, 1 mM TCEP and 1 mM MgCl2.

*Pf*CLK3 kinase domain (residues 334–699 with a C-terminal TEV cleavage sequence and His6-tag) was cloned into pFastBac vector and expressed and purified from Sf21 insect cells. Cells were infected using P2 BIICs at an MOI of 0.2 and left to express for 72 h. Harvested cells were lysed and centrifuged before purifying using IMAC and SEC in a final buffer containing 20 mM HEPES pH 7.4, 150 mM NaCl, 1 mM TCEP, and 1 mM MgCl2.

Protein Mass Spectrometry

For intact protein mass spectrometry, compounds were incubated with a 5-fold excess of compound in buffer containing 20 mM HEPES pH 7.4, 150 mM NaCl, 1 mM TCEP, and 1 mM $MgCl_2$. 5 μL of kinase domain (1 mg/mL) was added to 25 μL of buffer before 0.12 μL of compound in a 10 mM DMSO stock was added to start the incubation. Samples were incubated at room temperature for 4 hours before 10 μL alliquots were analysed by LC-MS. Intact protein LC-MS experiments were performed on a Synapt G2 Q-ToF instrument equipped with electrospray ionisation (Waters Corp., Manchester, UK). LC separation was achieved using an Acquity UPLC equipped with a reverse phase C4 Aeris Widepore 50 × 2.1 mm HPLC column (Phenomenex, CA, USA) and a gradient of 5–95% acetonitrile (0.1% formic acid) over 10 minutes was employed. Data analysis was performed using MassLynx v4.1 and deconvolution was performed using MaxEnt.

After incubation with a 5-fold excess of compound as described above, 1.2 μL Fmoc-Lys-OH.HCl (final concentration 250 mM) was added to quench excess inhibitor. 2 μL of Pierce trypsin protease (1 mg/mL) was added to give a final protein:trypsin ratio of 10:1. Overnight incubation at 37 °C afforded a series of peptides, which were prepared using PierceTM C18 spin columns according to the manufacturer's procedure.

The resulting peptide mixture was analyzed by high resolution nESI FT-ICR MS using a 12 Telsa Solarix 2XR mass spectrometer (Bruker Daltonics) equipped with a nanomate infusion robot (Advion Biosciences). The resulting mass spectra were then processed by using the SNAP algorithm in Data Analysis (Bruker Daltonics) to produce monoisotopic mass lists. The mass lists were then searched against the primary amino acid sequence of *Pf*CLK3 kinase domain$_{343–699}$ using MS-Fit in Protein Prospector (University of California, San

Francisco) and ProSight Lite v1.4 (Northwestern University). For all analyses, error tolerances of 10 ppm were used.

Time Resolved Förster Resonance Energy Transfer (TR-FRET) Assay

Tr-FRET technology was used to determine kinase activity and Km ATP before profiling compounds. An 11-point half-log serial dilution of each test compound was prepared in 100% DMSO at 100X final test concentration. The dilution series were dispensed onto assay plates with liquid handling machinery using positive displacement. The final DMSO concentration in the assay was 1%. To obtain IC_{50} values, full length recombinant PfCLK3 was prepared in 1X assay buffer (50 mM Hepes, 10 mM MgCl2, 1 mM EGTA, 0.01% Tween20, 2 mM TCEP) at a 2X concentration and incubated with compounds for 15 minutes at room temperature before addition of ULight-labelled peptide substrate (MBP) with Km ATP (at 2X concentration). The reaction was centrifuged and incubated for 2 hr at 37 °C. To stop the reaction, 1X Lance detection buffer was added with 1 nM Eu-labelled anti-MBP and 10 mM EDTA, and the reaction was incubated in the dark for 2 hr at room temperature to allow detection to take place. Plate output was read on a Pherastar FSX and data analysed in Graphpad Prism to a 4-parameter curve fit. Reactions were normalised to kinase and no kinase control wells

P. falciparum (3D7) culture and synchronisation.

P. falciparum cultures were maintained in RPMI-1640 media (Invitrogen) supplemented with 0.2% sodium bicarbonate, 0.5% Albumax II, 2.0 mM L-glutamine (Sigma) and 10 mg/L gentamycin. For continuous culture, the parasites were maintained at 4% haematocrit in human erythrocytes from 0+ blood donors and between 0.5 - 3% parasitaemia in an incubator at 37 °C, 5% carbon dioxide (CO_2), 5% oxygen (O_2) and 90% nitrogen (N_2). To obtain highly synchronous ring stage parasites for assays, cultures were double synchronised using Percoll and Sorbitol synchronisation. First, highly segmented schizonts were enriched by centrifugation on a 70% Percoll (GE Healthcare) cushion gradient. The Schizont pellet was collected and washed twice before fresh erythrocytes were added to a final haematocrit of 4%, and incubated for about 1-2 hours shaking continuously to allow merozoites egress and re-invasion of new erythrocytes. Residual schizonts were then removed by a second Percoll purification followed by treating the ring pellet with sorbitol to generate highly synchronous 1-2 hours old ring-stage parasites.

Ex vivo *P. Falciparum* (3D7) inhibition assay.

To determine the IC_{50} of the molecules in parasites (*P. falciparum* 3D7) ex vivo, the molecules were diluted 1 in 3 from a starting concentration of 100 μM for 12 dilution points in triplicate. 50 μL of freshly diluted drugs, at twice the required final concentrations were aliquoted into black 96-well plates. To the compound plates, 50 μL of parasites prepared at 8% haematocrit at a parasitaemia (0.3 - 0.5%) were added and mixed by pipetting up and down several times giving a final culture volume of 100 μL at the required compound concentration (top concentration of 100 μM) and 4% haematocrit. To the 'no compound' control, growth media was added and uninfected erythrocytes were included on the plate as blank. The outer wells were filled with media to reduce evaporation from the experimental wells and the plates incubated for 72 hours (±2 hours) to allow the parasites sufficient time to re-invade before they are collected and frozen. For cellular washout studies, compound media was exchanged for compound-free media after 6 hours, and the parasites incubated for a further 66 hours. To quantify growth inhibition, the plates were thawed at room temperature for at least 1 hour and 100 μL of lysis buffer (20 mM Tris-HCl; 5 mM EDTA; 0.004% saponin and triton X-100) in PBS containing Sybr Green I (1μL in 5 ml) was added to each well and mixed by pipetting up and down several times and incubated for 1 hour in the dark shaking. Using a Fluroskan/ClarioStar plate reader at excitation of 485 nm and emission of 538 nm, plate absorbances were acquired. The data was normalised against the controls and graphs were generated using Graph Pad Prism 8 to determine the IC_{50} values using the non-linear regression log (inhibitor) versus response (three parameter) curve. All experiments were performed 3 times.

Human cell viability assay.

Mycoplasma tested HepG2 cells were cultured in Dulbecco's Modified Eagle Medium (DMEM) with 10% fetal bovine serum, 1% non-essential AA, 1% sodium pyruvate, 1% Pen-strep and 100ug/mL Normocin. Cultures were incubated at 37 °C, 5% CO2. Cells were detached using 0.05% trypsin-EDTA. 500nL of drug compound dilutions, in triplicate, were added to 384-well, black, clear bottom assay plates using a Mosquito liquid handling machine. Assay plates, containing compound dilutions, were seeded at 5,000 cells/well and incubated for 48 hours. 40uM final concentration resazurin, diluted in DPBS, was added to the assay plates which were then incubated for 4 hours and analysed using a ClariostarTM

plate reader to measure fluorescence Intensity (545-20nm/600-40nm). 3 control compounds were included on every assay plate, Tamoxifen, Puromycin and TCMDC-135051. Maximum signal control was obtained from wells with DMSO only and minimum signal control with Tamoxifen 100uM. These were used to normalise data and give percentage inhibition of metabolic activity. Experiments were performed N=3 and normalised data was grouped and a nonlinear regression curve with four parameters was plotted using GraphPad Prism, generating activity data.

***Pf*CLK3 Activity at Elevated ATP Concentrations**

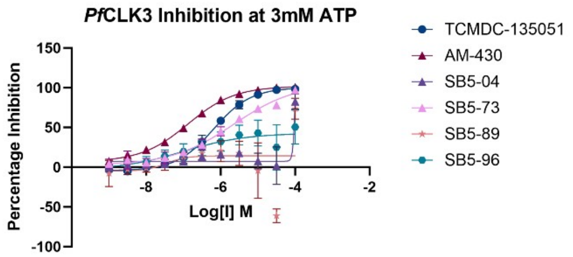

Compound	pIC_{50} (*Pf*CLK3) 3 mM ATP	pIC_{50} (*Pf*CLK3) 3 mM ATP
TCMDC-135051 (**1**)	640 nM	6.19
AM-430 (**x**)	132 nM	6.88
SB5-04 (**4**)	Inactive	Inactive
SB5-73 (**5**)	2.25 μM	5.65
SB5-89 (**8**)	Inactive	Inactive
SB5-96 (**9**)	Inactive	Inactive

Figure S1: PfCLK3 activity at 3 mM ATP assay concentrations

NMR Spectra for Novel Compounds

^{1}H and [13]C Spectra ($CDCl_3$) for compound 23

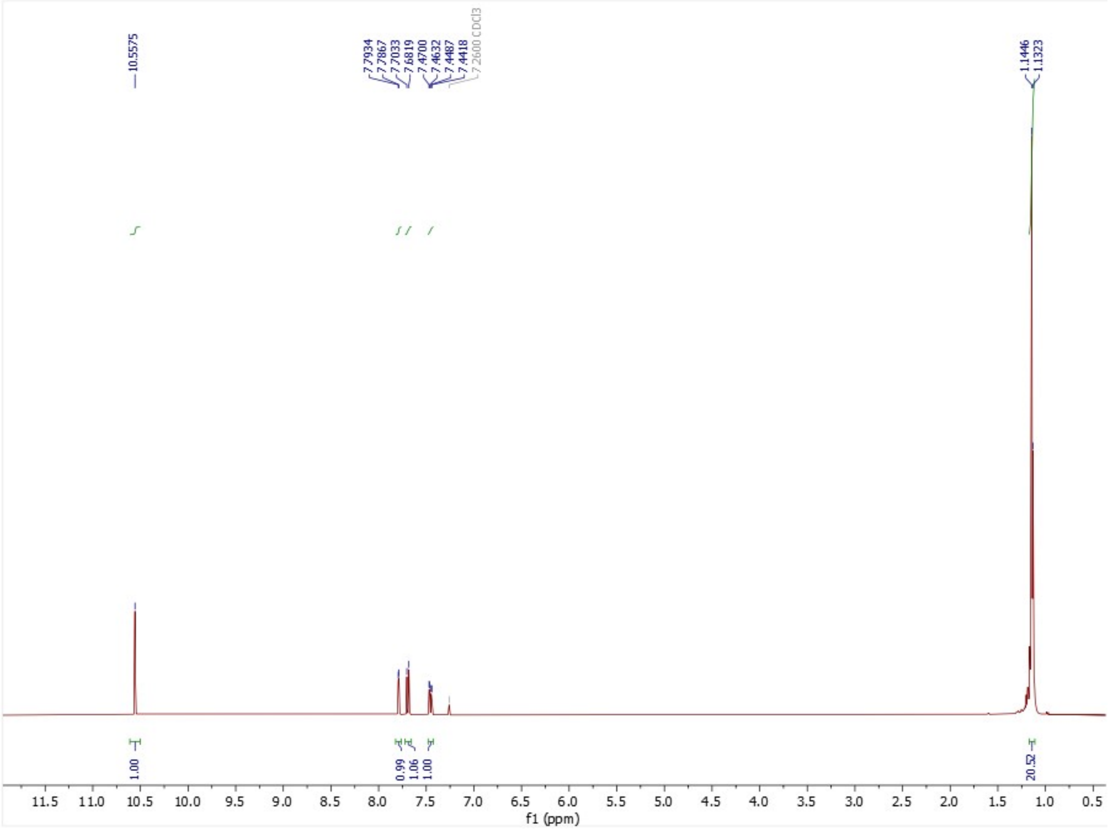

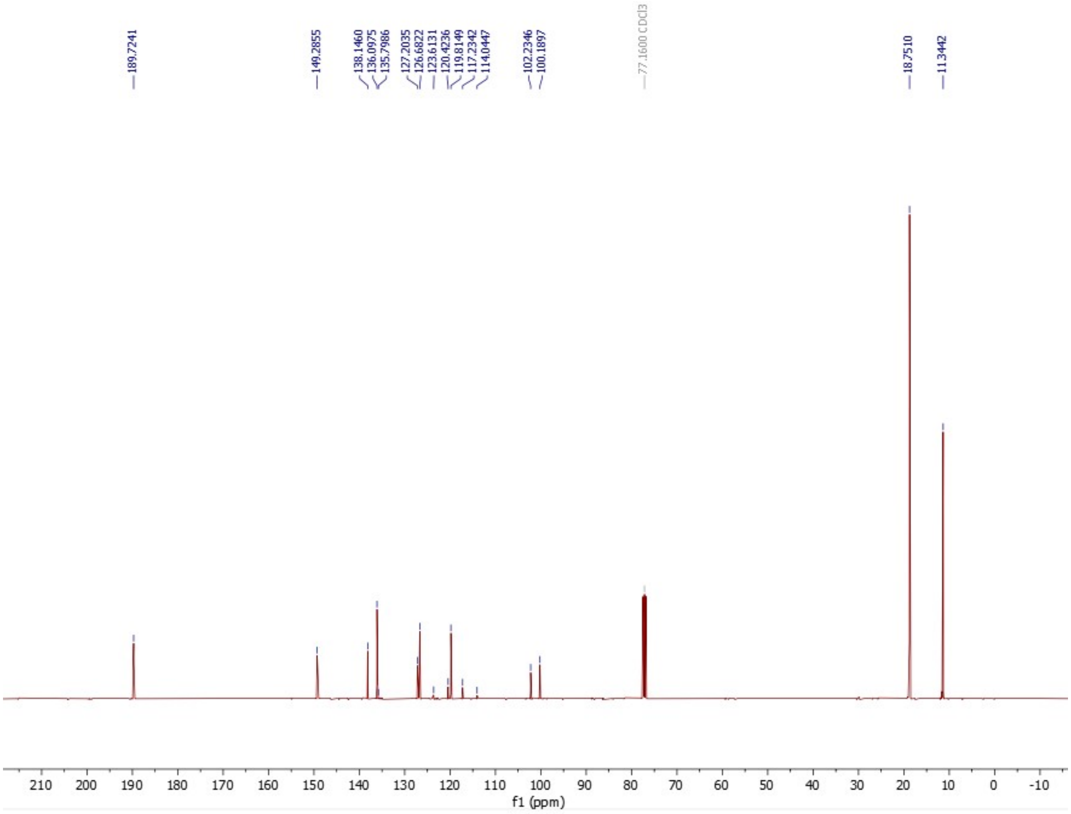

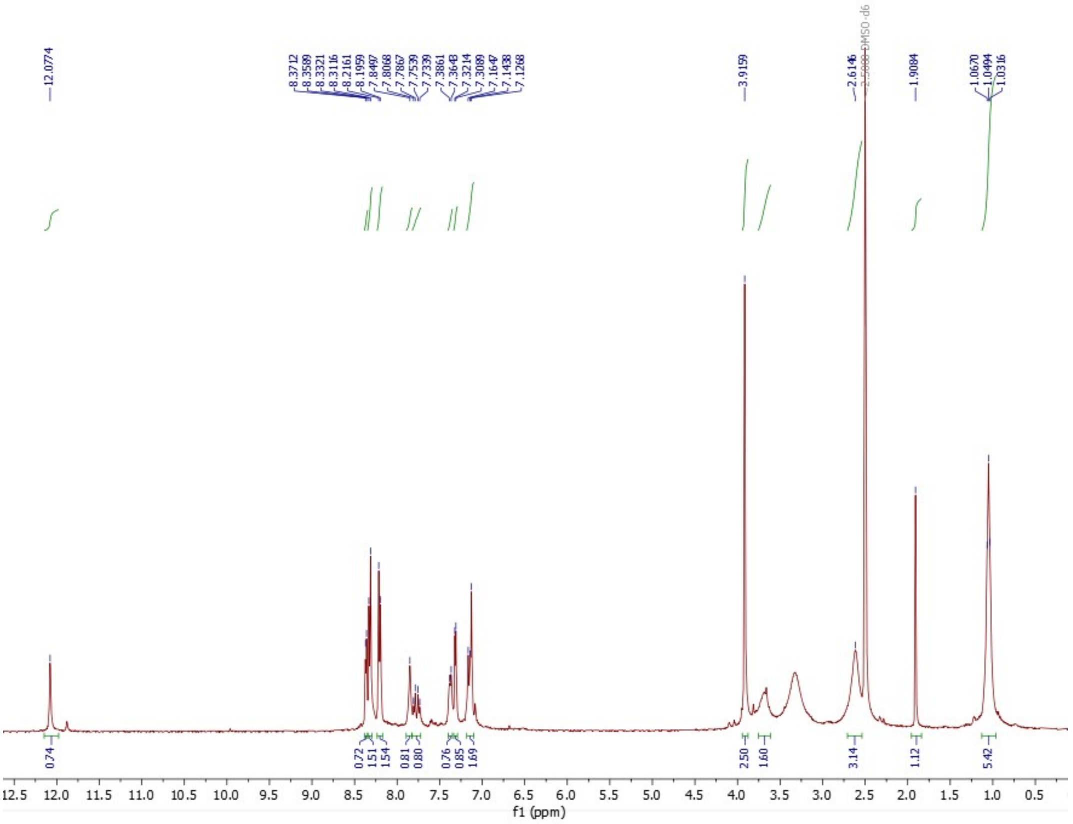

^{1}H and ^{13}C Spectra ($CDCl_3$) for compound 2

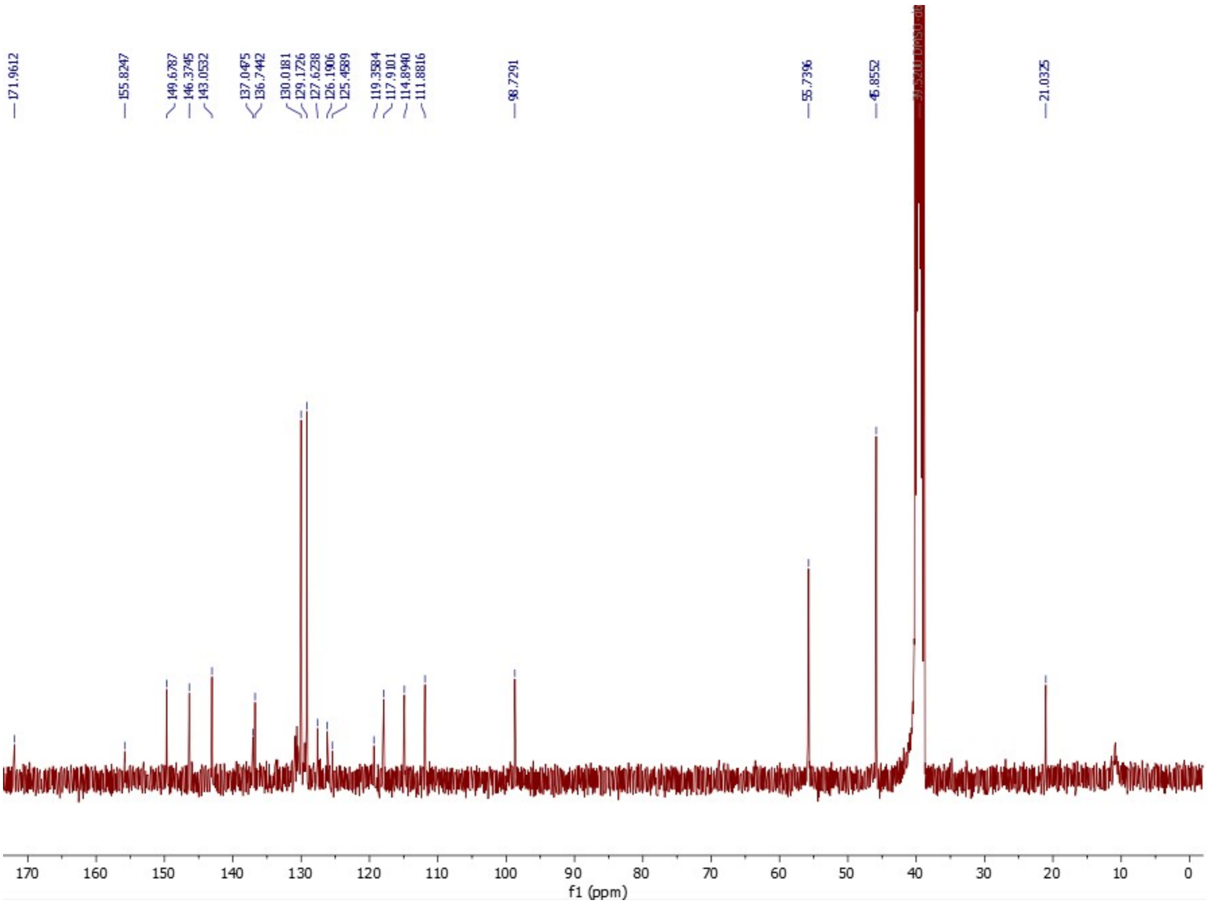

^{1}H and ^{13}C Spectra ($CDCl_3$) for compound 6

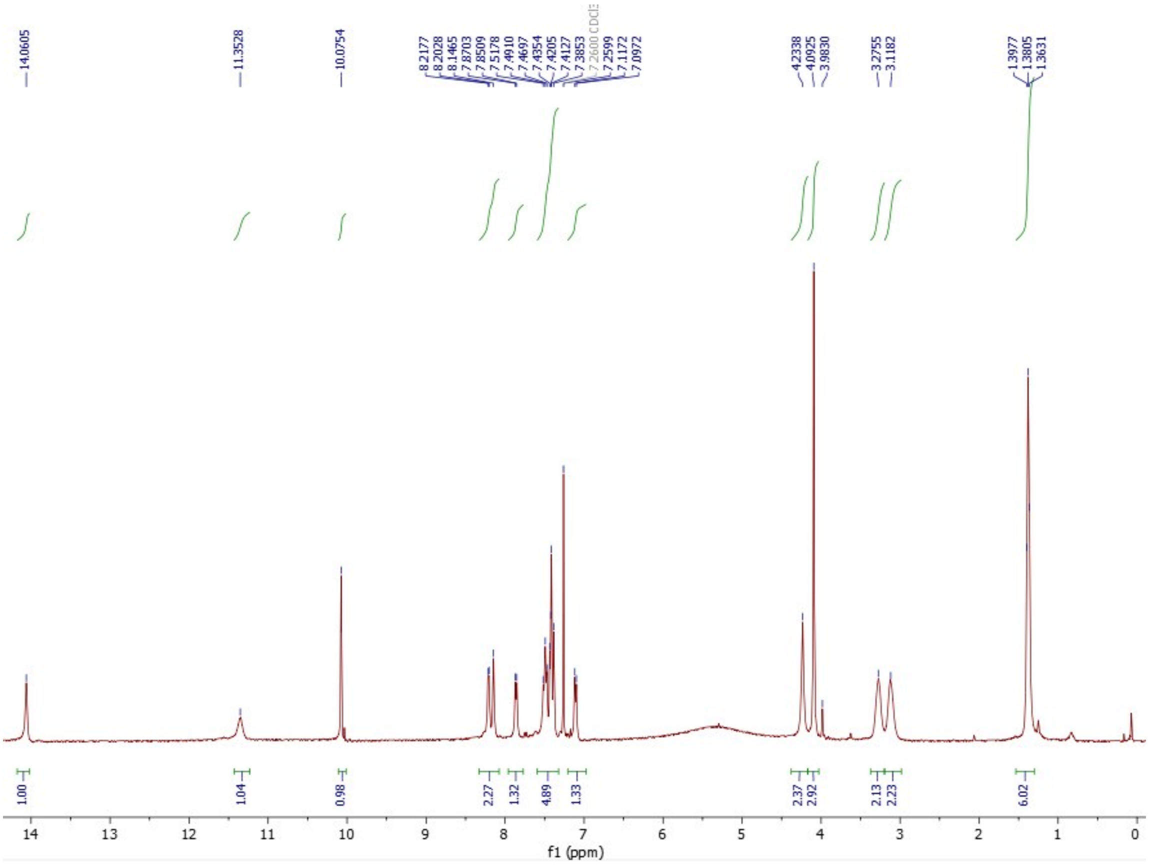

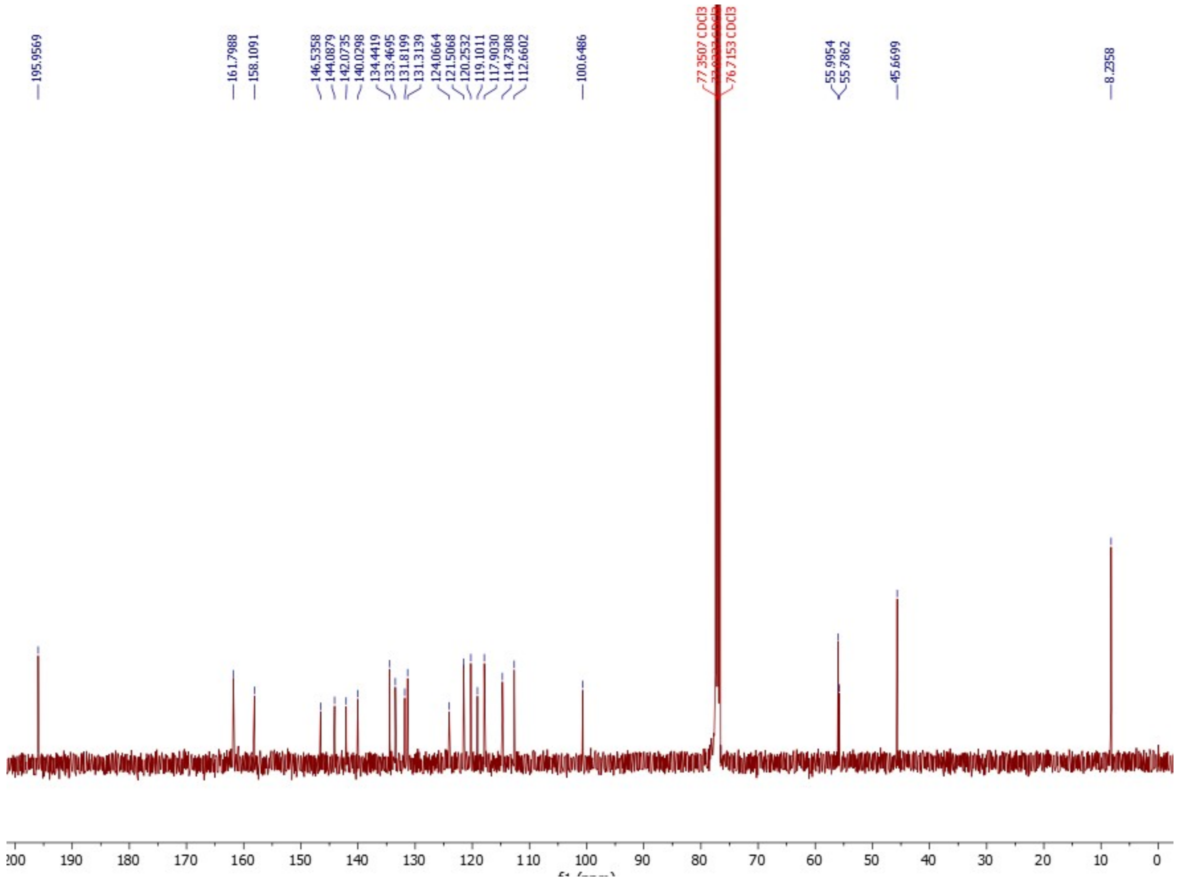

^{1}H and [13]C Spectra ($CDCl_3$) for compound 4

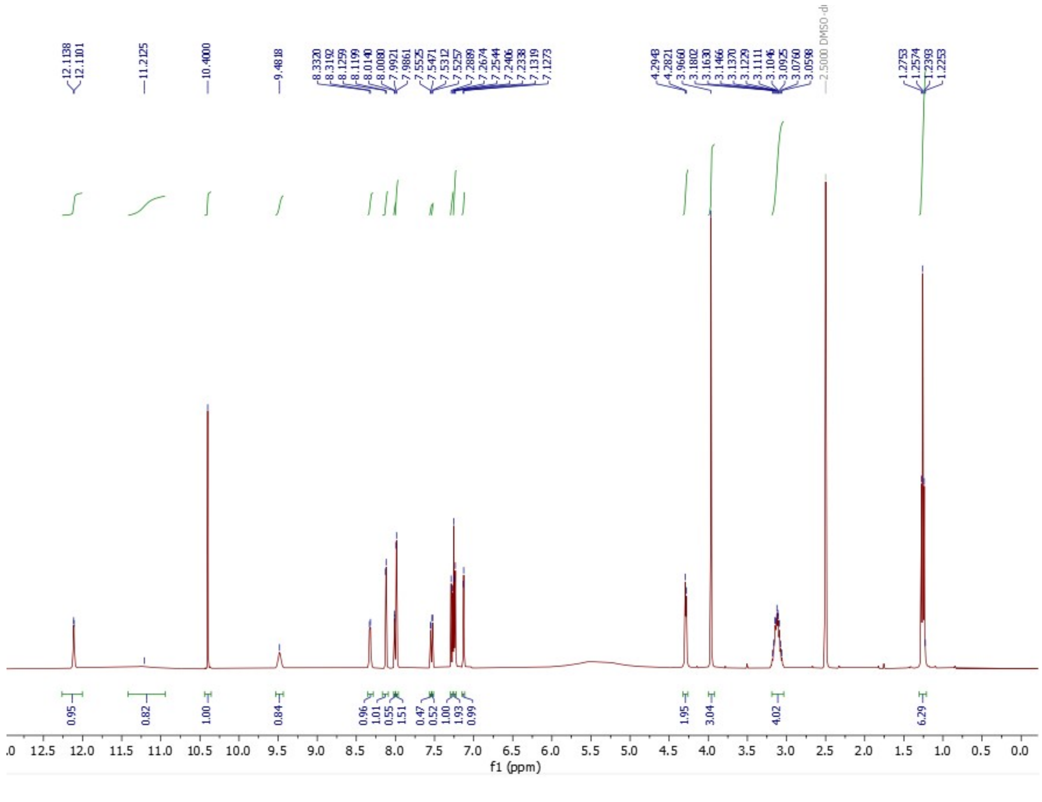

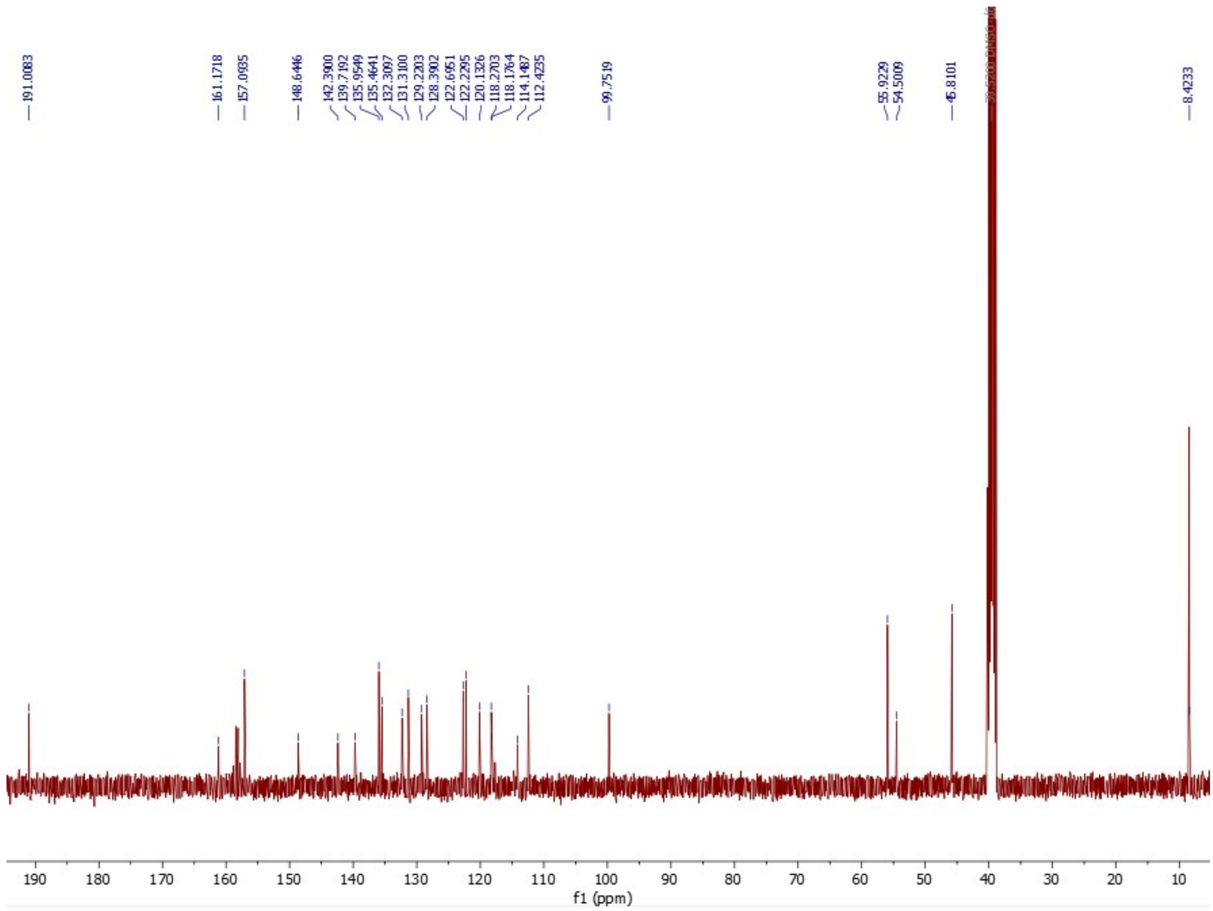

^{1}H and ^{13}C Spectra ($CDCl_3$) for compound 8

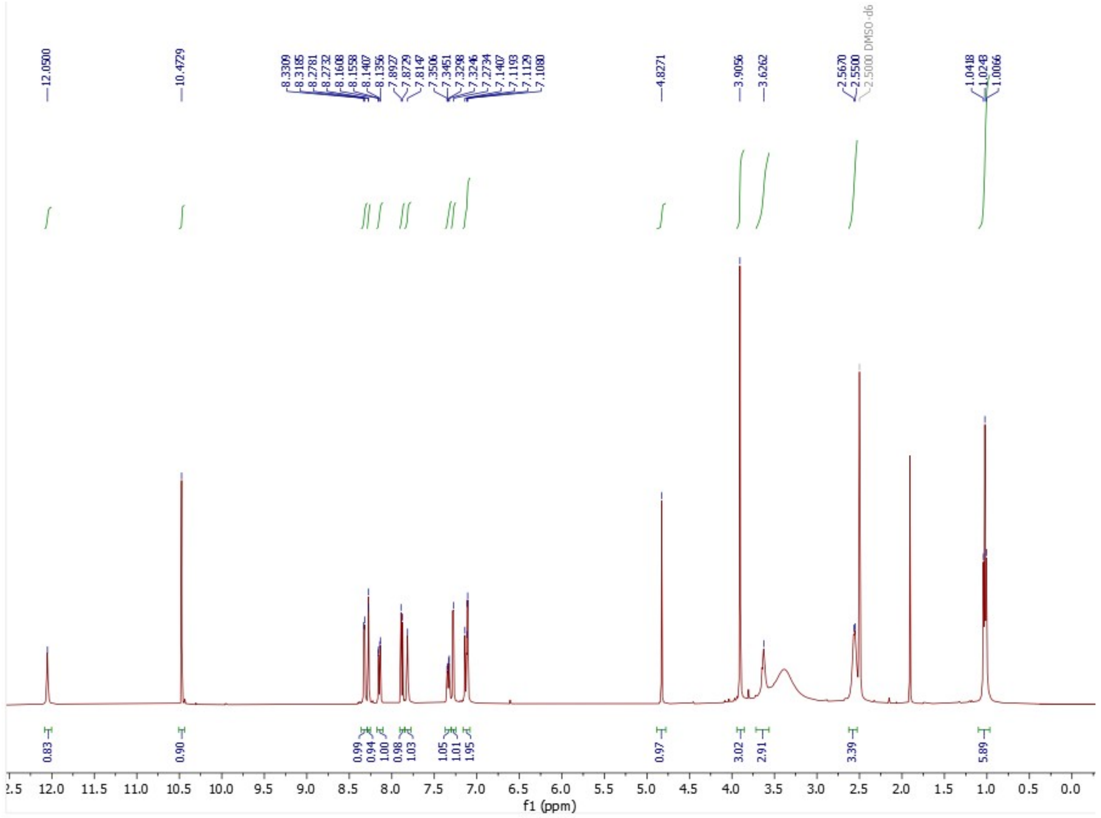

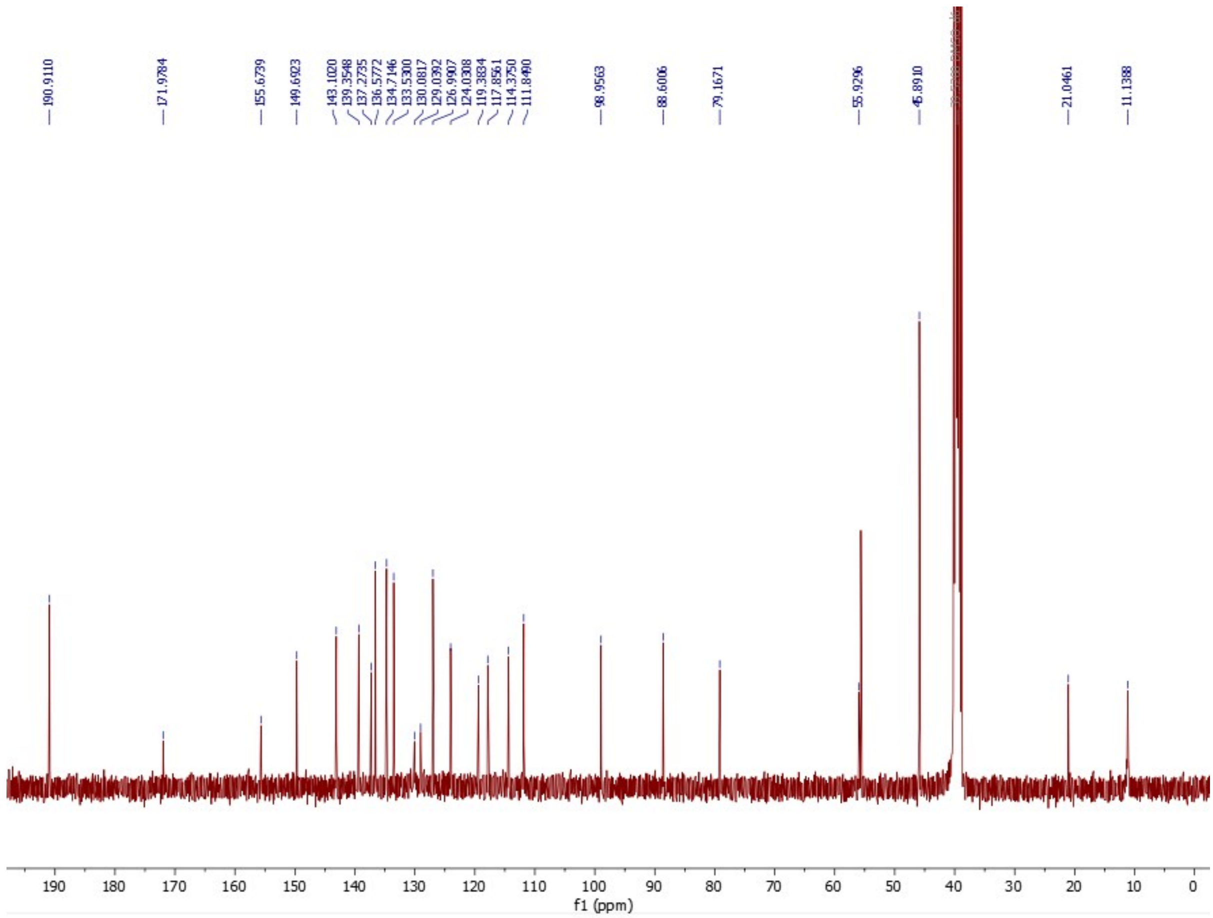

^{1}H and ^{13}C Spectra ($CDCl_3$) for compound 31

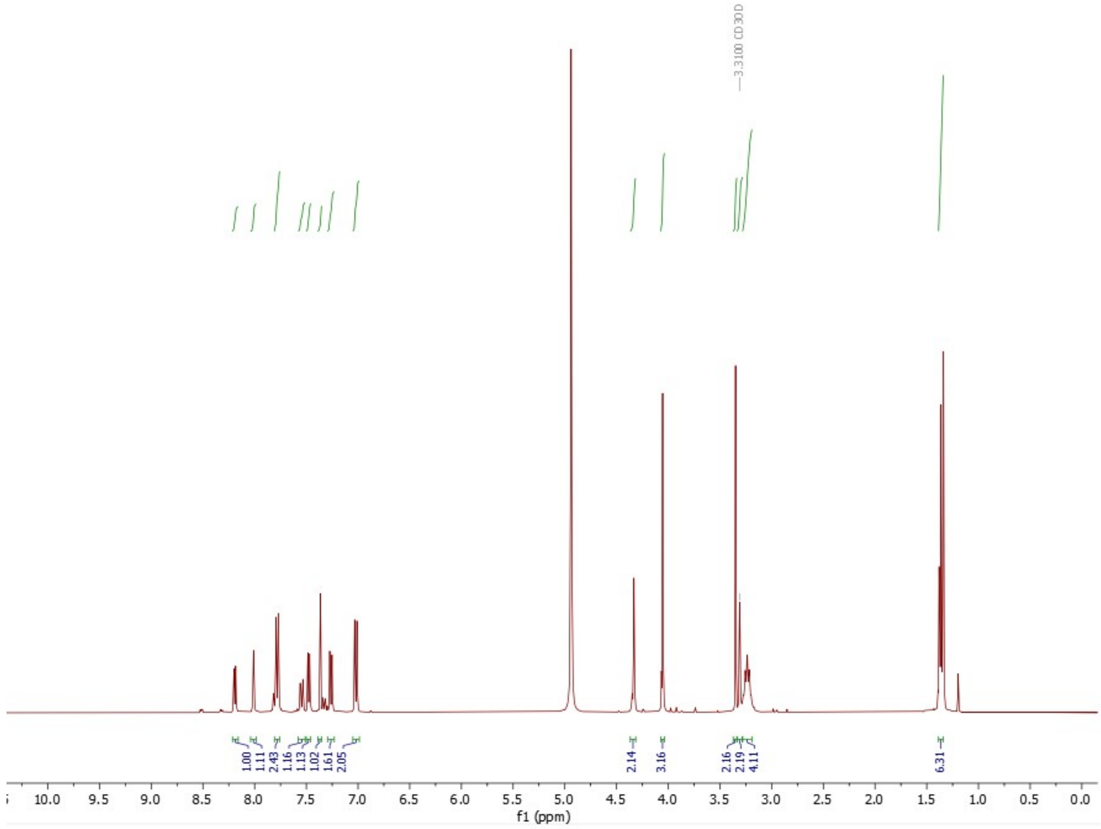

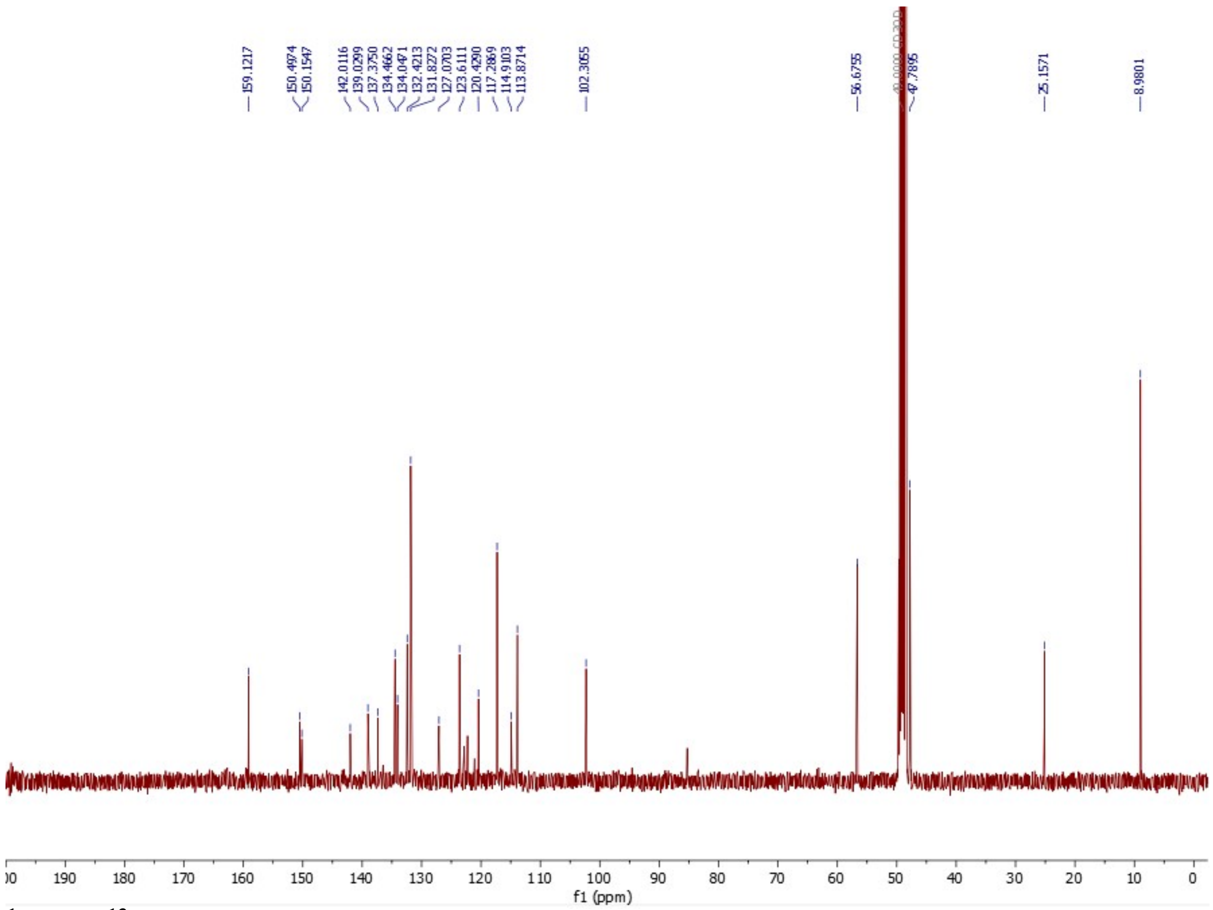

^{1}H and ^{13}C Spectra ($CDCl_3$) for compound 12

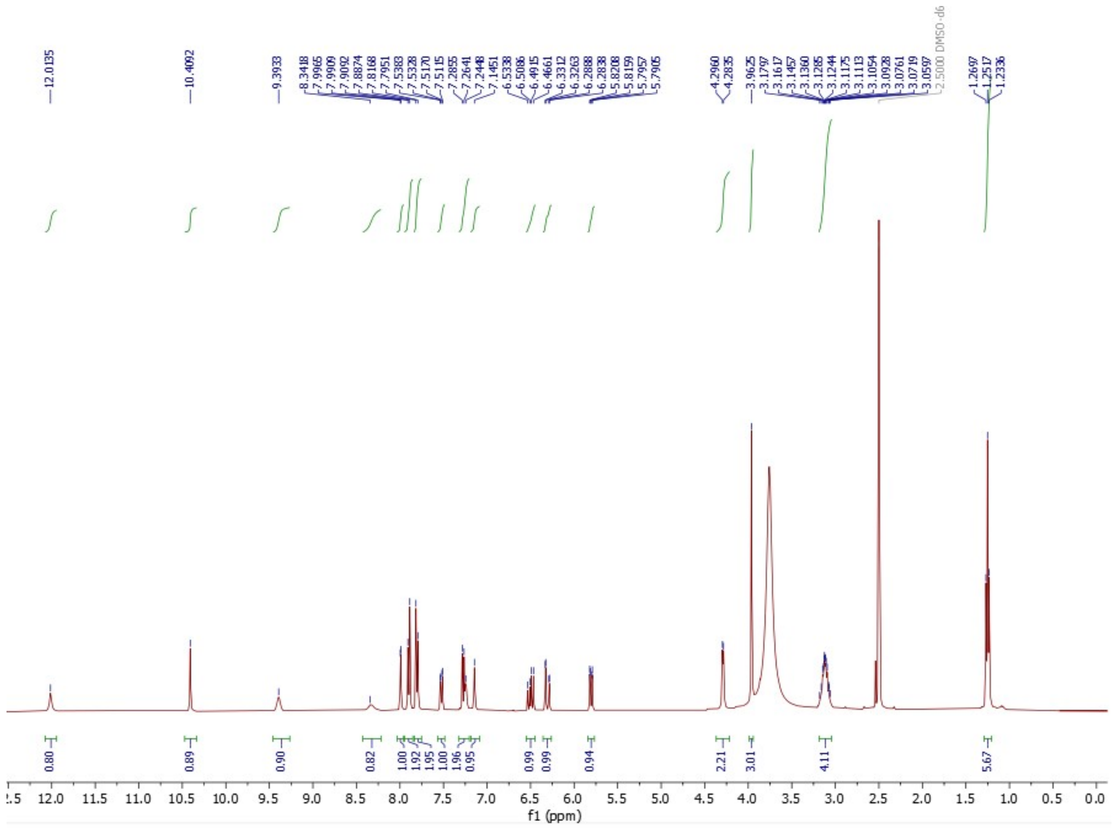

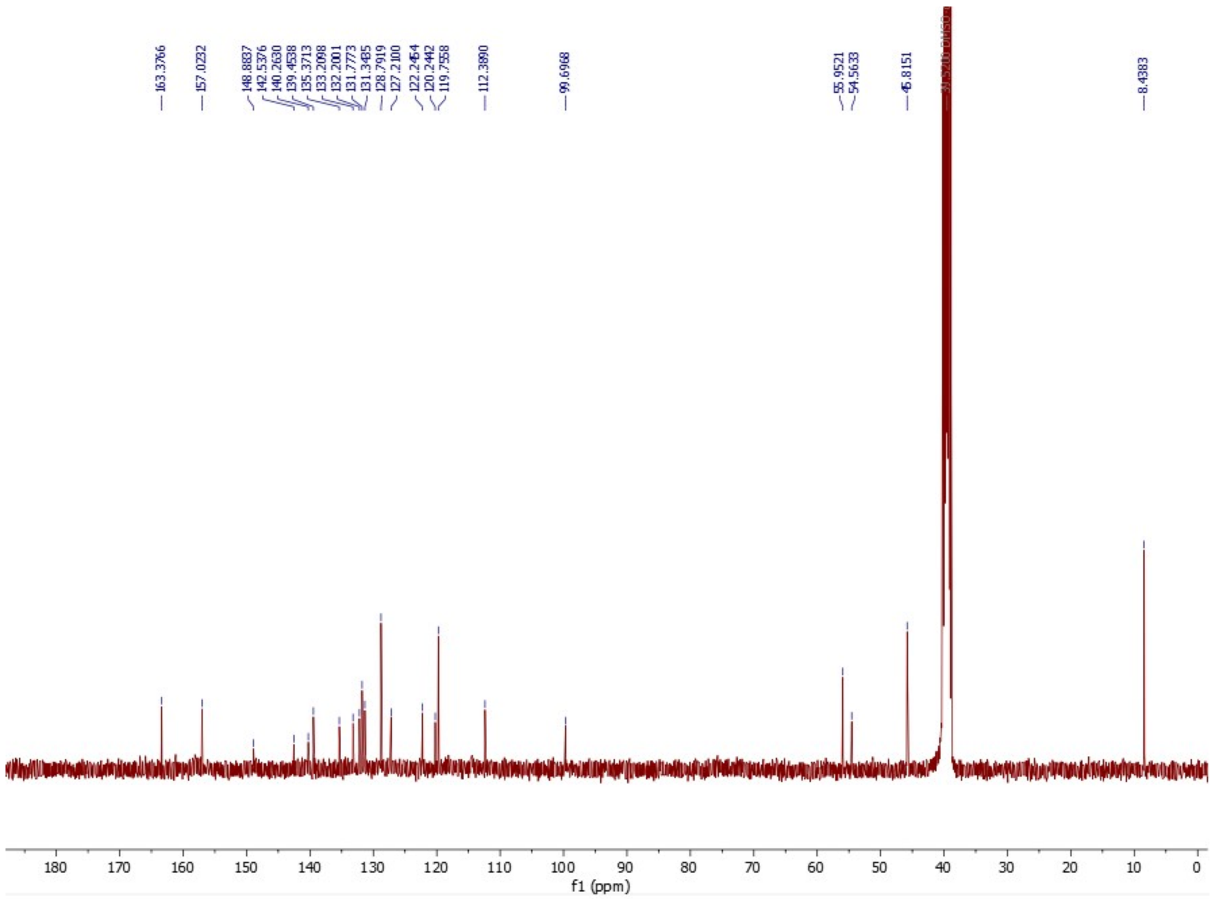

^{1}H and ^{13}C Spectra ($CDCl_3$) for compound 38

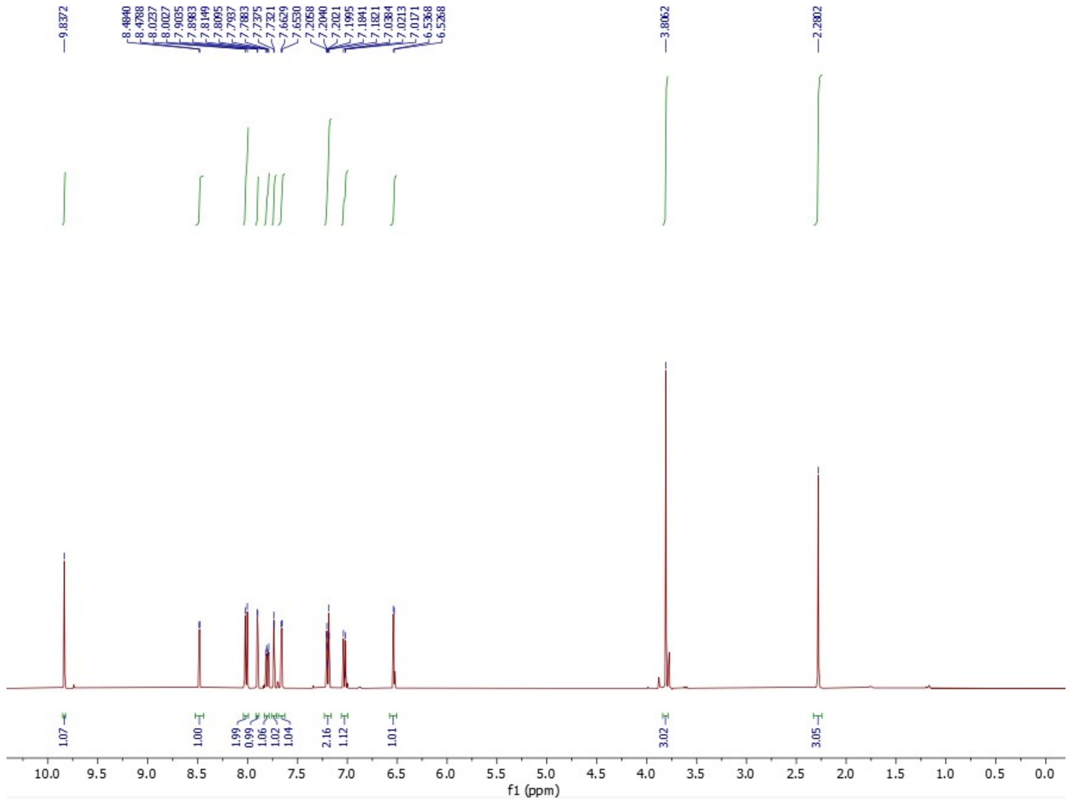

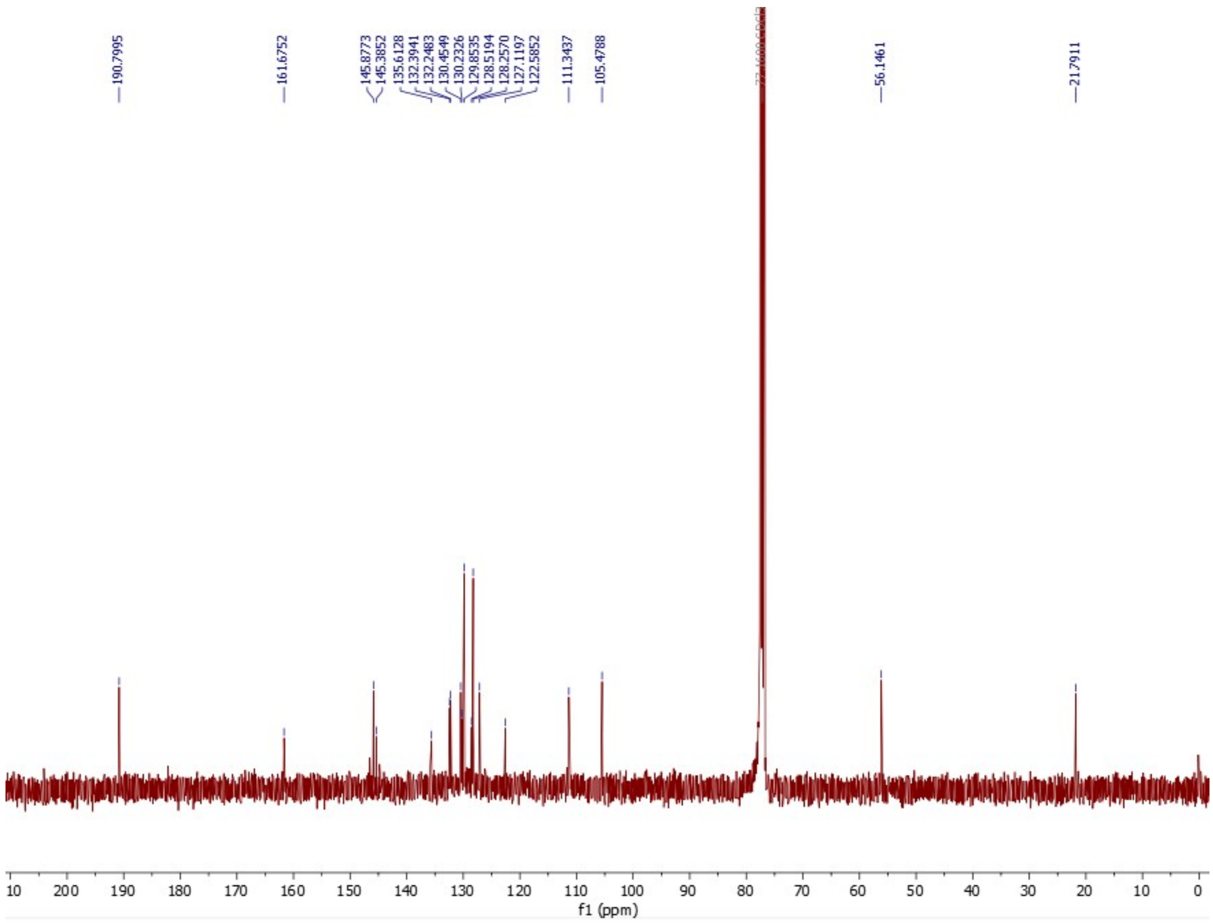

^{1}H and ^{13}C Spectra ($CDCl_3$) for compound 13

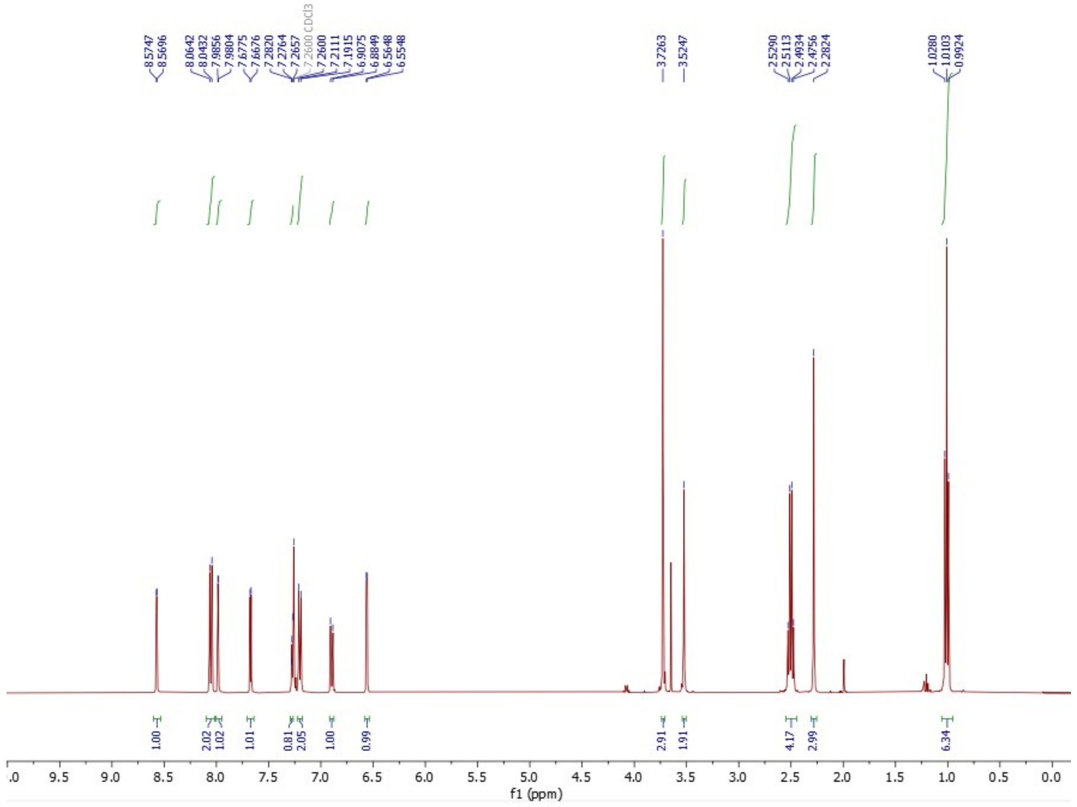

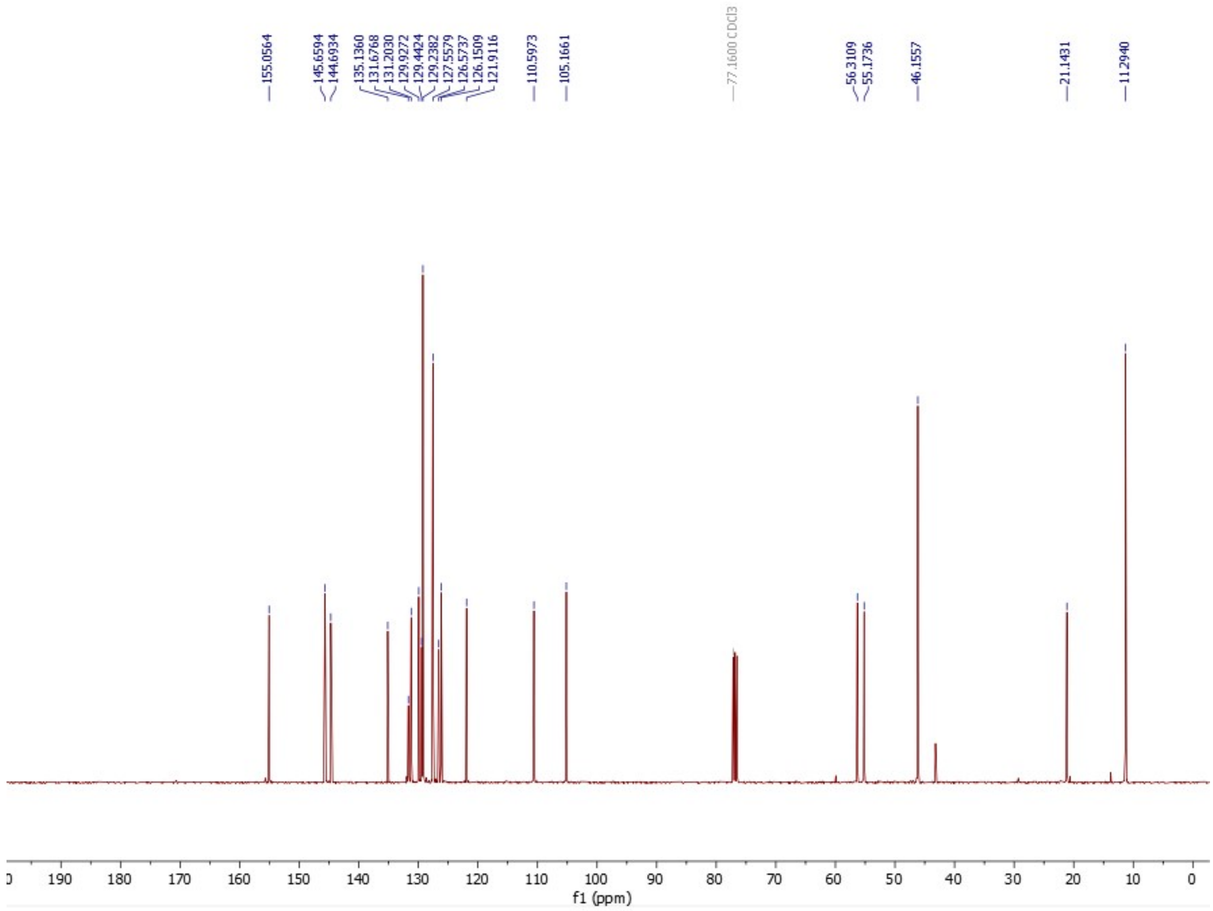

^{1}H and [13]C Spectra ($CDCl_3$) for compound 37

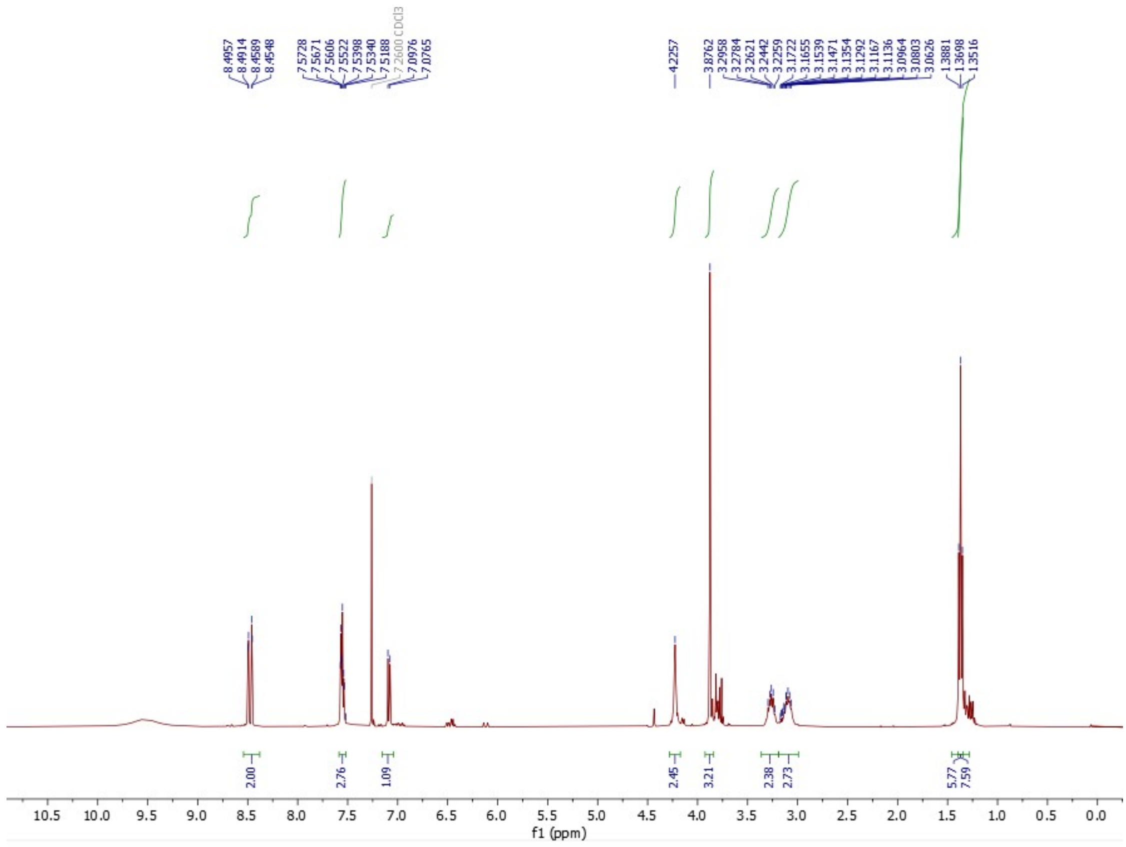

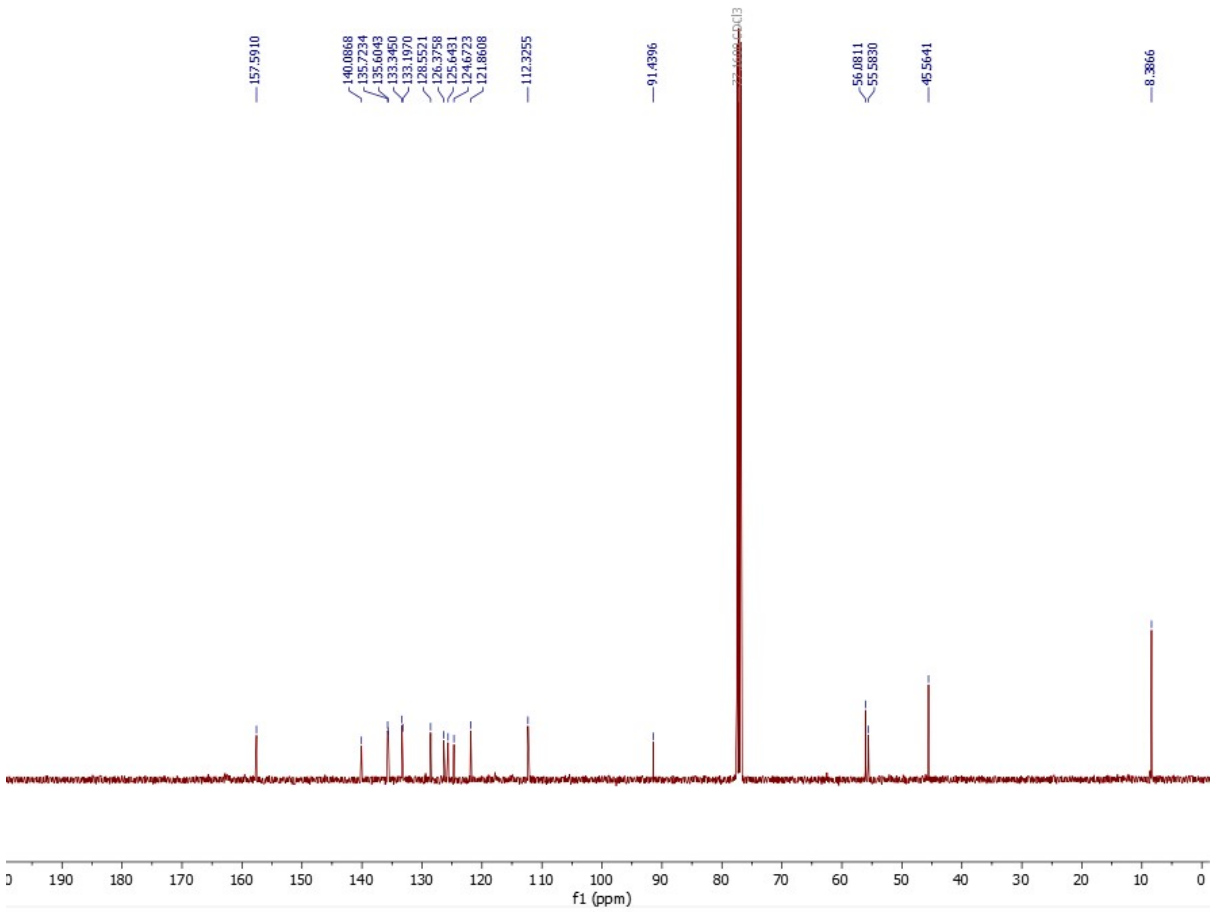

^1H and ^{13}C Spectra ($CDCl_3$) for compound 3

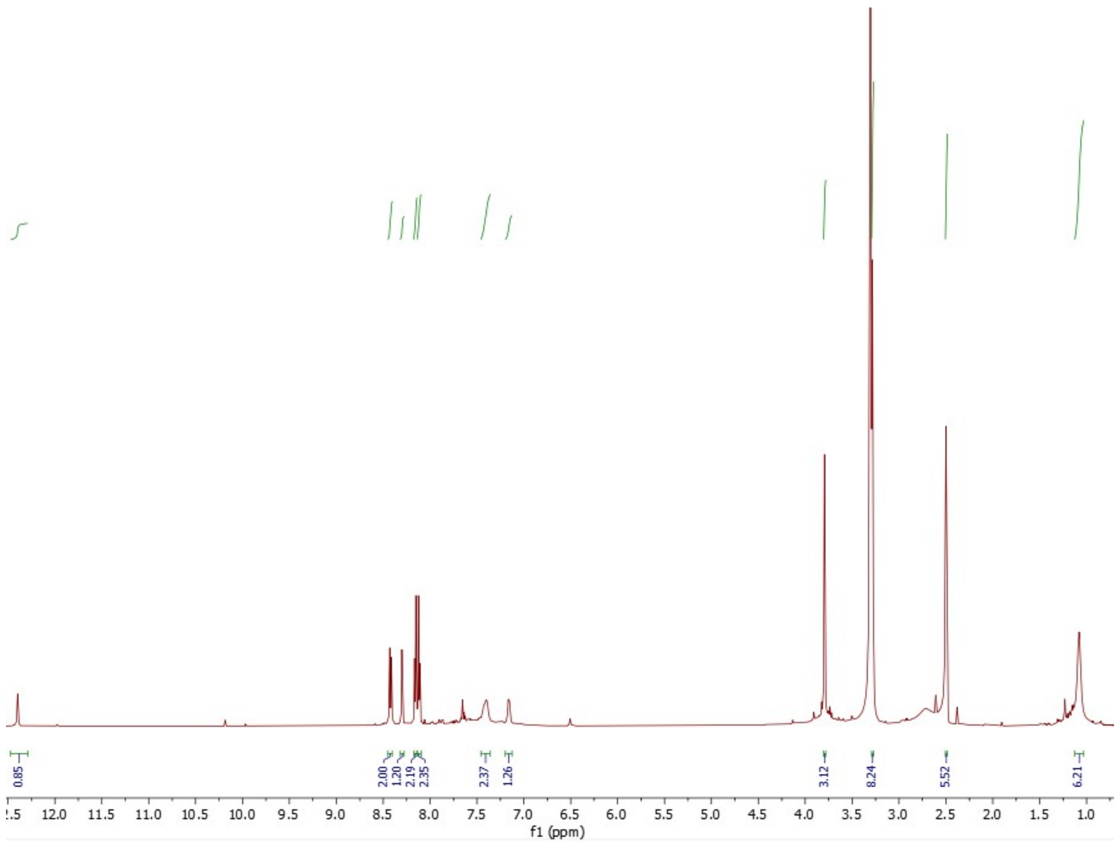

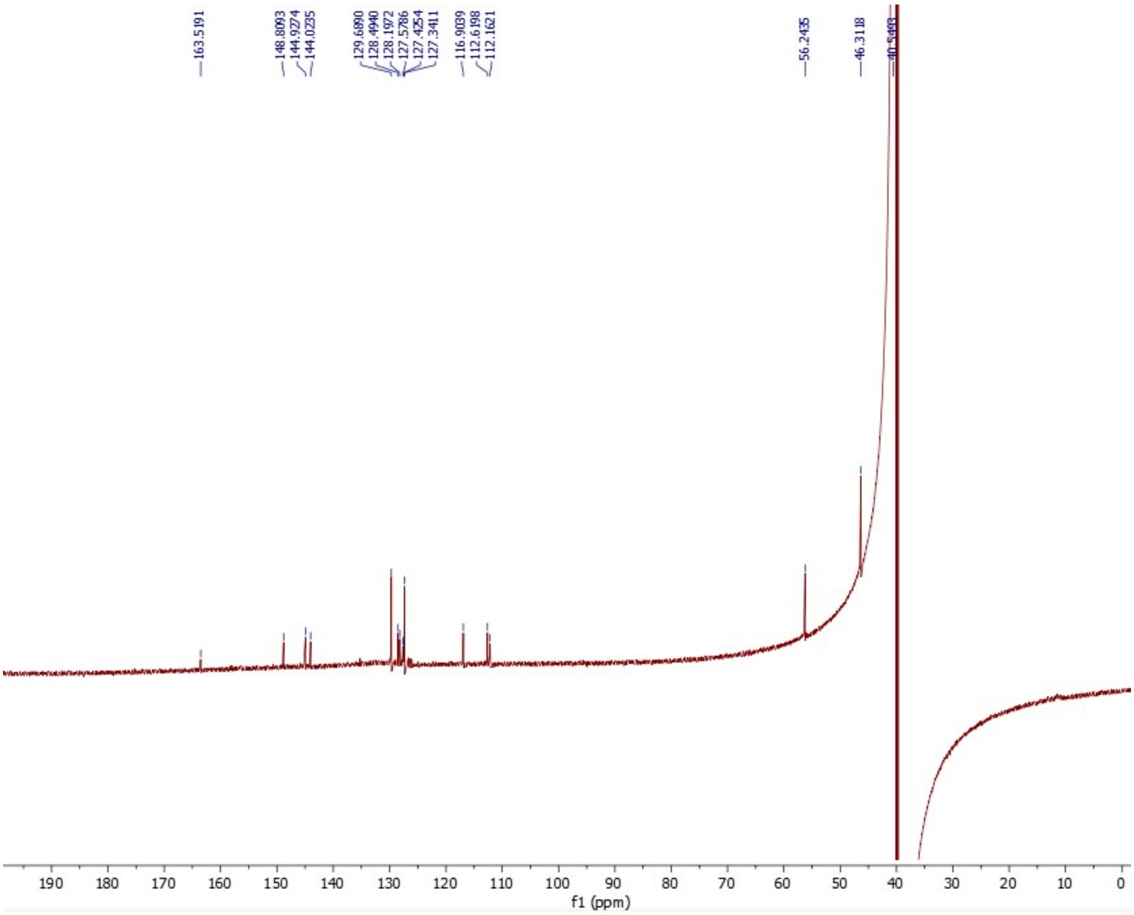

^{1}H and ^{13}C Spectra ($CDCl_3$) for compound 7

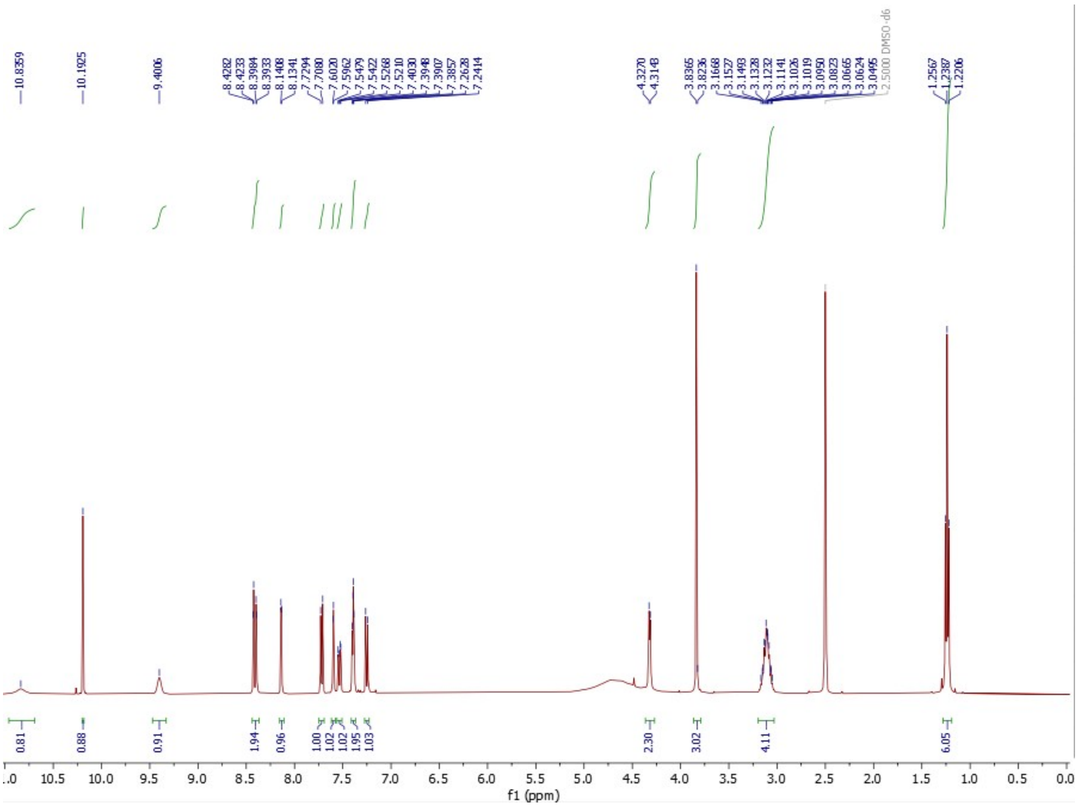

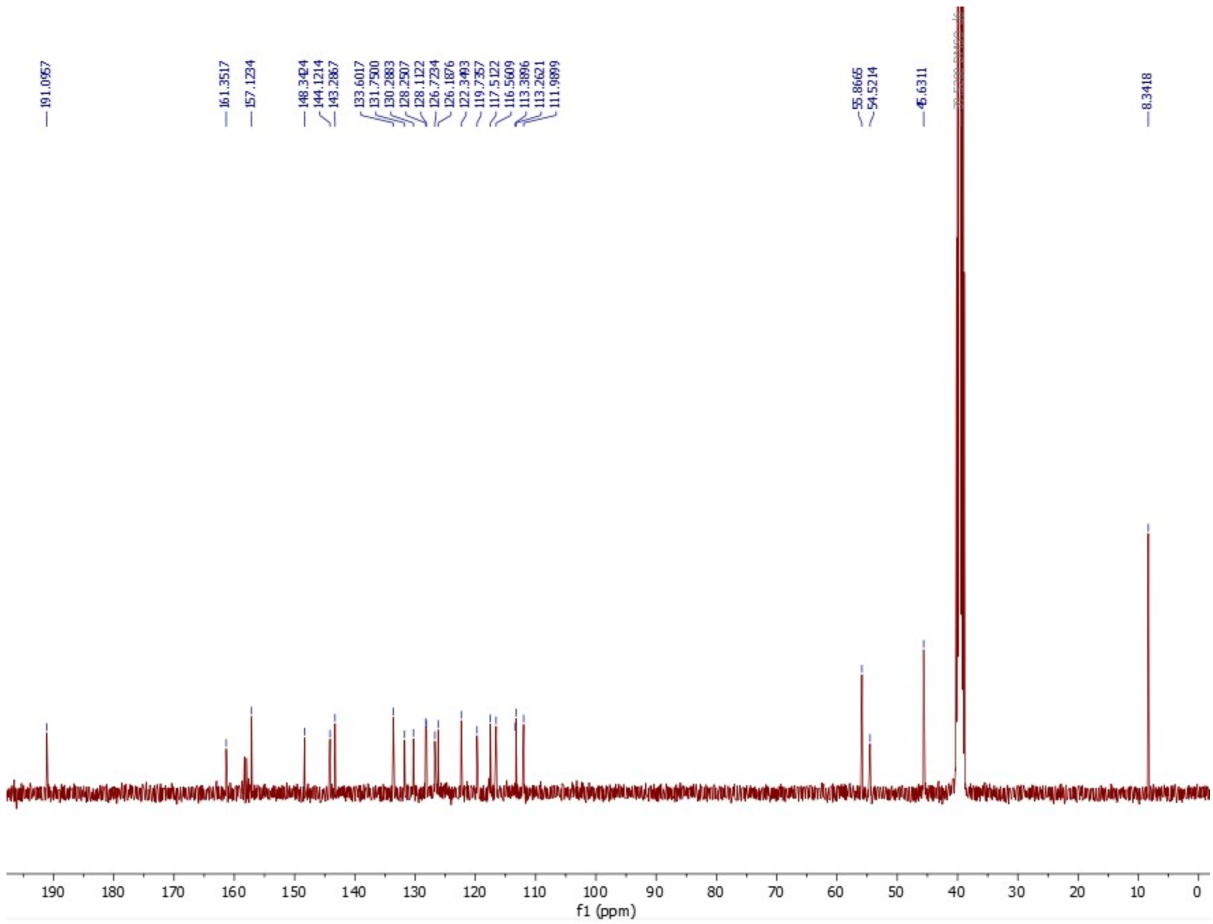

^{1}H and [13]C Spectra ($CDCl_3$) for compound 5

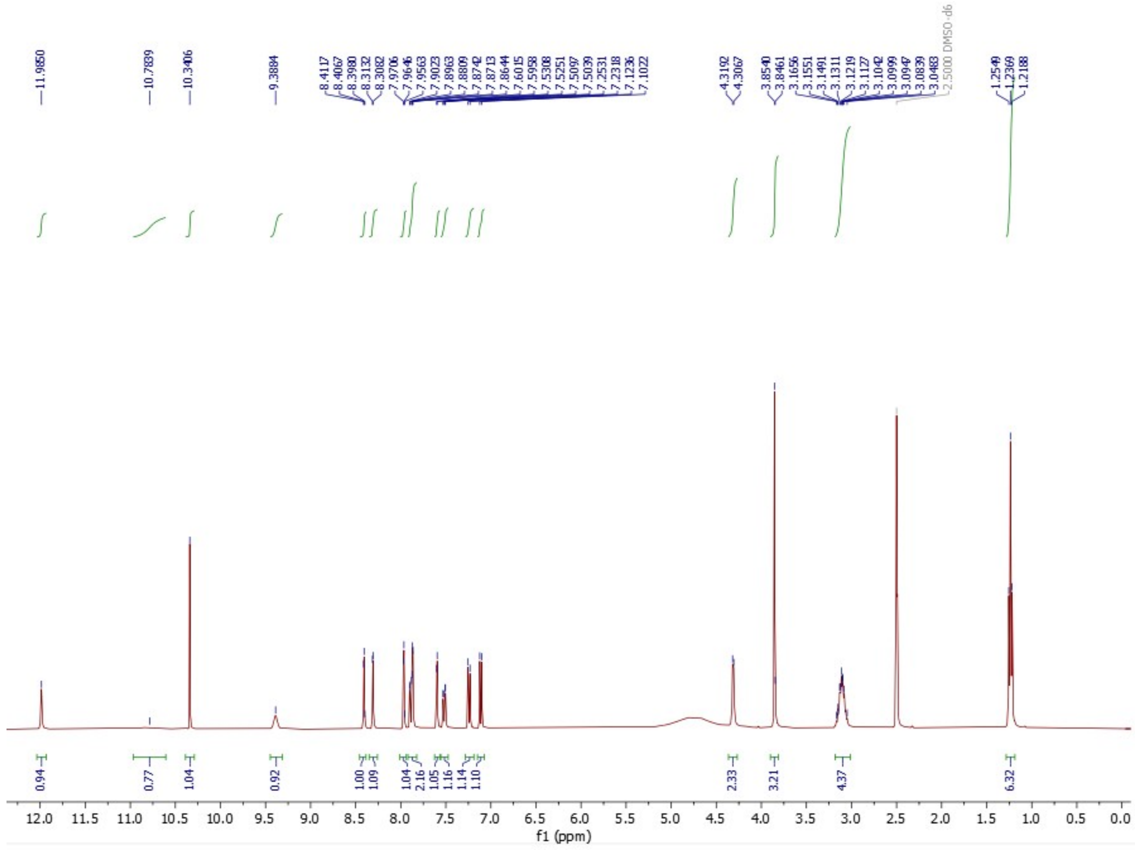

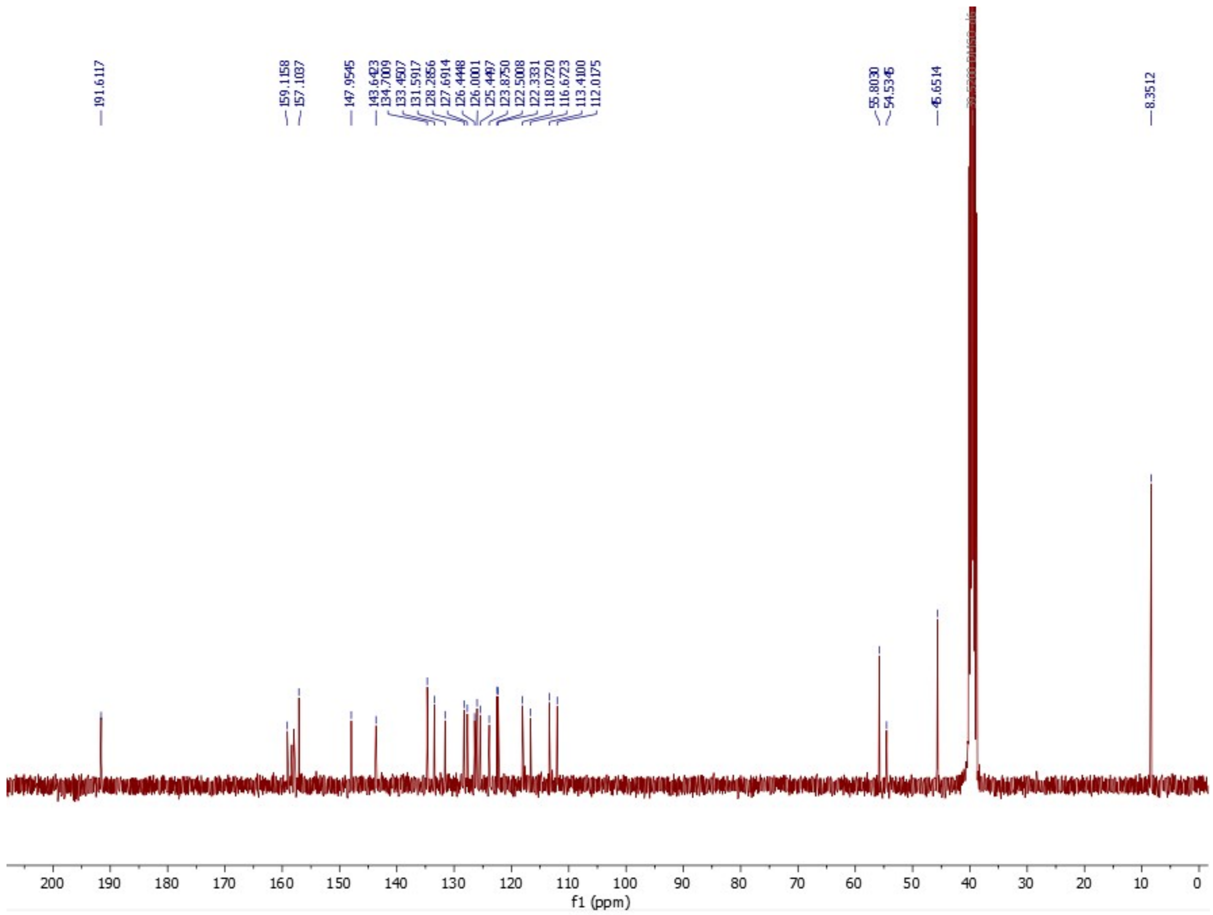

^{1}H and ^{13}C Spectra ($CDCl_3$) for compound 9

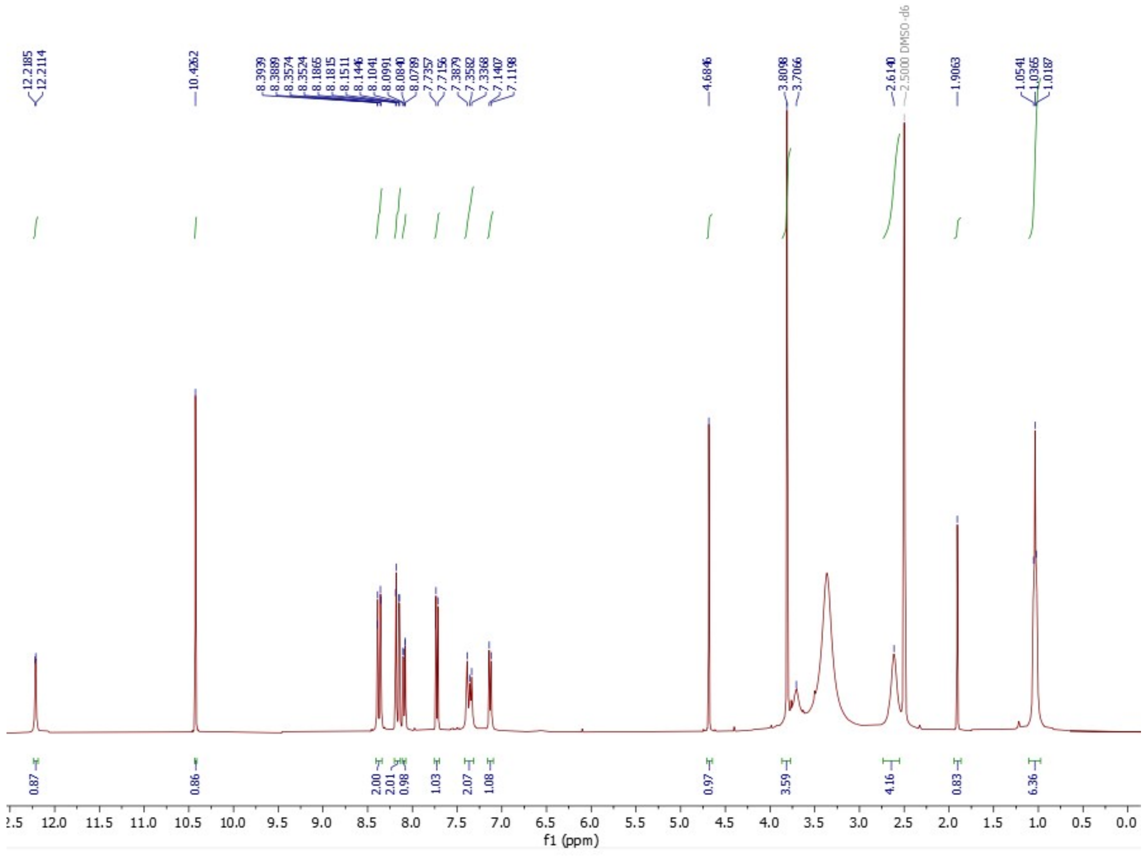

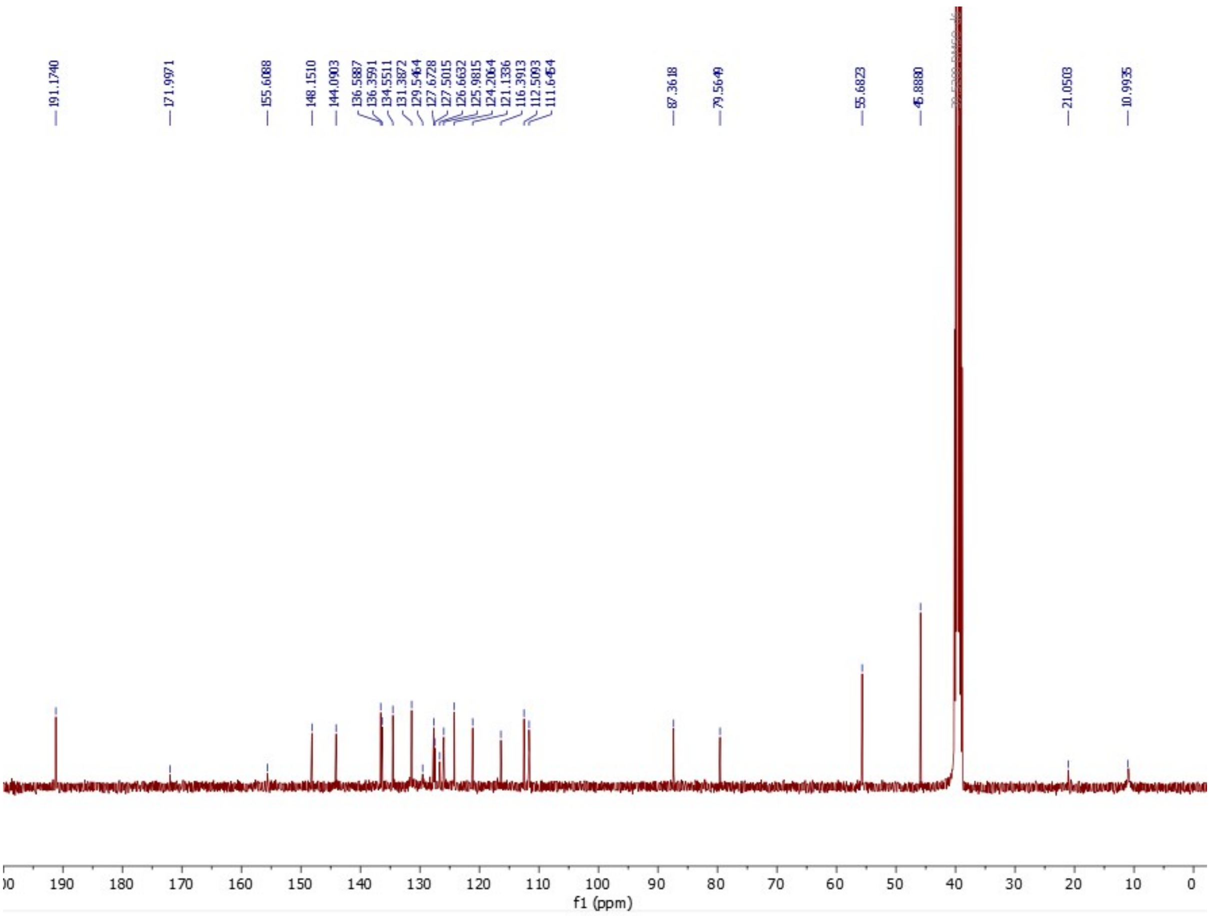

^{1}H and [13]C Spectra ($CDCl_3$) for compound 38

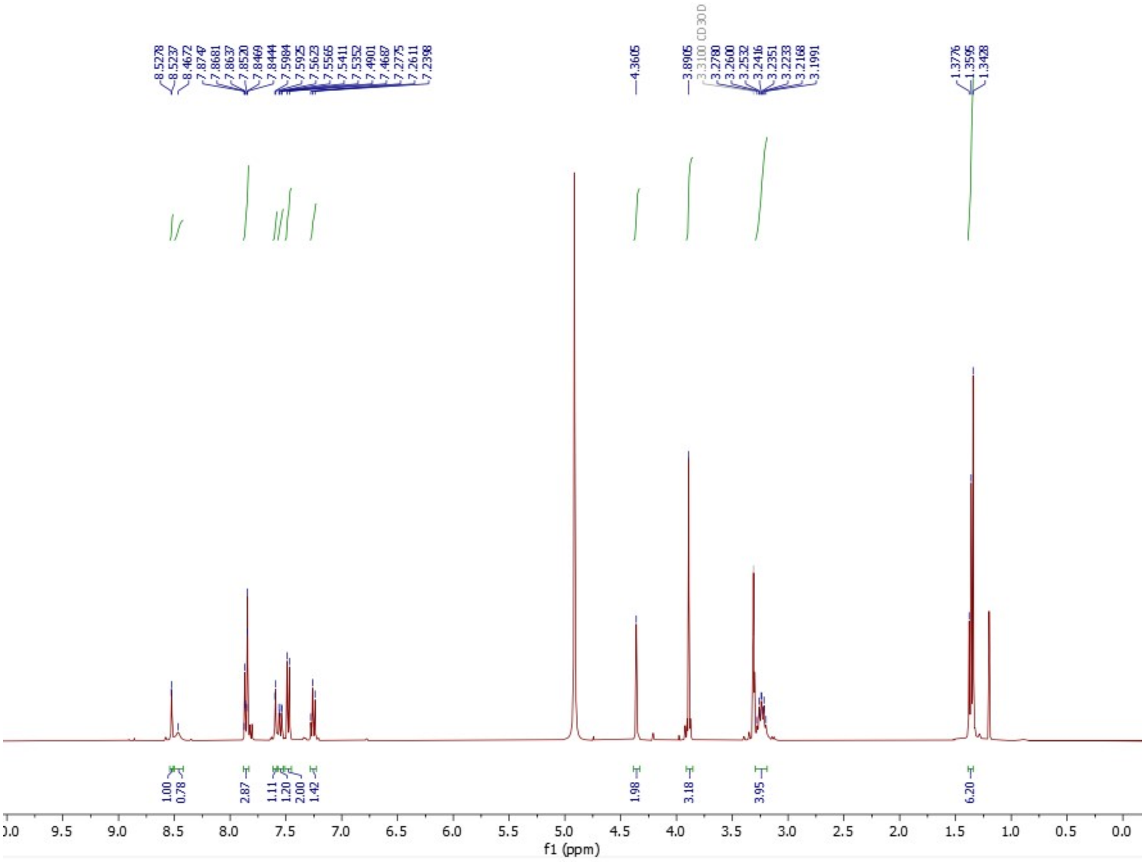

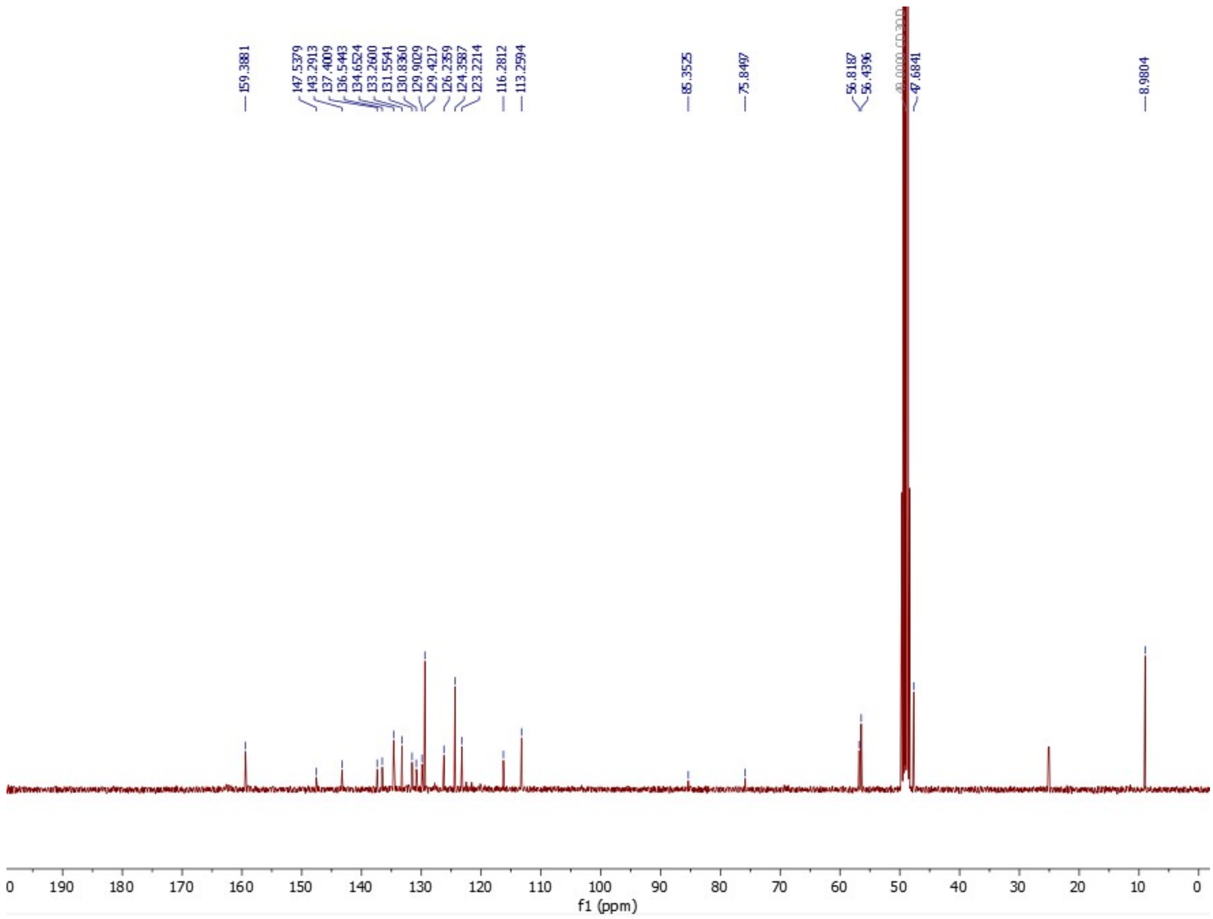

^{1}H and ^{13}C Spectra ($CDCl_3$) for compound 13

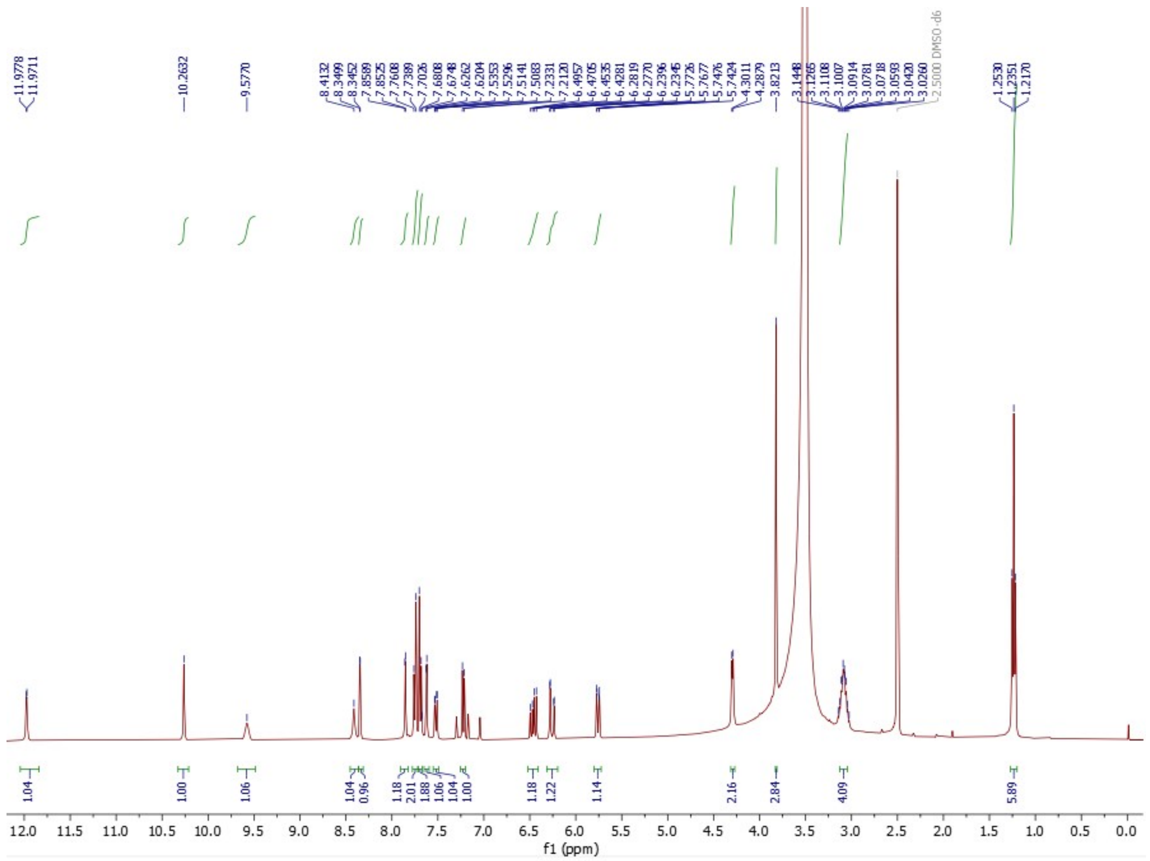

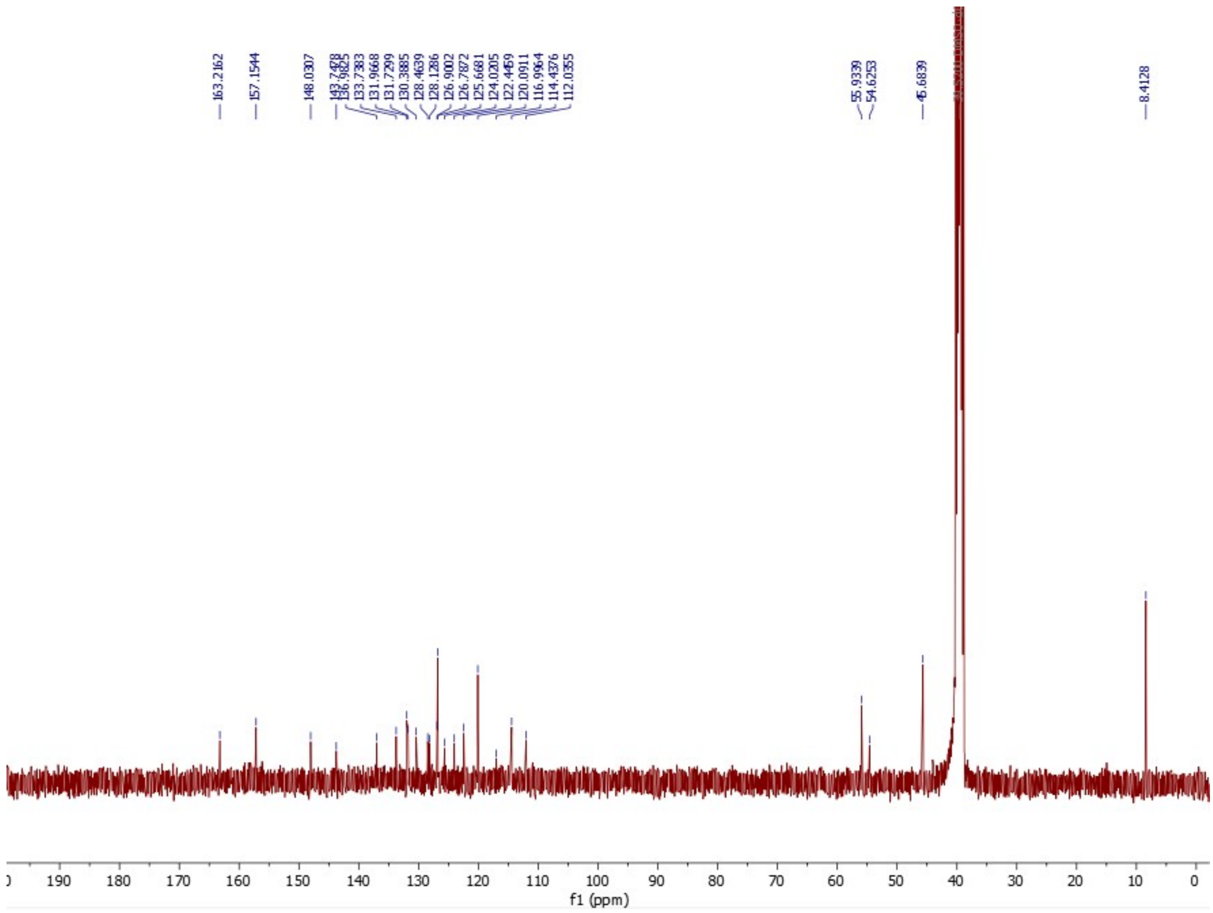

HPLC data for all tested compounds

Compound 2

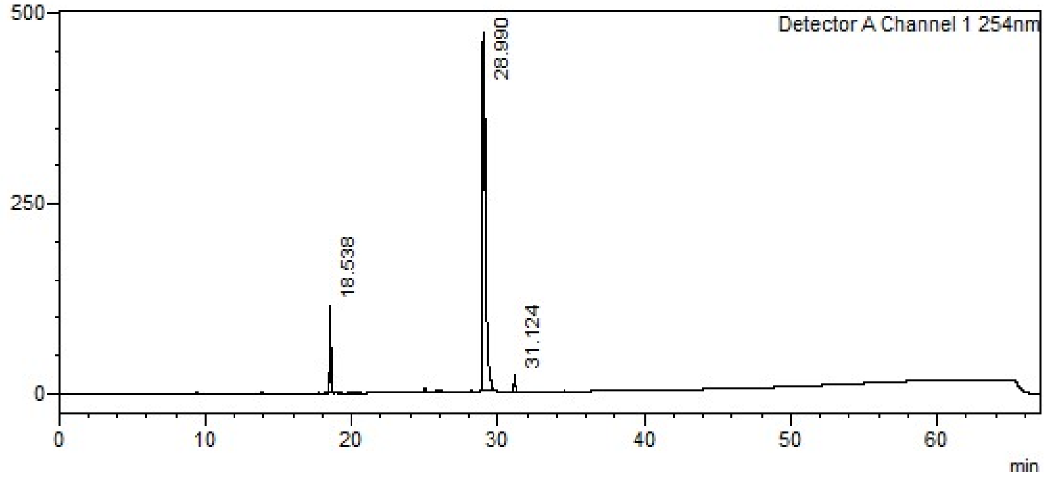

Compound 3

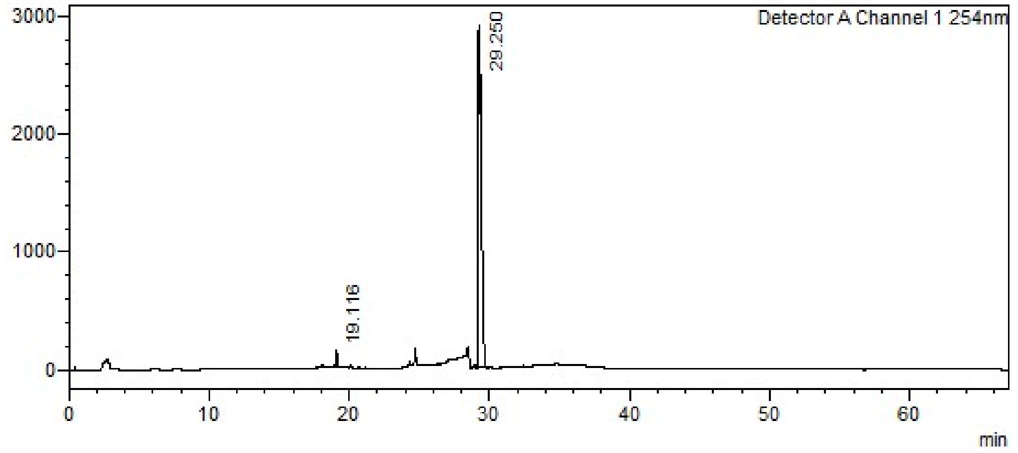

Compound 4

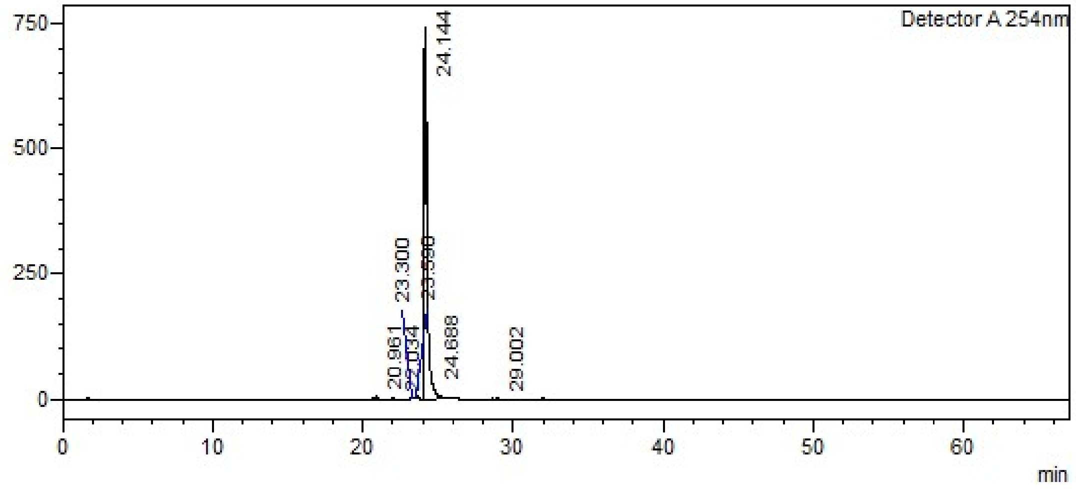

Compound 5

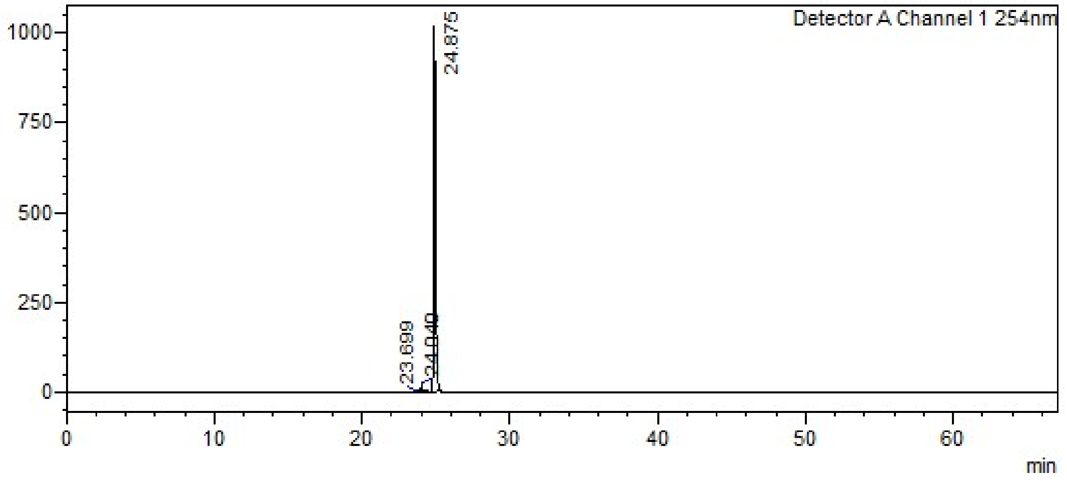

Compound 6

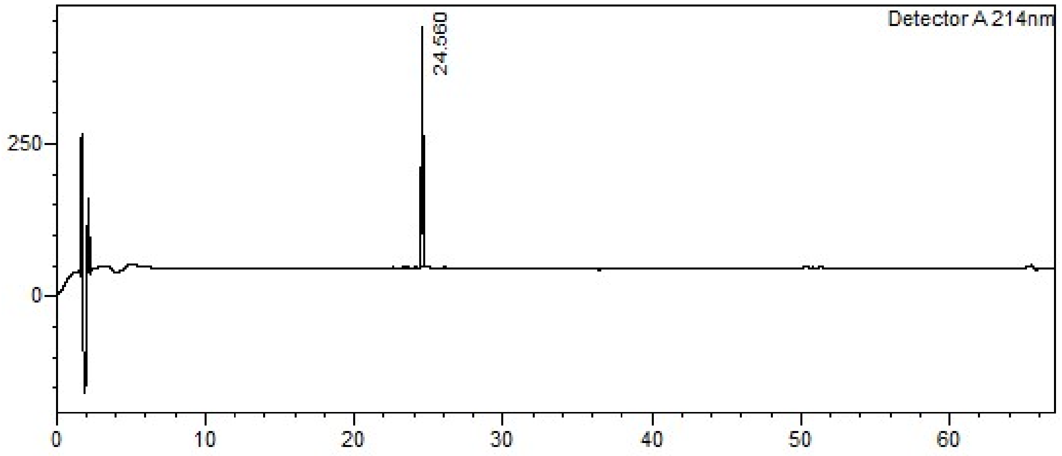

Compound 7

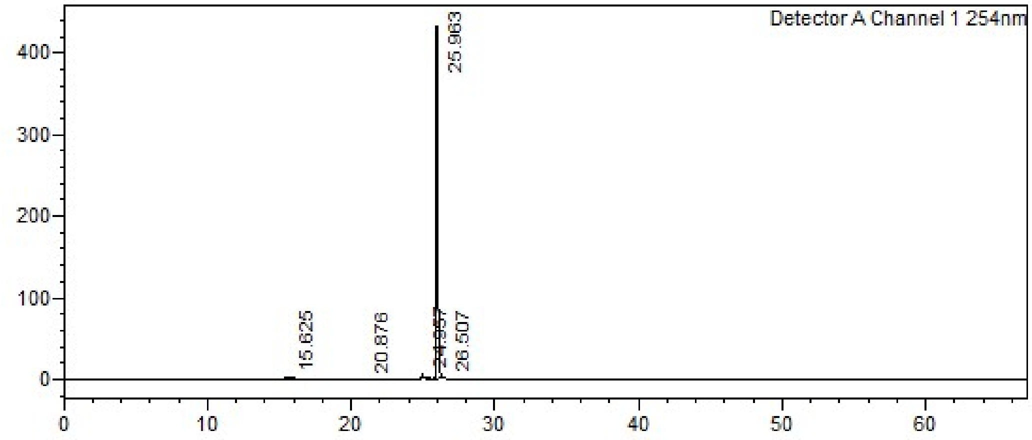

Compound 8

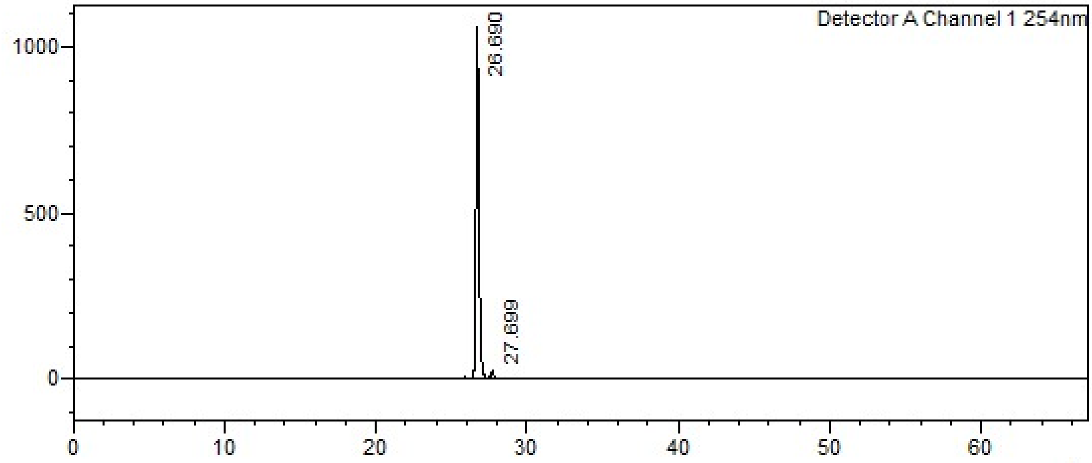

Compound 9

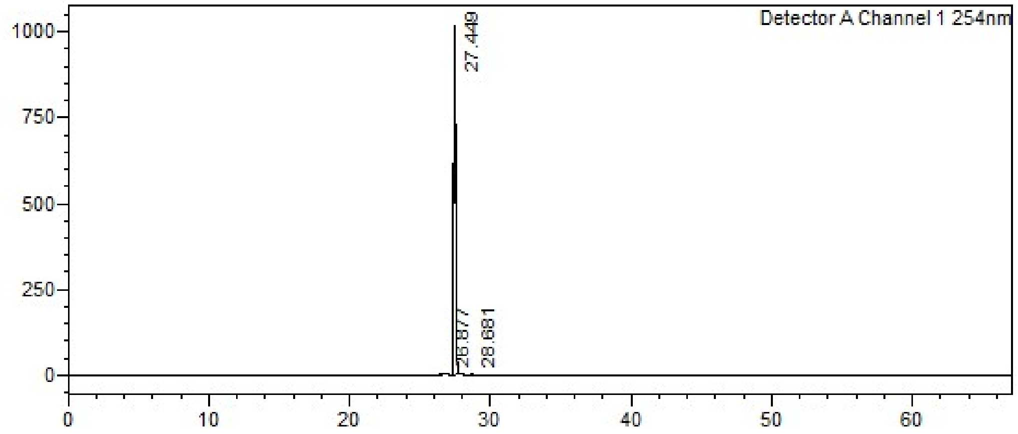

Compound 12

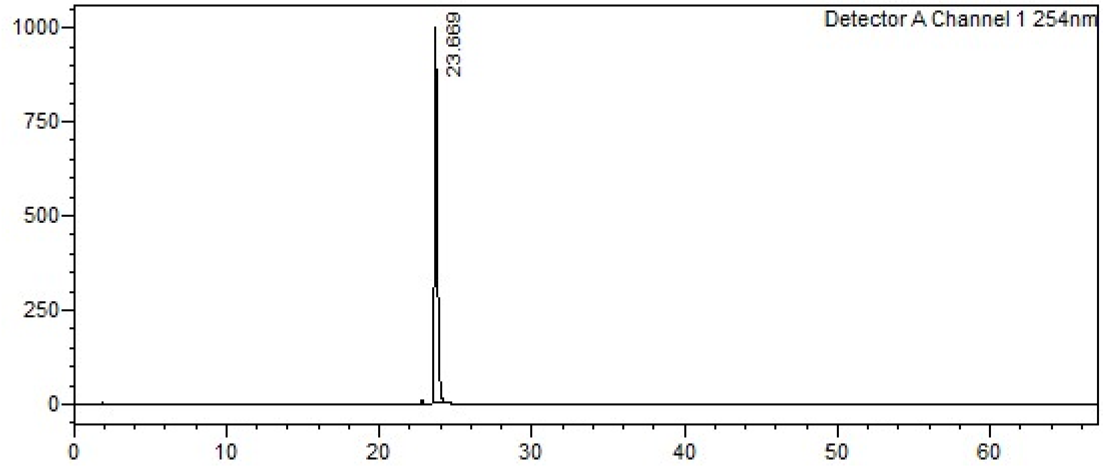

Compound 13

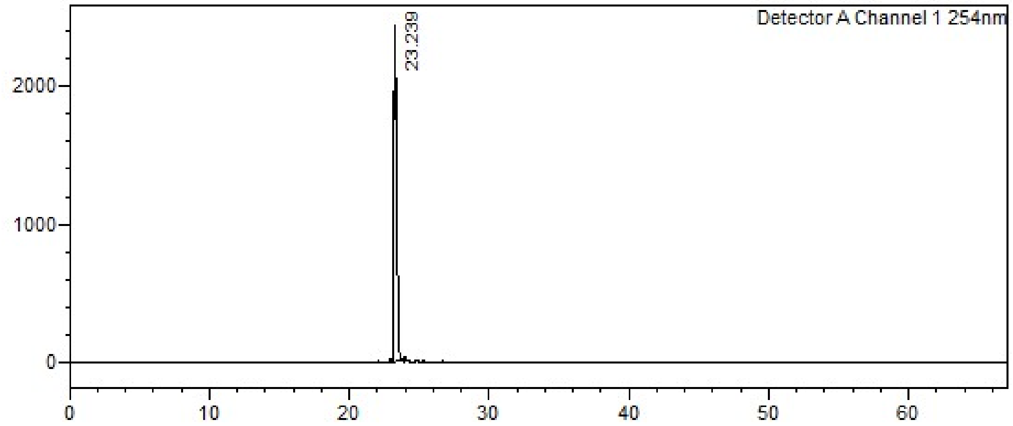

References

1. Lou, T. S.-B.; Willis, M. C. Arylsulfonyl fluoride boronic acids: Preparation and coupling reactivity. *Tetrahedron* **2020**, *76* (I).
2. Quach, D.; Tang, G.; Anantharajan, J.; Baburajendran, N.; Poulsen, A.; Wee, J. L. K.; Retna, P.; Li, R.; Liu, B.; Tee, D. H. Y.; et al. Strategic Design of Catalytic Lysine-Targeting Reversible Covalent BCR-ABL Inhibitors. *Angew. Chem. Int. Ed* **2021**, *60* (31), 17131-17137
3. Chen, P.; Tang, G.; Zhu, C.; Sun, J.; Wang, X.; Xiang, M.; Huang, H.; Wang, W.; Li, L.; Zhang, Z.-M.; et al. 2-Ethynylbenzaldehyde-Based, Lysine-Targeting Irreversible Covalent Inhibitors for Protein Kinases and Nonkinases. *JACS* **2023**, Article; Early Access.
4. Mahindra, A.; Janha, O.; Mapesa, K.; Sanchez-Azqueta, A.; Alam, M. M.; Amambua-Ngwa, A.; Nwakanma, D. C.; Tobin, A. B.; Jamieson, A. G. Development of Potent PfCLK3 Inhibitors Based on TCMDC-135051 as a New Class of Antimalarials. *J. Med. Chem.* **2020**, *63* (17), 9300-9315.
5. Yongjin Xu, Chunxia Gao, Joakim Andréasson, and Morten Grøtli. Synthesis and Photophysical Characterization of Azoheteroarenes. *Org. Lett.* **2018** 20 (16), 4875-4879
