# Supplementary material for: Lysine targeting covalent inhibitors of malarial kinase PfCLK3
Source: RSC Med Chem. 2025 May 27;16(8):3530–40. doi: 10.1039/d5md00335k (PMC12164071; doi:10.1039/d5md00335k)
Supplement: MD-016-D5MD00335K-s004 [file MD-016-D5MD00335K-s004.pdf]

Supplementary Information (SI) for RSC Medicinal Chemistry.
This journal is © The Royal Society of Chemistry 2025

Trypsin Digest Results obtained from Compound 4

23 unmodified peptides (46.7% sequence coverage)

2 Possible modified Peptides (∆mass 413.2103 Da).

Modification of Peptide 57 to 65 was confirmed by MSMS (Data not shown).

m/z Submitted	MH^+ Matched	Delta ppm	Start	End	Missed Cleavages	Sequence
814.3842	814.3838	0.517	245	250	0	(K)LMMEYK(G)
843.4148	843.4141	0.785	238	244	0	(K)SNNHMIK(L)
847.4314	847.4308	0.668	357	363	0	(R)ESIHFSK(S)
860.5359	860.5352	0.791	143	149	0	(K)QLFIALR(H)
1015.5945	1015.5935	0.943	202	210	0	(R)APEIILGFR(Y)
1028.6109	1028.6099	0.962	290	298	0	(R)VISDLRPTK(N)
1117.6045	1117.6034	1.02	22	31	1	(K)AMVGEVIDKR(Y)
1121.5453	1121.5442	0.976	63	71	1	(K)VIRDNDMMK(K)
1156.6948	1156.6936	1.06	325	334	1	(K)IKQLGDLLEK(C)
1415.6097	1415.6074	1.62	364	375	0	(K)SQNEGSENLYFQ(-)
1421.7514	1421.7496	1.30	83	93	3	(K)KLNQYDKDNKR(H)
1421.7514	1421.7496	1.30	83	93	3	(K)KLNQYDKDNKR(H)
1481.8324	1481.8263	4.09	199	210	1	(R)FYRAPEIILGFR(Y)
1601.8095	1601.8071	1.51	344	356	0	(R)YTPDQALQHPYLR(E)
1601.8095	1601.8071	1.51	344	356	0	(R)YTPDQALQHPYLR(E)
1640.0003	1639.9854	9.08	285	298	2	(K)KEVVRVISDLRPTK(N)
1864.0027	1863.9997	1.62	156	171	0	(R)IMHADLKPDNILINEK(F)
1864.0027	1863.9997	1.62	156	171	0	(R)IMHADLKPDNILINEK(F)
2256.1227	2256.1183	1.94	211	231	0	(R)YDAQIDVWSAAATVFELATGK(I)
2256.1227	2256.1183	1.94	211	231	0	(R)YDAQIDVWSAAATVFELATGK(I)
2449.1252	2449.1207	1.81	260	279	0	(K)GGQFYSQHFNENLDFLYVDR(D)
2449.1252	2449.1207	1.81	260	279	0	(K)GGQFYSQHFNENLDFLYVDR(D)
3079.4024	3079.3969	1.79	260	284	1	(K)GGQFYSQHFNENLDFLYVDRDHYSK(K)
3079.4024	3079.3969	1.79	260	284	1	(K)GGQFYSQHFNENLDFLYVDRDHYSK(K)
**Possible Modified Peptides**						
1407.8890	1407.8868	1.54	57	65	1	(K)IPVAV**K**VIR(D)
2850.5426	2850.5366	2.12	156	176	1	(R)IMHADL**K**PDNILINE**K**FNALK(V)

Trypsin Digest Results obtained from Compound 9

9 unmodified peptides (21.1% sequence coverage)

2 Possible modified Peptides (Δmass 419.1998 Da).

m/z Submitted	MH⁺ Matched	Delta ppm	Start	End	Missed Cleavages	Sequence
915.5151	915.5146	0.586	327	334	0	(K)QLGDLLEK(C)
1015.5942	1015.5935	0.647	202	210	0	(R)APEIILGFR(Y)
1028.6107	1028.6099	0.796	290	298	0	(R)VISDLRPTK(N)
1117.6043	1117.6034	0.819	22	31	1	(K)AMVGEVIDKR(Y)
1601.8088	1601.8071	1.11	344	356	0	(R)YTPDQALQHPYLR(E)
1601.8088	1601.8071	1.11	344	356	0	(R)YTPDQALQHPYLR(E)
1775.8814	1775.8785	1.62	299	312	0	(K)NITCDLLEHQYWLK(G)
1864.0020	1863.9997	1.22	156	171	0	(R)IMHADLKPDNILINEK(F)
1864.0020	1863.9997	1.22	156	171	0	(R)IMHADLKPDNILINEK(F)
**Possible Modified Peptides**						
1413.8789	1413.8761	1.94	57	65	1	(K)IPVAV**K**VIR(D)
2360.2550	2360.2504	1.96	32	49	1	(R)YSVVCELVG**K**GVFSNVLK(C)

Proteins Used in this study.

Compound 4

MTTGNNANLSDNWNDSEGYYKAMVGEVIDKRYSVVCELVGKGVFSNVLKCYDMVNKIPVAVKVIRDNDMMKKA
AEKEISILKKLNQYDKDNKRHIIRLLSSIKYKNHLCLVFEWMWGNLRIALKKYGNGHGLNATAVHCYTKQLFIALRH
MRKCRIMHADLKPDNILINEKFNALKVCDLGSASDISENEITSYLVSRFYRAPEIILGFRYDAQIDVWSAAATVFELA
TGKILFPGKSNNHMIKLMMEYKGKFSHKMIKGGQFYSQHFNENLDFLYVDRDHYSKKEVVRVISDLRPTKNITCDL
LEHQYWLKGNSPKMQFLKKKIKQLGDLLEKCLILDPSKRYTPDQALQHPYLRESIHFSKSQNEGSENLYFQGHHH
HHH

Compound 9

MTTGNNANLSDNWNDSEGYYKAMVGEVIDKRYSVVCELVGKGVFSNVLKCYDMVNKIPVAVKVIRDNDMMKKA
AEKEISILKKLNQYDKDNKRHIIRLLSSIKYKNHLCLVFEWMWGNLRIALKKYGNGHGLNATAVHCYTKQLFIALRH
MRKCRIMHADLKPDNILINEKFNALKVCDLGSASDISENEITSYLVSRFYRAPEIILGFRYDAQIDVWSAAATVFELA
TGKILFPGKSNNHMIKLMMEYKGKFSHKMIKGGQFYSQHFNENLDFLYVDRDHYSKKEVVRVISDLRPTKNITCDL
LEHQYWLKGNSPKMQFLKKKIKQLGDLLEKCLILDPSKRYTPDQALQHPYLRESIHFSKSQNEGSENLYFQ
